# Synthesis and Biological Evaluation of New Compounds with Nitroimidazole Moiety

**DOI:** 10.3390/molecules29133023

**Published:** 2024-06-26

**Authors:** Katarzyna Dziduch, Sara Janowska, Sylwia Andrzejczuk, Paulina Strzyga-Łach, Marta Struga, Marcin Feldo, Oleg Demchuk, Monika Wujec

**Affiliations:** 1 Doctoral School, Medical University of Lublin, Chodzki 7, 20-093 Lublin, Poland; katarzynadziduch@o2.pl; 2 Department of Pathobiochemistry and Interdisciplinary Applications of Ion Chromatography, Biomedical Sciences, Medical University of Lublin, 1 Chodzki Street, 20-093 Lublin, Poland; sara.janowska@umlub.pl; 3 Department of Pharmaceutical Microbiology, Faculty of Pharmacy, Medical University, 20-093 Lublin, Poland; sylwia.andrzejczuk@umlub.pl; 4 Chair and Department of Biochemistry, Medical University of Warsaw, 02-097 Warszawa, Poland; paulina.strzyga-lach@wum.edu.pl (P.S.-Ł.); marta.struga@wum.edu.pl (M.S.); 5 Department of Vascular Surgery, Medical University of Lublin, Staszica 11 St., 20-081 Lublin, Poland; marcin.feldo@umlub.pl; 6 Faculty of Medicine, The John Paul II Catholic University of Lublin, Konstantynow 1J, 20-708 Lublin, Poland; oleh.demchuk@kul.lublin.pl; 7 Department of Organic Chemistry, Faculty of Pharmacy, Medical University of Lublin, 4A Chodzki Street, 20-093 Lublin, Poland

**Keywords:** synthesis, thiosemicarbazide, hydrazide-hydrazone, structure-activity relationship, antibacterial activity, anticancer activity

## Abstract

Heterocyclic compounds, particularly those containing azole rings, have shown extensive biological activity, including anticancer, antibacterial, and antifungal properties. Among these, the imidazole ring stands out due to its diverse therapeutic potential. In the presented study, we designed and synthesized a series of imidazole derivatives to identify compounds with high biological potential. We focused on two groups: thiosemicarbazide derivatives and hydrazone derivatives. We synthesized these compounds using conventional methods and confirmed their structures via nuclear magnetic resonance spectroscopy (NMR), MS, and elemental analysis, and then assessed their antibacterial and antifungal activities in vitro using the broth microdilution method against Gram-positive and Gram-negative bacteria, as well as *Candida* spp. strains. Our results showed that thiosemicarbazide derivatives exhibited varied activity against Gram-positive bacteria, with MIC values ranging from 31.25 to 1000 µg/mL. The hydrazone derivatives, however, did not display significant antibacterial activity. These findings suggest that structural modifications can significantly influence the antimicrobial efficacy of imidazole derivatives, highlighting the potential of thiosemicarbazide derivatives as promising candidates for further development in antibacterial therapies. Additionally, the cytotoxic activity against four cancer cell lines was evaluated. Two derivatives of hydrazide-hydrazone showed moderate anticancer activity.

## 1. Introduction

Despite the continuous development of medicine and pharmacology, the search for new, more effective, and less toxic drugs is still one of the most important areas of interest in medical science. This is related to the rapid development of drug resistance and low selectivity and, at the same time, significant toxicity of known therapeutic agents [1,2]. This problem applies to antibacterial, antifungal, and anticancer drugs. One of the strategies for searching for new, more effective drugs is to modify known leading pharmacophore structures, about which we know that molecules containing them have high biological activity [2,3,4]. Thanks to this method, it is possible to obtain compounds that act selectively towards selected molecular targets. As a consequence of having such a mechanism of action, the obtained molecules may exhibit higher therapeutic activity and lower toxicity [2,5].

An important class of structures used in the design of new drugs are heterocyclic groups. Many research studies have shown that heterocyclic molecules containing the azole ring system in their structure exhibit a wide spectrum of biological activity, including anticancer, antibacterial, and antifungal properties [6,7,8,9]. One of the nitrogen-based heterocyclic systems is the imidazole ring. This structure was discovered in the 1840s, arousing great interest among scientists involved in the design of new drugs [10]. This was due to the fact that compounds based on the imidazole core scaffold showed promising multidirectional effects, including anticancer [11,12], antimicrobial [6], antimycobacterial [13], analgesic [14], antidiabetic [15], antithrombotic [16], antiviral [17], antimalarial [18], anticonvulsant [19], and anti-inflammatory effects [20]. There are many drugs registered and used in clinical practice based on the imidazole core scaffold (Table 1).

The imidazole ring has structural features that increase its ability to form significant interactions with molecular targets, such as hydrogen bonds and van der Waals and hydrophobic interactions [10]. The presence of basic nitrogen atoms in the heterocyclic ring allows the development of compounds with reduced lipophilicity, which makes it possible to formulate water-soluble salts from them, which may have a beneficial effect on their pharmacokinetic properties [11]. What is also important for the significant biological activity of many imidazole derivatives is the fact that many compounds occurring in nature, such as histamine, histidine, or nucleic acids, contain this core scaffold in their structure [10,21]. As a consequence, imidazole and its derivatives have a significant impact on the course of processes in biological systems, in particular through interactions with enzymes, in which they play the role of, among others, ligands of the coordination system and proton donors or acceptors. Imidazole is also a good pharmacophore due to its ability to form π interactions with protein residues [21,22].

In our research work, we decided to design and synthesize a series of imidazole derivatives in order to find molecules with the highest biological potential. Based on scientific data proving the multidirectional action of molecules based on this heterocyclic ring, we decided to subject the obtained products to biological tests for antibacterial, antifungal, and anticancer activity.

When designing the molecules, we decided to create two groups of derivatives that differ in certain chemical properties. The first were compounds containing a thiosemicarbazide moiety, providing the molecule with the possibility of rotation. The second group consisted of molecules with a hydrazone structure, characterized by greater stiffness due to the presence of a double bond between the nitrogen and carbon atoms. By designing the synthesis in two different directions, we intended to find the relationship between structure and activity in a number of tested compounds. Both hydrazones and thiosemicarbazides constitute a group of compounds with distinctive biological properties, particularly antibacterial [23,24,25] and antifungal [26,27] activities.

In the synthetic work undertaken, using a conventional method, we obtained a number of thiosemicarbazides and hydrazones, which are imidazole derivatives. We confirmed the structure of the obtained compounds by analyzing spectra obtained by infrared spectroscopy (IR) and nuclear magnetic resonance spectroscopy (NMR), as well as by elemental analyses. The antibacterial and antifungal effects of the synthesized compounds were assessed in vitro using the broth microdilution method against both bacteria (Gram-negative and -positive) and *Candida* spp. reference strains. We then tested the cytotoxic activity of the newly synthesized compounds against cancer cell lines in vitro using the MTT assay.

## 2. Results and Discussion

### 2.1. Chemistry

**Scheme 1:**
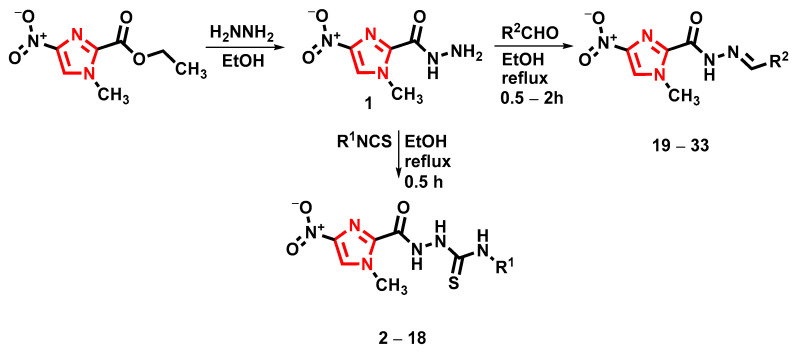
The synthetic route of thiosemicarbazide derivatives (**2**–**18**) and hydrazide-hydrazone derivatives (**19**–**33**).

The main aim of this study was to synthesize new hydrazide–hydrazones and thiosemicarbazides to investigate their in vitro antimicrobial and anticancer activities. To synthesize new derivatives, 1-methyl-4-nitroimidazolole-2-carbohydrazide (**1**) was used as a substrate by reacting it with selected isothiocyanates and derivatives of benzaldehyde. The reactions were performed according to Scheme 1. The substituents of synthesized compounds and yields of all reactions are summarized in Table 2.

The reaction between the 1-methyl-4-nitroimidazolole-2-carbohydrazide and appropriate isothiocyanate in boiling ethanol afforded thiosemicarbazides (**2**–**18**) in good yields of 81–85%. The structures of the new compounds, described for the first time, were determined using ^1^H NMR spectroscopy and elemental analyses. Compounds **2**–**13** we obtained and published previously [28]. The ^1^H NMR spectra showed chemical shifts of protons located on nitrogen atoms as three singlets: one in the range of 9.46–10.04 ppm, the second in the range of 9.72–10.20 ppm, and the third in the range of 10.82–10.99 ppm. Signals of three protons of the methyl group in position N1 imidazole ring at 3.98–4.01 ppm, appearing as a singlet, were observed.

The reaction between the 1-methyl-4-nitroimidazolole-2-carbohydrazide and appropriate aldehydes in boiling ethanol afforded hydrazide–hydrazones (**19**–**33**) in moderate-to-good yields (32–88%). Higher yields for monosubstituted benzaldehyde derivatives were obtained. The compounds (**19**–**33**) were characterized by the ^1^H and ^13^C NMR spectra. The ^1^H NMR spectra of all hydrazide–hydrazones (**19**–**33**) confirmed the proposed structures. In all the compounds, the NH peaks of hydrazone appeared at δ 12.11–12.61 ppm. The peak for the =CH group was observed at δ 8.58–9.08 ppm, confirming the formation of final products. In the ^13^C NMR spectra, the peaks for the carbon of the methyl group were present in the range δ 36.8–37.0 ppm.

^1^H NMR, ^13^C NMR, and MS spectra for the new compounds are presented in the Supplementary Materials. Additionally, ^13^C NMR spectra for previously described compounds were added to the Supplementary Materials.

### 2.2. Antibacterial and Antifungal Activity Assay

The antibacterial activity of all thiosemicarbazides and hydrazide–hydrazones was determined by evaluating their ability to inhibit microbial growth in vitro using the broth microdilution method.

The results of the tests for the antimicrobial activity of the compounds in bacteria are presented in Table 3.

None of the thiosemicarbazide (**2**–**18**) and hydrazone derivatives (**19**–**33**) showed antimicrobial activity against reference strains of Gram-negative bacteria used in in vitro tests. None of the newly synthesized imidazole derivatives containing a hydrazone moiety (**19**–**33**) showed antibacterial activity against both Gram-negative and Gram-positive bacteria; therefore, these compounds were not included in Table 3.

The tested thiosemicarbazide derivatives (**2**–**18**) differed from each other structurally in terms of the type and position of the substituent in the phenyl ring located in position 1, with respect to the thiosemicarbazide moiety. This made it possible to trace the relationship between the antimicrobial activity and structural differences in this group of compounds. A series of imidazole derivatives containing a thiosemicarbazide moiety (**2**–**18**) in their structure showed varied activity against Gram-positive bacteria with MIC values in the range of 31.25–1000 µg/mL, especially against *S. epidermidis* ATCC 12228, *M. luteus* ATCC 10240, *B. subtilis* ATCC 6633, and *B. cereus* ATCC 10876 (Table 2). Their activity was mostly bactericidal with MBC/MIC = 1–2. For compounds **4** and **5**, with a chlorophenyl moiety with a chlorine atom in the *meta* or *para* position, together with **14**, having a trifluoromethyl substituent in the *para* position in the phenyl ring, the lowest MIC concentrations in the range 31.25–62.5 µg/mL were recorded against *B. subtilis* ATCC 6633 and the ratio MBC/MIC = 1 indicated their bactericidal activity. In addition, compound **14** showed activity against the broadest spectrum of Gram-positive bacteria, including *S. aureus* ATCC 6538 and *S. aureus* MRSA ATCC 43300, with MICs ranging from 31.25 to 62.5 µg/mL and MBC ranging from 500 to 1000 µg/mL. The MBC/MIC value for these microorganisms was ≥4, indicating its bacteriostatic nature.

According to our results, two compounds, **17** and **18**, were also reported to have bactericidal activity against *S. aureus* ATCC 6538, *M. luteus* ATCC 10240, and both of the *Bacillus* spp. species, but MIC concentrations were determined to be 125 µg/mL and 500–1000 µg/mL, respectively. The MIC values for the other compounds ranged from 62.5 to 1000 µg/mL for staphylococci (*S. aureus* ATCC 25923 and *S. aureus* ATCC 6538).

The obtained results indicate the importance of the trifluoromethylphenyl group as a pharmacophore determining the antimicrobial activity in the group of thiosemicarbazide derivatives. This observation is reflected in the results of previous work carried out by our research team [23,24].

Antifungal activity tests were also carried out on the newly synthesized hydrazone and thiosemicarbazide derivatives. The results of the antifungal potential tests, in terms of MIC and MFC values, are given in Table 4. Hydrazone derivatives (**19**–**33**) were not active towards tested strains. According to our results, none of the tested compounds showed fungistatic or fungicidal activity against the reference strains of *C. glabrata* ATCC 90030, *C. glabrata* ATCC 15126, *C. krusei* ATCC 14243, *C. auris* CDC B11903, and *C. lusitaniae* ATCC 3449. Some of the obtained thiosemicarbazide derivatives (**3**, **4**, **9**, **10**, **12**, **13**, and **14**) showed antifungal activity only against *C. parapsilosis* ATCC 22019 and *C. tropicalis* ATCC 1369, with MIC and MBC values in the range of 62.5–1000 µg/mL and 500–1000 µg/mL, respectively. Their activity was mostly fungicidal (MFC/MIC = 1–2) against these abovementioned strains of *Candida* spp. The compounds differed in their substituents, which may be important for their apparent anti-yeast activity. Chlorophenyl substituents, especially *meta* and *para* chlorophenyl and methoxyphenyl (*ortho* and *para*), can be mentioned as conducive to this activity. Among the thiosemicarbazide derivatives tested (**218**), only one compound, **10**, characterized by fungistatic activity, was recorded, with MIC = 250 µg/mL and MBC = 1000 µg/mL.

Summarizing the results of the antimicrobial activity test of the tested group of thiosemicarbazide-imidazole derivatives (**2**–**18**), a high potential against a group of Gram-positive bacteria can be observed, with a lack of activity against Gram-negative bacteria. In addition, it can be noted that the addition of a trifluoromethyl substituent in the *para* position of the phenyl ring in the thiosemicarbazide compound **14** had the most favorable effect on its antimicrobial activity, both against a wide spectrum of Gram-positive bacteria (especially bacilli) and against yeast reference strains (either *C. parapsilosis* or *C. tropicalis*). For anti-yeast properties, the use of chlorophenyl as a substituent proved most beneficial.

### 2.3. Cytotoxic Evaluation

In vitro studies on normal and cancer cell lines showed the lack of cytotoxic potential of all tested compounds, except for the two compounds that are hydrazone derivatives of imidazole, compounds **30** and **31**. After 24 h of incubation, these particles showed cytotoxic activity against the human metastatic colon cancer SW620 cell line, with IC_50_ of 64.2 µM and 86.3 µM, respectively. The same hydrazone derivatives of imidazole were also active against the human metastatic prostate cancer PC3 cell line, with IC_50_ values of 70.2 µM and 70.4 µM, respectively. None of the tested compounds showed cytotoxic potential against the estrogen-independent breast cancer MDA-MB-231 and A-549 throat cancer cell lines.

Although these compounds had a much lower potential in tests than the reference drugs, they showed high selectivity of cytotoxic activity directed against cancer cells. The cytotoxic activity of **30** (Table 1) against normal cells was at the IC_50_ level of 97.0 µM, while the value for **31** was >100 µM. For comparison, the toxic effect of doxorubicin on normal cells was at the IC_50_ level of 0.3 µM, and in the case of cisplatin, at the level of 6.3 µM. This means that the selectivity index in our tests is much more favorable for the newly synthesized **30** and **31** molecules than for the reference drugs used. The results of the tests performed are presented in Table 5.

In terms of chemical structure, compounds **30** and **31** were distinguished by the presence of an aromatic ring with a hydroxyl substituent in position 2 and a methoxy group in positions 4 and 5, respectively. Interestingly, the compounds **32** and **33**, having a hydroxyl group in position 4 and methoxy groups in position 3 and 2, respectively, did not show cytotoxic activity in in vitro tests.

## 3. Materials and Methods

### 3.1. General Comments

All the chemicals utilized in this research were obtained from Sigma-Aldrich (Munich, Germany) and employed without further purification. The ^1^H NMR and ^13^C NMR spectra were acquired on a Bruker Avance 600 spectrometer (Bruker BioSpin GmbH, Rheinstetten, Germany) in DMSO-d_6_. The HPLC and MS analyses were performed on a Shimadzu LCMS Q-Tof 9030 ESI (Kioto, Japan) instrument equipped with a column Dr. Maisch ReproSil-Pur Basic-C18 3 µm 100 mm which was eluted at 40 °C by MeOH/H_2_O = 70/30 with rate 0.3 mL/min, and the injection of analyte were applied at the first minute of elution. The NMR and MS spectra can be found in the Supplementary Materials. Melting points were measured with a Fisher-Johns melting point apparatus (Fisher Scientific, Schwerte, Germany) and were uncorrected. The purity of the compounds and the reaction progress were tracked by TLC using an aluminum sheet with 60 F_254_ plates (Merck Co., Kenilworth, NJ, USA) with a CHCl_3_/EtOH (10:1, *v*/*v*) solvent system. Elemental analyses were conducted using a Perkin Elmer 2400 Series II CHNS/O analyzer (Waltham, MA, USA), with results within ±0.4% of the theoretical values.

#### 3.1.1. Synthesis of Thiosemicarbazide Derivatives

The procedure for the synthesis of 1-[(1-methyl-4-nitroimidazol-2-yl)carbonyl]-4-substituted-thiosemicarbazide was described previously by us [28].

1-[(1-methyl-4-nitroimidazol-2-yl)carbonyl]-4-[(4-trifluoromethylphenyl)thiosemicarbazide (**14**)

Light yellow powder. m.p.: 204–206 °C. ^1^H NMR (600 MHz, DMSO-*d*_6_) δ (ppm): 4.00 (s, 3H, CH_3_), 7.68 (d, 2H, Ar-H, *J* = 8.6 Hz), 7.76 (d, 2H, Ar-H, *J* = 8.6 Hz), 8.66 (s, 1H, CH), 9.92; 10.04; 10.94 (3s, 3H, 3NH). ^13^C NMR (151 MHz, DMSO-d_6_) δ (ppm): 36.8; 122.09; 124.8 (q, *J* = 213.8 Hz); 125.5; 126.9; 137.1; 137.6; 143.4; 144.9; 157.0; 158.1; 181.2. HRMS (ESI) *m*/*z* = calc. for C_13_H_11_N_6_O_3_F_3_S 388.05654, mass found [M + H]^+^ 389.06348. Anal. calc. for C_13_H_11_N_6_O_3_F_3_S (%): C 40.21; H 2.86; N 21.64. Found: C 40.35; H 2.96; N 21.78.

1-[(1-methyl-4-nitroimidazol-2-yl)carbonyl]-4-[(2-methylphenyl)thiosemicarbazide (**15**)

Light yellow powder. m.p.: 164–166 °C. ^1^H NMR (600 MHz, DMSO-*d*_6_) δ (ppm): 3.98 (s, 3H, CH_3_), 7.07–7.37 (m, 4H, Ar-H), 8.62 (s, 1H, Ar-H), 9.46; 9.72; 10.88 (3s, 3H, 3NH). ^13^C NMR (151 MHz, DMSO-d_6_) δ (ppm): 18.1; 36.7; 126.2; 126.7; 127.1; 129.3; 130.3; 136.4; 137.5; 138.4, 144.8; 158.0; 181.8.HRMS (ESI) *m*/*z* = calc. for C_13_H_14_N_6_O_3_S 334.08481, mass found [M + H]^+^ 335.09177. Anal. calc. for C_13_H_14_N_6_O_3_S (%): C 46.70; H 4.22; N 25.14. Found: C 46.86; H 4.32; N 25.02.

1-[(1-methyl-4-nitroimidazol-2-yl)carbonyl]-4-(3-methylphenyl)-thiosemicarbazide (**16**)

White powder. m.p.: 201–204 °C. ^1^H NMR (600 MHz, DMSO-*d*_6_) δ (ppm): 2.28 (s, 3H, CH_3_), 3.99 (s, 3H, CH_3_), 6.95–6.98 (m, 1H, Ar-H), 7.17–7.28 (m, 3H, Ar-H), 8.64 (s, 1H, Ar-H), 9.64; 9.75; 10.83 (3s, 3H, 3NH). ^13^C NMR (151 MHz, DMSO-d_6_) δ (ppm): 21.4; 36.8; 123.3; 126.1; 126.8; 128.2; 130.1; 137.3; 137.6; 139.5; 144.8; 158.0; 181.2. HRMS (ESI) *m*/*z* = calc. for C_13_H_14_N_6_O_3_S 334.08481, mass found [M + H]^+^ 335.09156. Anal. calc. for C_13_H_14_N_6_O_3_S (%): C 46.70; H 4.22; N 25.14. Found: C 47.04; H 4.08; N 25.32.

1-[(1-methyl-4-nitroimidazol-2-yl)carbonyl]-4-(4-methylphenyl)thiosemicarbazide (**17**)

Cream powder. m.p.: 190–192 °C. ^1^H NMR (600 MHz, DMSO-*d*_6_) δ (ppm): 2.27 (s, 3H, CH_3_), 3.99 (s, 3H, CH_3_), 7.12 (d, 2H, Ar-H, *J* = 8.2 Hz), 7.29 (d, 2H, Ar-H, *J* = 8.0 Hz), 8.64 (s, 1H, Ar-H), 9.63; 9.72; 10.82 (3s, 3H, 3NH). ^13^C NMR (151 MHz, DMSO-d_6_) δ (ppm): 21.0; 36.8; 126.1; 126.8; 128.9; 134.6; 137.0; 137.3; 144.8; 158.0; 181.3. HRMS (ESI) *m*/*z* = calc. for C_13_H_14_N_6_O_3_S 334.08481, mass found [M + H]^+^ 335.09167. Anal. calc. for C_13_H_14_N_6_O_3_S (%): C 46.70; H 4.22; N 25.14. Found: C 46.98; H 4.30; N 25.26.

1-[(1-methyl-4-nitroimidazol-2-yl)carbonyl]-4-(4-nitrophenyl)-thiosemicarbazide (**18**)

Yellow powder. m.p.: 203–205 °C. ^1^H NMR (600 MHz, DMSO-*d*_6_) δ (ppm): 4.01 (s, 3H, CH_3_), 7.89 (d, 2H, Ar-H, *J* = 9.1 Hz), 8.21 (d, 2H, Ar-H, *J* = 9.2 Hz), 8.67 (s, 1H, Ar-H), 10.04; 10.20; 10.99 (3s, 3H, 3NH). ^13^C NMR (151 MHz, DMSO-d_6_) δ (ppm): 36.8; 124.1; 125.7; 127.0; 137.0; 143.9; 144.9; 146.0; 158.1; 181.0. HRMS (ESI) *m*/*z* = calc. for C_12_H_11_N_7_O_5_S 365.05424, mass found [M + H]^+^ 366.06104. Anal. calc. for C_12_H_11_N_7_O_5_S (%): C 39.45; H 3.04; N 26.84. Found: C 39.12; H 2.98; N 27.14.

#### 3.1.2. Synthesis of Hydrazide-Hydrazone Derivatives

The new hydrazone-hydrazides derivatives were prepared using the method described previously [29,30]. We started by dissolving 0.01 mole of 1-methyl-4-nitroimidazole-2-carbohydrazide (**1**) in 5 mL of 96% ethanol. Next, 0.011 mole of the appropriate substituted benzaldehyde was added, and the mixture was heated under reflux for 0.5 to 2 h. The reaction time is provided below in the description of individual compounds. After heating, the solution was cooled to room temperature. The resulting precipitate was then filtered and recrystallized from 96% ethanol.

1-methyl-4-nitro-N’-(phenylmethylidene)-1*H*-imidazole-2-carbohydrazide (**19**)

White powder. m.p.: 280–284 °C. The reaction time: ½ h. ^1^H NMR (600 MHz, DMSO-*d*_6_) δ (ppm): 4.06 (s, 3H, CH_3_), 7.46–7.49 (m, 3H, Ar-H), 7.71–7.73 (m, 2H, Ar-H), 8.63 (s, 1H, Ar-H), 8.67 (s, 1H, CH), 12.29 (s, 1H, NH). ^13^C NMR (151 MHz, DMSO-d_6_) δ (ppm): 36.9; 127.3; 127.7; 129.3; 130.8; 134.6; 137.3; 144.8; 150.1; 154.7. HRMS (ESI) *m*/*z* = calc. for C_12_H_11_N_5_O_3_ 273.08619, mass found [M + H]^+^ 274.09288. Anal. calc. for C_12_H_11_N_5_O_3_ (%): C 52.75; H 4.06; N 25.63. Found: C 52.56; H 3.85; N 25.87.

N’-[(2-chlorophenyl)methylidene]-1-methyl-4-nitro-1*H*-imidazole-2-carbohydrazide (**20**)

Light pink powder. m.p.: 234–236 °C. The reaction time: 2 h. ^1^H NMR (600 MHz, DMSO-*d*_6_) δ (ppm): 4.06 (s, 3H, CH_3_), 7.44–7.48 (m, 2H, Ar-H), 7.55 (dd, 1H, Ar-H, *J* = 7.7 Hz; 1.5 Hz), 8.01 (dd, 1H, Ar-H, *J* = 7.6 Hz; 2.0 Hz), 8.68 (s, 1H, Ar-H), 9.08 (s, 1H, CH), 12.59 (s, 1H, NH). ^13^C NMR (151 MHz, DMSO-d_6_) δ (ppm): 37.0; 127.3; 127.4; 128.1; 130.4; 132.0; 132.2; 134.0; 137.1; 144.8; 146.1; 154.8. HRMS (ESI) *m*/*z* = calc. for C_12_H_10_ClN_5_O_3_ 307.04722, mass found [M + H]^+^ 308.05423. Anal. calc. for C_12_H_10_ClN_5_O_3_ (%): C 46.84; H 3.28; N 22.76. Found: C 46.65; H 3.56; N 23.07.

N’-[(3-chlorophenyl)methylidene]-1-methyl-4-nitro-1*H*-imidazole-2-carbohydrazide (**21**)

White powder. m.p.: 276–278 °C. The reaction time: 1 h. ^1^H NMR (600 MHz, DMSO-*d*_6_) δ (ppm): 3.97 (s, 3H, CH_3_), 7.40–7.44 (m, 2H, Ar-H), 7.57 (dt, 1H, Ar-H, *J* = 6.6 Hz; 1.8 Hz), 7.66–7.67 (m, 1H, Ar-H), 8.51 (s, 1H, Ar-H), 8.58 (s, 1H, CH), 12.33 (s, 1H, NH). ^13^C NMR (151 MHz, DMSO-d_6_) δ (ppm): 37.0; 126.4; 126.8; 127.4; 130.4; 131.2; 134.1; 136.8; 137.1; 144.8; 148.3; 154.8. HRMS (ESI) *m*/*z* = calc. for C_12_H_10_ClN_5_O_3_ 307.04722, mass found [M + H]^+^ 308.05430. Anal. calc. for C_12_H_10_ClN_5_O_3_ (%): C 46.84; H 3.28; N 22.76. Found: C 47.16; H 3.34; N 22.53.

N’-[(2-fluorophenyl)methylidene]-1-methyl-4-nitro-1*H*-imidazole-2-carbohydrazide (**22**)

Cream powder. m.p.: 214–217 °C. The reaction time: 2 h. ^1^H NMR (600 MHz, DMSO-*d*_6_) δ (ppm): 4.06 (s, 3H, CH_3_), 7.30–7.33 (m, 2H, Ar-H), 7.50–7.54 (m, 1H, Ar-H), 7.94 (td, 1H, Ar-H, *J* = 7.6 Hz; 1.8 Hz), 8.68 (s, 1H, Ar-H), 8.89 (s, 1H, CH), 12.48 (s, 1H, NH). ^13^C NMR (151 MHz, DMSO-d_6_) δ (ppm): 37.0; 116.6; 122.2 (d, *J* = 9.8 Hz); 125.4; 126.8; 127.4; 132.8; (d, *J* = 8.5 Hz); 137.1; 142.9; 144.8; 154.8; 160.5; 162.2. HRMS (ESI) *m*/*z* = calc. for C_12_H_10_FN_5_O_3_ 291.07677, mass found [M + H]^+^ 292.08392. Anal. calc. for C_12_H_10_FN_5_O_3_ (%): C 49.49; H 3.46; N 24.05. Found: C 49.05; H 3.68; N 23.97.

N’-[(2-methoxyphenyl)methylidene]-1-methyl-4-nitro-1*H*-imidazole-2-carbohydrazide (**23**)

Light yellow powder. m.p.: 205–208 °C. The reaction time: 2 h. ^1^H NMR (600 MHz, DMSO-*d*_6_) δ (ppm): 3.82 (s, 3H, OCH_3_), 4.05 (s, 3H, CH_3_), 7.03–7.05 (m, 1H, Ar-H), 7.26–7.27 (m, 2H, Ar-H), 7.39 (t, 1H, Ar-H, *J* = 8.1 Hz), 8.59 (s, 1H, Ar-H), 8.67 (s, 1H, Ar-H), 8.67 (s, 1H, CH), 12.31 (s, 1H, NH). ^13^C NMR (151 MHz, DMSO-d_6_) δ (ppm): 36.9; 56.2; 112.4; 121.4; 122.7; 126.0; 127.3; 132.3; 137.3; 144.8; 145.7; 154.7; 158.4. HRMS (ESI) *m*/*z* = calc. for C_13_H_13_N_5_O_4_ 303.09675, mass found [M + H]^+^ 304.10331. Anal. calc. for C_13_H_13_N_5_O_4_ (%): C 51.49; H 4.32; N 23.09. Found: C 51.38; H 4.27; N 22.98.

N’-[(3-methoxyphenyl)methylidene]-1-methyl-4-nitro-1*H*-imidazole-2-carbohydrazide (**24**)

White powder. m.p.: 208–210 °C. The reaction time: 1 h. ^1^H NMR (600 MHz, DMSO-*d*_6_) δ (ppm): 3.82 (s, 3H, OCH_3_), 4.06 (s, 3H, CH_3_), 7.03–7.05 (m, 1H, Ar-H), 7.26–7.27 (m, 2H, Ar-H), 7.39 (t, 1H, Ar-H, *J* = 8.0 Hz), 7.66 (d, 1H, Ar-H, *J* = 8.8 Hz), 8.59 (s, 1H, Ar-H), 8.67 (s, 1H, CH), 12.30 (s, 1H, NH). ^13^C NMR (151 MHz, DMSO-d_6_) δ (ppm): 36.9; 55.6; 110.8; 117.0; 120.7; 127.4; 130.0; 136.0; 137.2; 144.8; 149.9; 154.7; 160.0. HRMS (ESI) *m*/*z* = calc. for C_13_H_13_N_5_O_4_ 303.09675, mass found [M + H]^+^ 304.10343. Anal. calc. for C_13_H_13_N_5_O_4_ (%): C 46.23; H 3.58; N 20.74. Found: C 46.05; H 3.75; N 20.38.

N’-[(4-methoxyphenyl)methylidene]-1-methyl-4-nitro-1*H*-imidazole-2-carbohydrazide (**25**)

Yellow crystal. m.p.: 224–226 °C. The reaction time: ½ h. ^1^H NMR (600 MHz, DMSO-*d*_6_) δ: (ppm) 3.82 (s, 3H, CH_3_), 4.05 (s, 3H, OCH_3_), 7.03 (d, 2H, Ar-H, *J* = 8.8 Hz), 7.66 (d, 2H, Ar-H, *J* = 8.8 Hz), 8.55 (s, 1H, Ar-H), 8.66 (s, 1H, CH), 12.15 (s, 1H, NH). ^13^C NMR (151 MHz, DMSO-d_6_) δ (ppm): 36.9; 55.8; 114.8; 127.1; 127.2; 129.3; 137.4; 144.8; 149.9; 154.5; 161.5. HRMS (ESI) *m*/*z* = calc. for C_13_H_13_N_5_O_4_ 303.09675, mass found [M + H]^+^ 304.10360. Anal. calc. for C_13_H_13_N_5_O_4_ (%): C 51.49; H 4.32; N 23.09. Found: C 51.23; H 4.45; N 22.98.

1-methyl-4-nitro-N’-[(4-trifluoromethylphenyl)methylidene]-1*H*-imidazole-2-carbohydrazide (**26**)

White powder. m.p.: 208–210 °C. The reaction time: 2 h. ^1^H NMR (600 MHz, DMSO-*d*_6_) δ (ppm): 4.06 (s, 3H, CH_3_), 7.84 (d, 2H, Ar-H, *J* = 8.2 Hz), 7.93 (d, 2H, Ar-H, *J* = 8.2 Hz), 8.69 (s, 1H, Ar-H), 8.70 (s, 1H, CH), 12.49 (s, 1H, NH). ^13^C NMR (151 MHz, DMSO-d_6_) δ (ppm): 37.0; 124.5 (d, *J* = 272.2 Hz); 125.4; 126.2; 127.5; 128.3; 130.4 (d, *J* = 31.8 Hz); 137.1; 138.5; 144.9; 148.3; 154.9. HRMS (ESI) *m*/*z* = calc. for C_13_H_10_F_3_N_5_O_3_ 341.07357, mass found [M + H]^+^ 342.08072. Anal. calc. for C_13_H_10_F_3_N_5_O_3_ (%): C 45.76; H 2.95; N 20.52. Found: C 45.23; H 2.87; N 20.25.

N’-[(3-hydroxyphenyl)methylidene]-1-methyl-4-nitro-1*H*-imidazole-2-carbohydrazide (**27**)

Dirty yellow powder. m.p.: 208–210 °C. The reaction time: 1 h. ^1^H NMR (600 MHz, DMSO-*d*_6_) δ (ppm): 4.05 (s, 3H, CH_3_), 6.84–6.86 (m, 1H, Ar-H), 7.08–7.09 (m, 1H, Ar-H), 7.17–7.18 (m, 1H, Ar-H), 7.27 (t, 1H, Ar-H), 8.53 (s, 1H, Ar-H), 8.67 (s, 1H, CH), 9.67 (s, 1H, OH), 12.22 (s, 1H, NH). ^13^C NMR (151 MHz, DMSO-d_6_) δ (ppm): 36.9; 113.3; 118.2; 119.4; 126.8; 130.4; 135.8; 137.2; 144.8; 150.2; 154.7; 158.0. HRMS (ESI) *m*/*z* = calc. for C_12_H_11_N_5_O_4_ 289.08110, mass found [M + H]^+^ 290.08798. Anal. calc. for C_12_H_11_N_5_O_4_ (%): C 49.83; H 3.83; N 24.21. Found: C 49.78; H 3.92; N 24.25.

N’-[(3-chloro-2-fluorophenyl)methylidene]-1-methyl-4-nitro-1*H*-imidazole-2-carbohydrazide (**28**)

Dirty yellow powder. m.p.: 252–255 °C. The reaction time: 2 h. ^1^H NMR (600 MHz, DMSO-*d*_6_) δ (ppm): 4.06 (s, 3H, CH_3_), 7.34 (t, 1H, Ar-H, *J* = 8.0 Hz), 7.68–7.70 (m, 1H, Ar-H), 7.87–7.90 (m, 1H, Ar-H), 8.69 (s, 1H, Ar-H), 8.89 (s, 1H, CH), 12.57 (s, 1H, NH). ^13^C NMR (151 MHz, DMSO-d_6_) δ (ppm): 37.0; 120.8 (d, *J* = 17 Hz); 124.0 (d, *J* = 9.8 Hz); 125.7; 126.2; 127.4; 132.5; 136.9; 142.1; 144.8; 154.8; 155.6; 157.2. HRMS (ESI) *m*/*z* = calc. for C_12_H_9_ClFN_5_O_3_ 325.03780, mass found [M + H]^+^ 326.04462. Anal. calc. for C_12_H_9_ClFN_5_O_3_ (%): C 44.26; H 2.79; N 21.50. Found: C 44.62; H 2.65; N 21.32.

N’-[(4-chloro-2-methoxyphenyl)methylidene]-1-methyl-4-nitro-1*H*-imidazole-2-carbohydrazide (**29**)

Light yellow fluffy powder. m.p.: 234–237 °C. The reaction time: 1 h. ^1^H NMR (600 MHz, DMSO-*d*_6_) δ (ppm): 3.90 (s, 3H, OCH_3_), 4.04 (s, 3H, CH_3_), 7.10 (dd, 1H, Ar-H, *J* = 8.4; 1.9 Hz), 7.22 (d, 1H, Ar-H, *J* = 2.0 Hz), 7.22 (d, 1H, Ar-H, *J* = 8.4 Hz), 8.66 (s, 1H, Ar-H), 8.91 (s, 1H, CH), 12.41 (s, 1H, NH). ^13^C-NMR (151, MHz, DMSO-d_6_) δ (ppm): 36.9; 56.7; 112.8; 121.4; 121.7; 127.3; 127.3; 136.5; 137.2; 144.7; 144.8; 154.7; 159.0. HRMS (ESI) *m*/*z* = calc. for C_13_H_12_ClN_5_O_4_ 337.05778, mass found [M + H]^+^ 338.06457. Anal. calc. for C_13_H_12_ClN_5_O_4_ (%): C 51.49; H 4.32; N 23.09. Found: C 51.64; H 4.66; N 23.47.

N’-[(2-hydroxy-4-methoxyphenyl)methylidene]-1-methyl-4-nitro-1*H*-imidazole-2-carbohydrazide (**30**)

Yellow powder. m.p.: 214–216 °C. The reaction time: 2 h. ^1^H NMR (600 MHz, DMSO-*d*_6_) δ (ppm): 3.79 (s, 3H, CH_3_), 3.99 (s, 1H, OH), 4.06 (s, 3H, OCH_3_), 6.50 (d, 1H, Ar-H, *J* = 2.5 Hz), 6.54 (dd, 1H, Ar-H, *J* = 8.6 Hz; 2.5 Hz), 7.39 (d, 1H, Ar-H, *J* = 8.6 Hz), 8.67 (s, 1H, Ar-H), 8.72 (s, 1H, CH), 11.54 (s, 1H, OH), 12.52 (s, 1H, NH). ^13^C NMR (151 MHz, DMSO-d_6_) δ (ppm): 37.0; 55.8; 101.6; 107.1; 112.1; 127.4; 131.9; 136.9; 144.9; 151.1; 154.3; 160.0; 162.8. HRMS (ESI) *m*/*z* = calc. for C_13_H_13_N_5_O_5_ 319.09167, mass found [M + H]^+^ 320.09884. Anal. calc. for C_13_H_13_N_5_O_5_ (%): C 48.91; H 4.10; N 21.94. Found: C 49.02; H 4.32; N 21.63.

N’-[(2-hydroxy-5-methoxyphenyl)methylidene]-1-methyl-4-nitro-1*H*-imidazole-2-carbohydrazide (**31**)

Light yellow powder. m.p.: 236–238 °C. The reaction time: 1 h. ^1^H NMR (600 MHz, DMSO-*d*_6_) δ (ppm): 3.74 (s, 3H, OCH_3_), 4.06 (s, 3H, NH), 6.87 (d, 1H, Ar-H, *J* = 8,9 Hz), 6.93 (dd, 1H, Ar-H, *J* = 8.9 Hz; 3.1 Hz), 7.08 (d, 1H, Ar-H, *J* = 3.1 Hz), 8.68 (s, 1H, Ar-H), 8.79 (s, 1H, CH), 10.62 (s, 1H, OH), 12.61 (s, 1H, NH). ^13^C NMR (151 MHz, DMSO-d_6_) δ (ppm): 36.9; 55.9; 112.6; 117.8; 119.2; 119.3; 127.4; 136.9; 144.9; 149.8; 152.0; 152.6; 154.6. HRMS (ESI) *m*/*z* = calc. for C_13_H_13_N_5_O_5_ 319.09167, mass found [M + H]^+^ 320.09864. Anal. calc. for C_13_H_13_N_5_O_5_ (%): C 48.91; H 4.10; N 21.94. Found: C 49.23; H 3.96; N 22.02.

N’-[(4-hydroxy-3-methoxyphenyl)methylidene]-1-methyl-4-nitro-1*H*-imidazole-2-carbohydrazide (**32**)

Intense yellow powder. m.p.: 241–243 °C. The reaction time: 1 h. ^1^H NMR (600 MHz, DMSO-*d*_6_) δ (ppm): 3.84 (s, 3H, OCH_3_), 4.04 (s, 3H, CH_3_), 6.85 (d, 1H, Ar-H, *J* = 8,1 Hz), 7.07 (dd, 1H, Ar-H, *J* = 8.2 Hz; 1.9 Hz), 7.30 (d, 1H, Ar-H, *J* = 1.8 Hz), 8.48 (s, 1H, Ar-H), 8.64 (s, 1H, CH), 9.61 (s, 1H, OH), 12.11 (s, 1H, NH). ^13^C NMR (151 MHz, DMSO-d_6_) δ (ppm): 36.9; 56.0; 109.5; 115.9; 123.0; 125.9; 127.7; 137.4; 144.8; 148.5; 149.7; 150.6; 154.5. HRMS (ESI) *m*/*z* = calc. for C_13_H_13_N_5_O_5_ 319.09167, mass found [M + H]^+^ 320.09868. Anal. calc. for C_13_H_13_N_5_O_5_ (%): C 48.91; H 4.10; N 21.94. Found: C 49.24; H 3.98; N 21.76.

N’-[(4-hydroxy-2-methoxyphenyl)methylidene]-1-methyl-4-nitro-1*H*-imidazole-2-carbohydrazide (**33**)

Yellow powder. m.p.: 233–235 °C. The reaction time: 2 h. ^1^H NMR (600 MHz, DMSO-*d*_6_) δ (ppm): 3.80 (s, 3H, OCH_3_), 4.03 (s, 3H, CH_3_), 6.44–6.46 (m, 2H, Ar-H), 7.69 (d, 1H, Ar-H, *J* = 8.9 Hz), 8.63 (s, 1H, Ar-H), 8.83 (s, 1H, CH), 10.05 (s, 1H, OH), 12.14 (s, 1H, NH). ^13^C NMR (151 MHz, DMSO-d_6_) δ (ppm): 36.8; 56.0; 99.4; 108.8; 113.8; 127.1; 127.4; 137.5; 144.8; 146.2; 154.4; 160.1; 161.7. HRMS (ESI) *m*/*z* = calc. for C_13_H_13_N_5_O_5_ 319.09167, mass found [M + H]^+^ 320.09884. Anal. calc. for C_13_H_13_N_5_O_5_ (%): C 48.91; H 4.10; N 21.94. Found: C 49.16; H 4.28; N 20.15.

### 3.2. Microbiology

The antibacterial activity of the obtained thiosemicarbazides and hydrazide–hydrazones was determined by evaluating their ability to inhibit microbial growth in vitro using the broth microdilution method, according to the guidelines of the European Committee on Antimicrobial Susceptibility Testing (EUCAST) [31]. The method involved the preparation of microbial suspensions with a standardized density of 0.5 on the McFarland scale (equivalent to 1.5 × 10^8^ colony-forming units/mL for bacteria and 1.5 × 10^8^ CFU/mL for fungi). A solution of the test substances was then prepared (initial concentration 1000 µg/mL, diluted in sterile Mueller-Hinton broth for bacteria or Mueller-Hinton with 2% glucose for fungi). The test was carried out in 96-well polystyrene flat-bottom plates (Medlab, Poland) with 100 µL of appropriate sterile microbiological medium in each well. The first wells were filled with 200 µL of the test substance, diluted to the initial concentration, and then double-decreasing dilutions were made under aseptic conditions using an automatic pipette. The final step was the addition of a 10-fold diluted microbial suspension of 2 µL. At the same time, the following controls were included: (a) medium control (100 µL of sterile medium without microorganisms added), (b) microbial viability control, (c) test substance control (dilutions without added micro-organisms), (d) positive control of the drug used in the clinic. The plates thus prepared were incubated for 18 ± 2 h at 35 ± 2 °C under conditions optimal for the growth of the test microorganisms. After incubation, the spectrophotometric absorbance at 570 nm in each well of the plate was read. The aim of this part of the study was to determine two parameters: the minimum inhibitory concentration (MIC) and the minimum bactericidal or fungicidal concentration (MBC/MFC) of the test compound. The MIC value was determined in the well where the absorbance was significantly lower than the absorbance in the next well with a lower concentration. The MBC value was determined by adding 5 µL of the contents of each well of the plate to solid sterile MHA or MHA + 2%glc medium for bacteria and fungi, respectively. The area on the plate where 99.9% inhibition of microbial growth was observed indicated the lethal concentration. In addition, the quotient MBC(MFC)/MIC could be determined to assess the nature of the activity of the test substance. If MBC(MFC)/MIC was <4, the test substance was bactericidal/fungicidal, whereas if MBC(MFC)/MIC was ≥4, it was bacteriostatic/fungistatic. Amoxicillin (Glentham Life Sciences, USA) and nystatin (Glentham Life Sciences, USA) were used as the controls for this assay.

The test was performed either on bacteria, comprising: Gram-positive (*Staphylococcus aureus* ATCC 25923, *Staphylococcus aureus* ATCC 6538, *Staphylococcus aureus* ATCC 43300 methicillin-resistant MRSA, *Staphylococcus epidermidis* ATCC 12228, *Micrococcus luteus* ATCC 10240, *Bacillus subtilis* ATCC 6633, *Bacillus cereus* ATCC 10876) and Gram-negative (*Escherichia coli* ATCC 25922, *Pseudomonas aeruginosa* ATCC 27853, *Proteus mirabilis* ATCC 12453, *Klebsiella pneumoniae* ATCC 13883) or yeast (*Candida parapsilosis* ATCC 22019, *Candida glabrata* ATCC 90030, *Candida krusei* ATCC 14243, *Candida glabrata* ATCC 15126, *Candida tropicalis* ATCC 1369, *Candida auris* CDC B11903, *Candida lusitaniae* ATCC 3449) reference strains from the American Type Culture Collection (ATCC) or the Centers for Disease Control and Prevention (CDC).

### 3.3. In Vitro Cytotoxicity Determination

#### 3.3.1. Cell Cultures

Colon cancer (SW480), breast adenocarcinoma (MDA-MB-231), lung carcinoma (A-549), prostate cancer (PC3), and immortal keratinocyte (HaCaT) cell lines of human origin were acquired from the American Type Culture Collection (ATCC). The recommended culture media and protocols provided by ATCC were as follows: SW480 were cultured in Minimum Essential Medium (MEM), MDA-MB-231, A-549, and HaCaT cells were cultured in Dulbecco’s Modified Eagle Medium (DMEM), while PC3 was cultured in Roswell Park Memorial Institute Medium (RPMI). The culture media were supplemented with 10% fetal bovine serum (FBS), penicillin (100 U/mL), and streptomycin (100 μg/mL). Cells were maintained in a humidified incubator at 37 °C with 5% CO_2_. Passaging was carried out upon reaching 80–90% confluence using 0.25% trypsin (Gibco Life Technologies, New York, NY, USA) treatment prior to experimentation.

#### 3.3.2. MTT Tests

Cell viability was assessed utilizing the MTT assay, which relies on the conversion of 3-(4,5-dimethylthiazol-2-yl)-2,5-diphenyltetrazolium bromide salt (MTT) to formazan crystals by mitochondrial dehydrogenases in viable cells. Initially, cells were plated in 96-well plates at a density of 1 × 10^4^ cells per well and allowed to attach for 24 h at 37 °C in a CO_2_ incubator. Subsequently, the culture medium was replaced with fresh medium containing various concentrations of the test compounds (ranging from 1 to 100 μM), and cells were further cultured for 24 h at 37 °C in a CO_2_ incubator. Control wells were left untreated for comparison. After the incubation period, MTT solution (0.5 mg/mL in serum-free medium) was added to each well, followed by a 4-h incubation at 37 °C in a CO_2_ incubator. The medium was then aspirated, and the formazan crystals were dissolved by adding a mixture of isopropanol and DMSO (1:1). The absorbance of the resulting solution was measured at 570 nm using a UVM 340 reader (ASYS Hitech GmbH, Seekirchen am Wallersee, Austria). IC_50_ values were determined using GraphPad software (GraphPad Prism 8 Software).

## 4. Conclusions

In summary, two groups of compounds with a different selective spectra of activity can be distinguished in the pool of compounds tested. One of them is imidazole derivatives with a thiosemicarbazide moiety in the structure with an aromatic ring with a halogen substituent attached in the *para* position (**5**, **8**, **14**). Compounds from this group showed variable antimicrobial activity, either against some *Candida* spp. reference strains, including *C. parapsilosis* and *C. tropicalis*, or against Gram-positive bacteria, comprising staphylococci, micrococci, and bacilli. The most active compound, with the broadest spectrum of antimicrobial activity, **14**, containing a thiosemicarbazide moiety with a 4-trifluoromethylphenyl substituent, showed predominantly bacteriostatic activity against all Gram-positive bacteria tested, with MIC values ranging from 31.25 to 62.5 µg/mL, except for *B. subtilis*, against which this compound was bactericidal with a rather low MIC = 31.25 µg/mL. The main advantage of the newly synthesized bioactive thiosemicarbazides is the selectivity of their antimicrobial activity. The results obtained for **3**, **4**, **9**, **10, 12**, **13**, and **14** in terms of activity against *C. tropicalis*, a yeast species other than *C. albicans* which is of increasing clinical importance, also appear to be promising. All compounds from this group of imidazole derivatives showed very low toxicity to normal human cells in in vitro tests.

The second group that can be distinguished in the pool of compounds tested are hydrazones, which exhibit selective anticancer activity against the PC3 prostate cancer and SW620 intestinal cancer cell lines. These particles did not show any bactericidal activity and showed very low cytotoxic activity toward normal human cells. This high selectivity towards cancer cells combined with low toxicity makes these molecules potential lead-hit structures for further development of new anticancer agents in the group of imidazole derivatives.

## Figures and Tables

**Table 1:** Drugs containing an imidazole system in their structure.

Drug	Structural Formula	Activity
*Metronidazole*	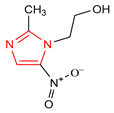	Antibacterial antiprotozoal
*Ornidazole*	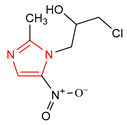	antibacterial antiprotozoal
*Tinidazole*	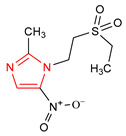	antibacterial antiprotozoal
*Nimorazole*	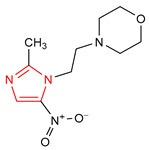	antibacterial antiprotozoal
*Benznidazole*	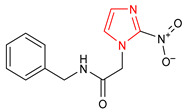	antibacterial antiprotozoal
*Etomidate*	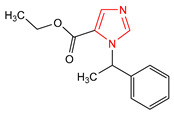	anesthetic
*Pilocarpine*	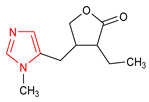	parasympathomimetic
*Orazamide*	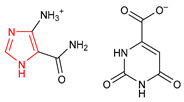	hepatoprotective
*Ondansetron*	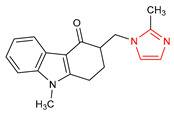	antiemetic
*Alosetrone*	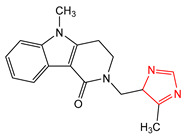	IBS treatment
*Clotrimazole*	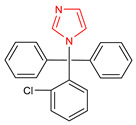	antifungal
*Bifonazole*	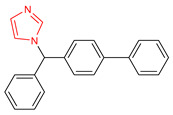	antifungal
*Miconazole*	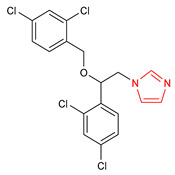	antifungal
*Econazole*	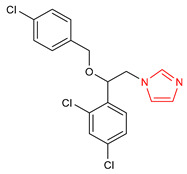	antifungal
*lsoconazole*	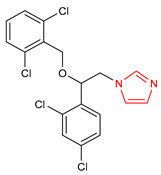	antifungal
*Tioconazole*	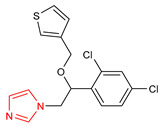	antifungal
*Sertaconazole*	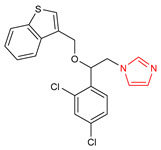	antifungal
*Fenticonazole*	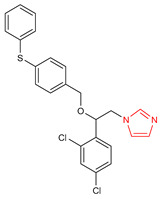	antifungal
*Ketoconazole*	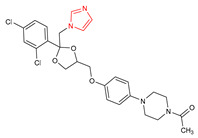	antifungal
*Oxlconazole*	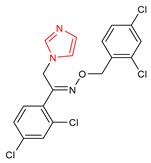	antifungal
*Omoconazole*	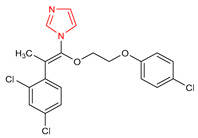	antifungal
*Croconazole*	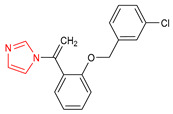	antifungal
*Butoconazole*	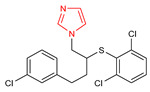	antifungal
*Cymetidine*	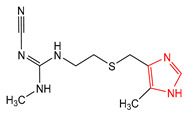	antihistamines
*Losartane*	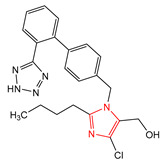	angiotensin receptor AT_1_ antagonist, lowering blood pressure
*Eprosartane*	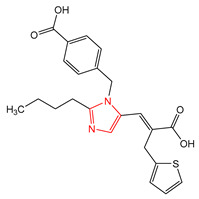	angiotensin receptor AT_1_ antagonist, lowering blood pressure
*Tiamazole*	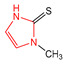	hyperthyroidism
*Dacarbazine*	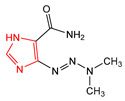	cytostatic
*Azathioprine*	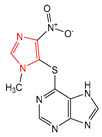	immuno-suppressive, cytotoxic

**Table 2:** The substituents of synthesized compounds and yields of reactions.

Compound	R^1^	Yield [%]	Compound	R^2^	Yield [%]
**2**	C_6_H_5_	87	**19**	C_6_H_5_	80
**3**	2-ClC_6_H_4_	86	**20**	2-ClC_6_H_4_	54
**4**	3-ClC_6_H_4_	86	**21**	3-ClC_6_H_4_	76
**5**	4-ClC_6_H_4_	81	**22**	2-FC_6_H_4_	46
**6**	2-FC_6_H_4_	87	**23**	2-OCH_3_C_6_H_4_	47
**7**	3-FC_6_H_4_	80	**24**	3-OCH_3_C_6_H_4_	65
**8**	4-FC_6_H_4_	83	**25**	4-OCH_3_C_6_H_4_	88
**9**	2-OCH_3_C_6_H_4_	90	**26**	4-CF_3_C_6_H_4_	38
**10**	3-OCH_3_C_6_H_4_	85	**27**	3-OHC_6_H_4_	65
**11**	4-OCH_3_C_6_H_4_	83	**28**	3-Cl-2-FC_6_H_3_	32
**12**	2-CF_3_C_6_H_4_	83	**29**	4-Cl-2-OCH_3_C_6_H_3_	60
**13**	3-CF_3_C_6_H_4_	89	**30**	2-OH-4-OCH_3_C_6_H_3_	48
**14**	4-CF_3_C_6_H_4_	85	**31**	2-OH-5-OCH_3_C_6_H_3_	67
**15**	2-CH_3_C_6_H_4_	82	**32**	4-OH-3-OCH_3_C_6_H_3_	76
**16**	3-CH_3_C_6_H_4_	85	**33**	4-OH-2-OCH_3_C_6_H_3_	58
**17**	4-CH_3_C_6_H_4_	83			
**18**	4-NO_2_C_6_H_4_	81			

**Table 3:** Antimicrobial activity of thiosemicarbazide derivatives in bacteria.

Compound	MIC [µg/mL] MBC [µg/mL] MBC/MIC Ratio						
	Gram-Positive Bacteria						
	Sa	Sa	Sa	Se	Ml	Bs	Bc
	25923	6538	43300	12228	10240	6633	10876
**2**	500	1000	>1000	1000	1000	1000	>1000
	>1000	>1000	n.d	1000	1000	>1000	n.d.
	-	-	-	1	1	-	-
**3**	250	500	1000	250	250	125	1000
	>1000	>1000	>1000	500	250	125	>1000
	-	-	-	2	1	1	-
**4**	62.5	250	500	125	125	62.5	250
	>1000	>1000	>1000	125	500	62.5	>1000
	-	-	-	1	4	1	-
**5**	62.5	125	125	125	125	62.5	250
	>1000	>1000	>1000	125	125	62.5	250
	-	-	-	1	1	1	1
**6**	250	1000	1000	500	250	250	500
	>1000	>1000	>1000	500	250	500	1000
	-	-	-	1	1	2	2
**7**	250	500	500	250	250	125	500
	>1000	>1000	>1000	>1000	250	250	500
	-	-	-	-	1	2	1
**8**	250	>1000	1000	500	250	250	500
	>1000	n.d.	>1000	500	500	250	1000
	-	-	-	1	2	1	2
**9**	500	1000	1000	500	500	500	1000
	>1000	>1000	>1000	>1000	>1000	500	>1000
	-	-	-	-	-	1	-
**10**	250	1000	500	500	250	125	500
	>1000	>1000	>1000	500	250	500	1000
	-	-	-	1	1	4	2
**11**	500	1000	1000	500	250	250	250
	>1000	>1000	>1000	>1000	500	250	250
	-	-	-	-	2	1	1
**12**	250	500	1000	500	250	250	500
	>1000	>1000	>1000	500	500	250	500
	-	-	-	1	2	1	1
**13**	500	>1000	>1000	1000	500	500	1000
	>1000	n.d.	n.d.	>1000	>1000	500	>1000
	-	-	-	-	-	1	-
**14**	31.25	62.5	62.5	31.25	62.5	31.25	31.25
	>1000	1000	500	500	500	31.25	250
	-	16	8	16	8	1	8
**15**	500	250	250	500	500	1000	500
	>1000	>1000	>1000	500	500	1000	500
	-	-	-	1	1	1	1
**16**	250	62.5	125	500	500	500	250
	>1000	>1000	>1000	500	>1000	500	>1000
	-	-	-	1	-	1	-
**17**	125	125	125	500	500	500	62.5
	>1000	125	>1000	>1000	>1000	>1000	>1000
	-	1	-	-	-	-	-
**18**	500	>1000	>1000	1000	500	1000	1000
	>1000	n.d.	n.d.	>1000	1000	1000	1000
	-	-	-	-	2	1	1
**AMX**	0.98	0.98	31.25	0.98	0.98	125	125
	0.98	0.98	31.25	0.98	0.98	125	125

**Abbreviations:** Sa 25923—*Staphylococcus aureus* ATCC 25923, Sa 6538—*Staphylococcus aureus* ATCC 6538, Sa 43300—*Staphylococcus aureus* ATCC 43300 methicillin-resistant MRSA, Se 12228—*Staphylococcus epidermidis* ATCC 12228, Ml 10240—*Micrococcus luteus* ATCC 10240, Bs 6633—*Bacillus subtilis* ATCC 6633, Bc 10876—*Bacillus cereus* ATCC 10876, MIC—the minimal inhibitory concentration [µg/mL], MBC—the minimal bactericidal concentration [µg/mL], AMX—amoxicillin [500 µg/mL], colored position—MBC(MFC)/MIC < 4 determined as bactericidal/fungicidal effect of tested compounds, n.d.—not defined.

**Table 4:** Antimicrobial activity of thiosemicarbazide derivatives in fungi.

Compound	MIC [µg/mL] MFC [µg/mL] MFC/MIC Ratio						
	Yeasts						
	Cp	Cg	Ck	Cg	Ct	Ca	Cl
	22019	90030	14243	15126	1369	B11903	3449
**2**	500	>1000	>1000	>1000	500	>1000	>1000
	>1000	n.d.	n.d.	n.d.	>1000	n.d.	n.d.
	-	-	-	-	-	-	-
**3**	250	>1000	>1000	>1000	250	>1000	>1000
	>1000	n.d.	n.d.	n.d.	500	n.d.	n.d.
	-	-	-	-	2	-	-
**4**	62.5	>1000	>1000	>1000	250	>1000	>1000
	>1000	n.d.	n.d.	n.d.	500	n.d.	n.d.
	-	-	-	-	2	-	-
**5**	62.5	>1000	>1000	>1000	500	>1000	>1000
	>1000	n.d.	n.d.	n.d.	>1000	n.d.	n.d.
	-	-	-	-	-	-	-
**6**	1000	>1000	>1000	>1000	250	>1000	>1000
	>1000	n.d.	n.d.	n.d.	>1000	n.d.	n.d.
	-	-	-	-	-	-	-
**7**	>1000	>1000	>1000	>1000	250	>1000	>1000
	n.d.	n.d.	n.d.	n.d.	>1000	n.d.	n.d.
	-	-	-	-	-	-	-
**8**	1000	>1000	>1000	>1000	250	>1000	>1000
	>1000	n.d.	n.d.	n.d.	>1000	n.d.	n.d.
	-	-	-	-	-	-	-
**9**	500	>1000	>1000	>1000	500	>1000	>1000
	1000	n.d.	n.d.	n.d.	1000	n.d.	n.d.
	2	-	-	-	2	-	-
**10**	250	>1000	>1000	>1000	250	>1000	>1000
	>1000	n.d.	n.d.	n.d.	1000	n.d.	n.d.
	-	-	-	-	4	-	-
**11**	250	>1000	>1000	>1000	250	>1000	>1000
	>1000	n.d.	n.d.	n.d.	>1000	n.d.	n.d.
	-	-	-	-	-	-	-
**12**	125	>1000	>1000	>1000	500	>1000	>1000
	>1000	n.d.	n.d.	n.d.	1000	n.d.	n.d.
	-	-	-	-	2	-	-
**13**	500	>1000	>1000	>1000	>1000	>1000	>1000
	1000	n.d.	n.d.	n.d.	n.d.	n.d.	n.d.
	2	-	-	-	-	-	-
**14**	>1000	>1000	>1000	>1000	500	>1000	>1000
	n.d.	n.d.	n.d.	n.d.	500	n.d.	n.d.
	-	-	-	-	1	-	-
**15**	>1000	>1000	>1000	>1000	500	>1000	>1000
	n.d.	n.d.	n.d.	n.d.	>1000	n.d.	n.d.
	-	-	-	-	-	-	-
**16**	500	>1000	>1000	>1000	>1000	>1000	>1000
	>1000	n.d.	n.d.	n.d.	n.d.	n.d.	n.d.
	-	-	-	-	-	-	-
**17**	>1000	>1000	>1000	>1000	250	>1000	>1000
	n.d.	n.d.	n.d.	n.d.	>1000	n.d.	n.d.
	-	-	-	-	-	-	-
**18**	>1000	>1000	>1000	>1000	500	>1000	>1000
	n.d.	n.d.	n.d.	n.d.	>1000	n.d.	n.d.
	-	-	-	-	-	-	-
**NYS**	12.5	1.56	12.5	1.56	<0.781	6.25	1.56
	12.5	3.125	12.5	3.125	0.5	15.625	3.125

**Abbreviations:** Cp 22019—*Candida parapsilosis* ATCC 22019, Cg 90030—*Candida glabrata* ATCC 90030, Ck 14243—*Candida krusei* ATCC 14243, Cg 15126—*Candida glabrata* ATCC 15126, Ct 1369—*Candida tropicalis* ATCC 1369, Ca B11903—*Candida auris* CDC B11903, Cl 3449—*Candida lusitaniae* ATCC 3449, MIC—the minimal inhibitory concentration [µg/mL], MFC—the minimal fungicidal concentration [µg/mL], NYS—nystatin [500 µg/mL], colored position—MFC/MIC < 4 determined as bactericidal/fungicidal effect of tested compounds, n.d.—not defined.

**Table 5:** Cytotoxic activity (IC_50_, µM) of studied compounds estimated by the MTT assay *^a^*.

Compound	Cancer Cells				Normal Cells				
	SW620 *^d^*		MDA-MB-231 *^e^*		A-549 *^f^*		PC3 *^g^*		HaCaT *^h^*
	IC_50_ *^b^*	SI *^c^*	IC_50_	SI	IC_50_	SI	IC_50_	SI	IC_50_
**30**	64.2 ± 6.88	1.5	>100	0.9	>100	0.9	70.2 ± 4.20	1.4	97.0 ± 5.48
**31**	86.3 ± 3.81	1.1	>100	1	>100	1	70.4 ± 2.76	1.4	>100
Doxo-rubicin *^i^*	0.3 ± 0.1	1.0	1.6 ± 0.23	0.19	0.2 ± 0.09	1.5	0.3 ± 0.1	1	0.3 ± 0.1
Cisplatin *^j^*	6.7 ± 1.1	0.9	7.8 ± 0.98	0.81	3.2 ± 1.24	5.08	13.2 ± 2.1	0.5	6.3 ± 0.7

*^a^* Data are expressed as mean SD, *^b^* IC_50_ (µM)—the half-maximal growth inhibitory concentration after culturing the cells for 24 h with the individual compound. *^c^* The SI (Selectivity Index) was calculated using the formula: SI = IC_50_ for normal cell line/IC_50_ cancer cell line. *^d^* Human metastatic colon cancer (SW620), *^e^* Human breast adenocarcinoma (MDA-MB-231), *^f^* Human lung carcinoma (A-549), *^g^* Human metastatic prostate cancer (PC3), *^h^* Human immortal keratinocyte cell line from adult human skin (HaCaT). *^i, j^* The reference compounds.

## Data Availability

Data are contained within the article and Supplementary Materials.

## Supplementary Materials

The following supporting information can be downloaded at: https://www.mdpi.com/article/10.3390/molecules29133023/s1, ^1^H NMR, ^13^C NMR and MS spectra for the compounds.
