# Supplementary material for: Synthesis and Biological Evaluation of New Compounds with Nitroimidazole Moiety
Source: Molecules. 2024 Jun 26;29(13):3023. doi: 10.3390/molecules29133023 (PMC11243693; doi:10.3390/molecules29133023)
Supplement: Supplementary file 1 [file molecules-29-03023-s001.zip › molecules-3057680-SI.pdf]

Supplementary material

Content

1. ^{1}H NMR spectra for the compounds number 14 - 33……………………….2
2. ^{13}C NMR spectra for the compounds number 14 - 33………………...……22
3. MS spectra for the compounds number 14 - 33………………...…………..42
4. ^{13}C NMR spectra for the compounds number 2 - 13…………………...…..62

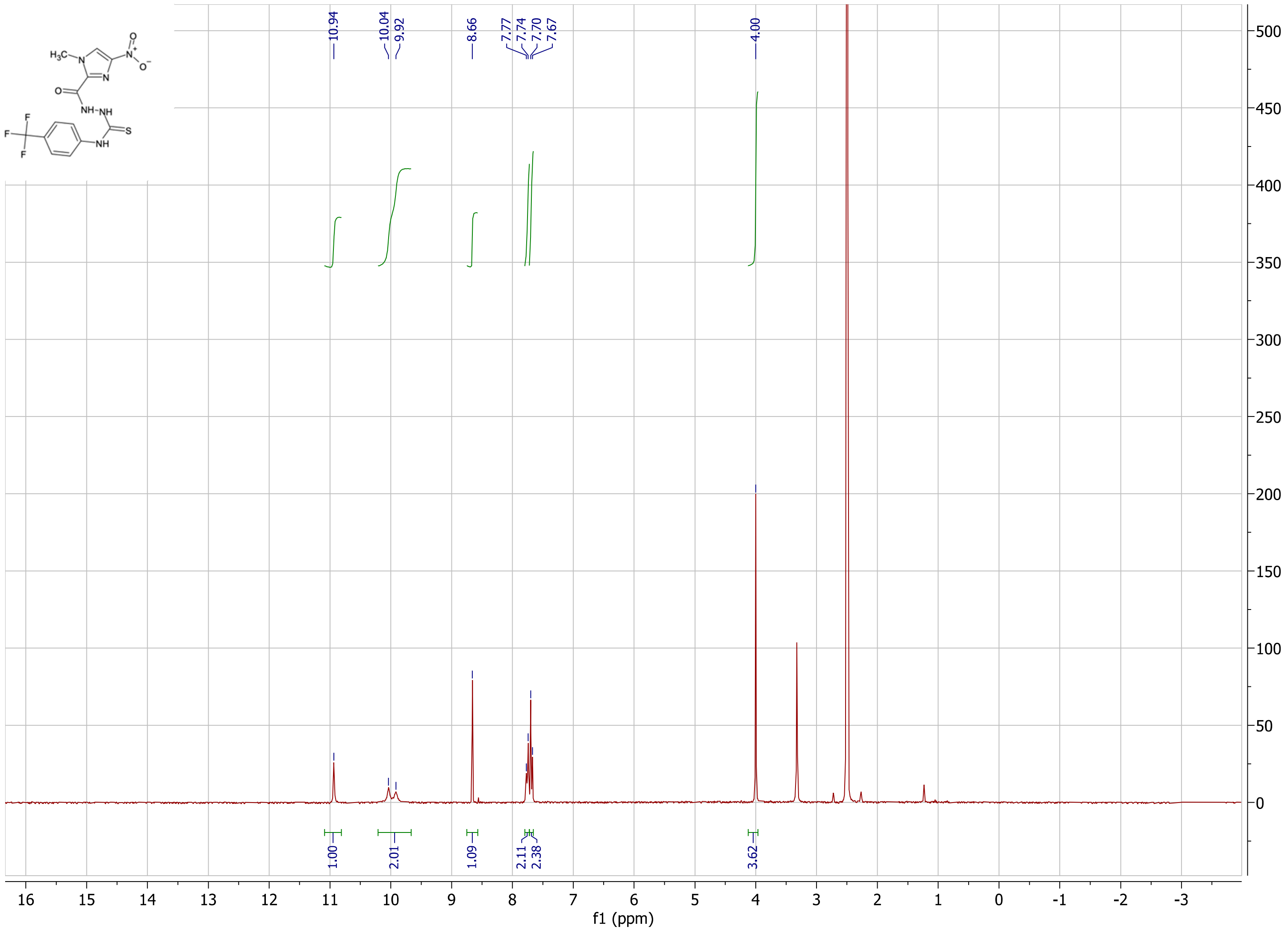

Figure S1. The ^{1}H NMR of compound **14**.

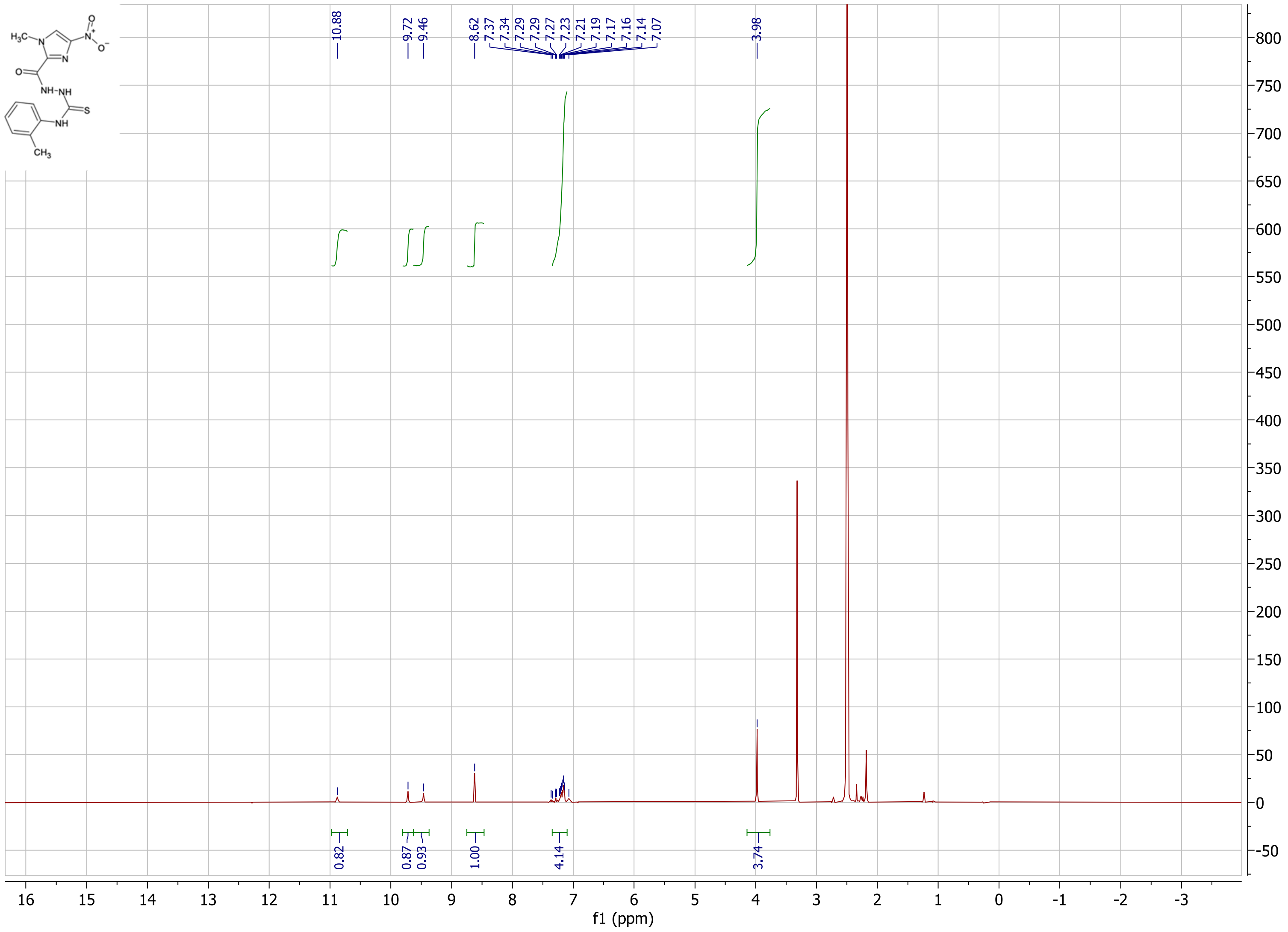

Figure S2. The ^{1}H NMR of compound **15**.

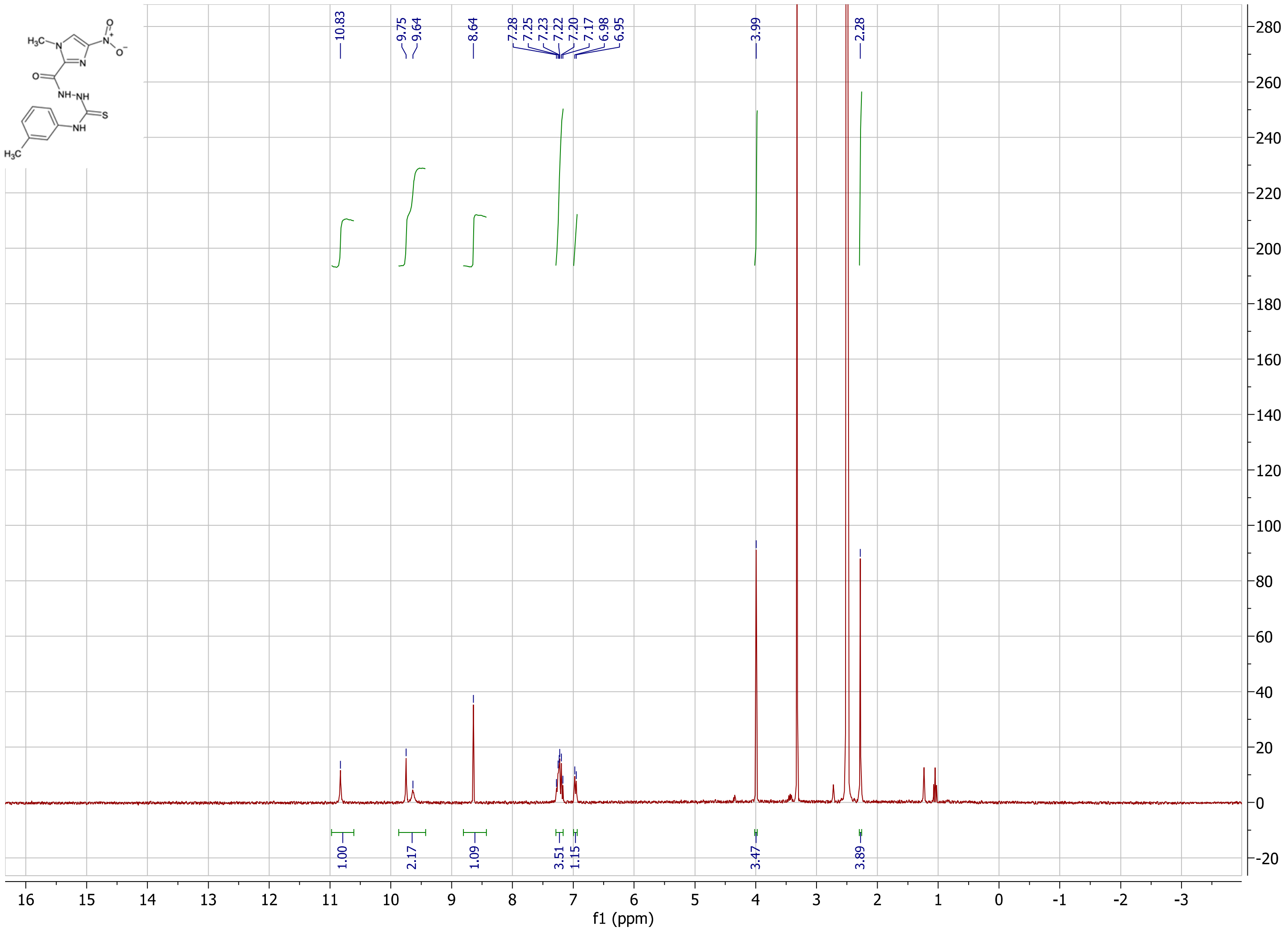

Figure S3. The ^{1}H NMR of compound **16**.

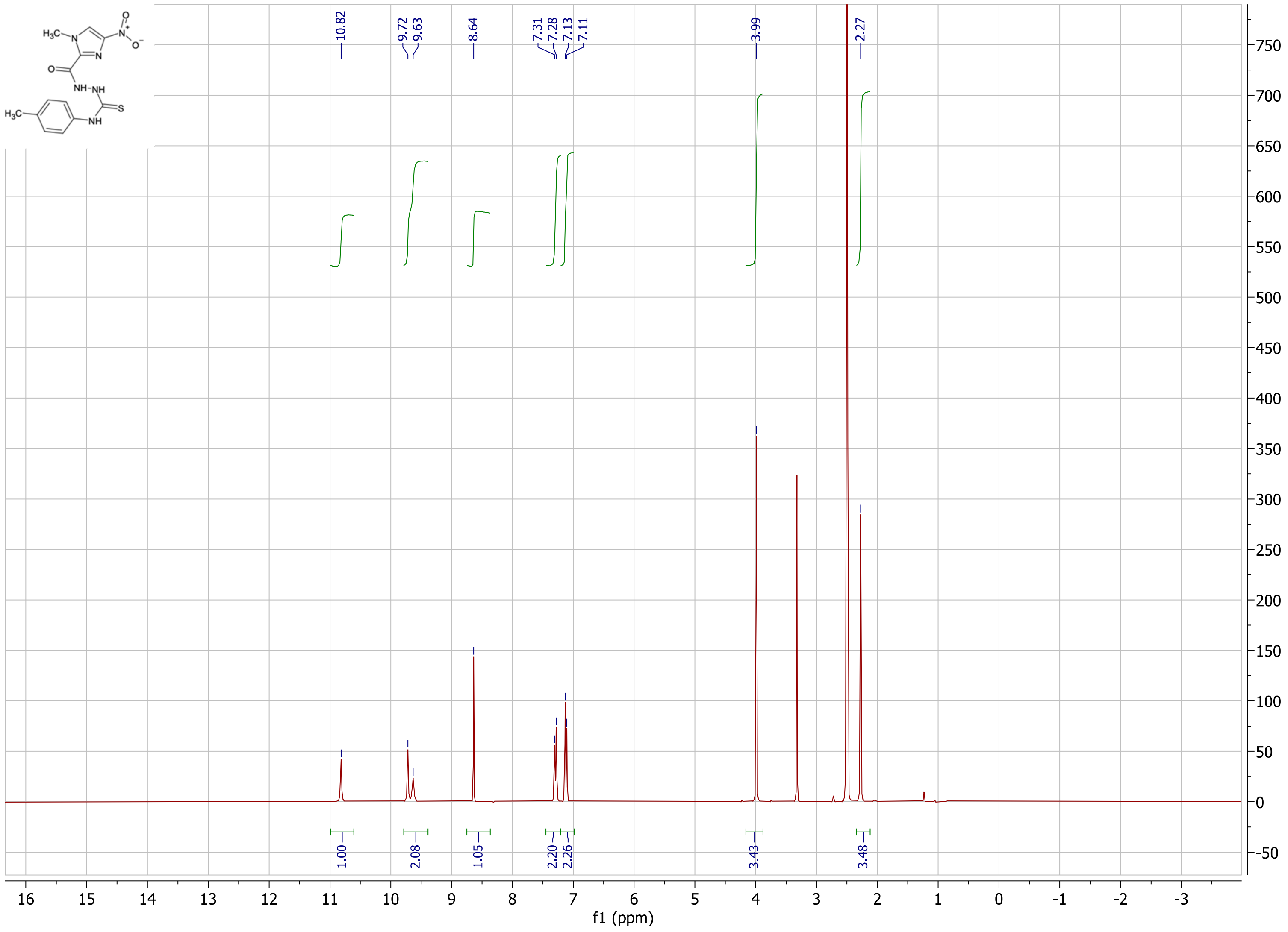

Figure S4. The ^{1}H NMR of compound **17**.

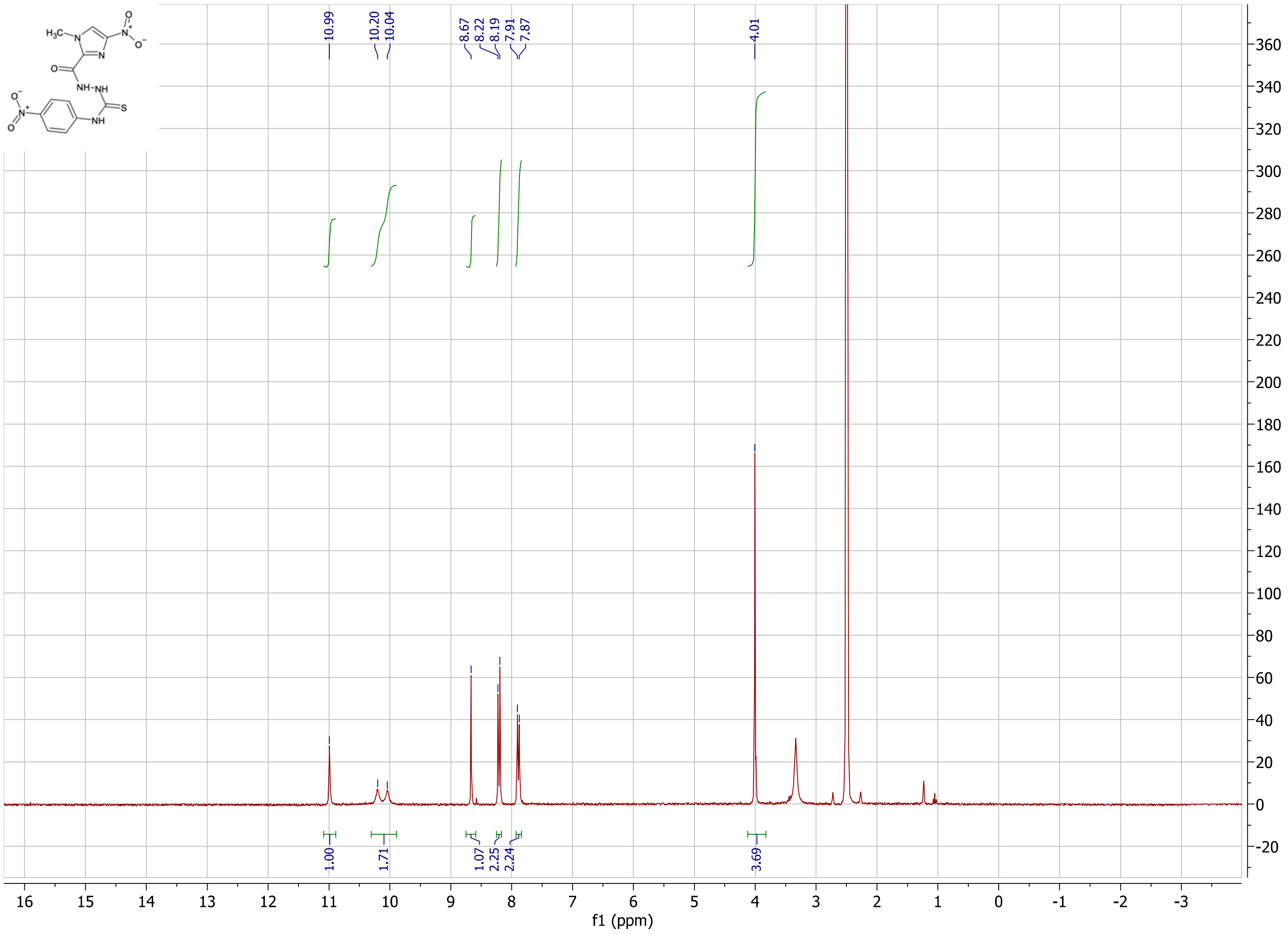

Figure S5. The ^{1}H NMR of compound **18**.

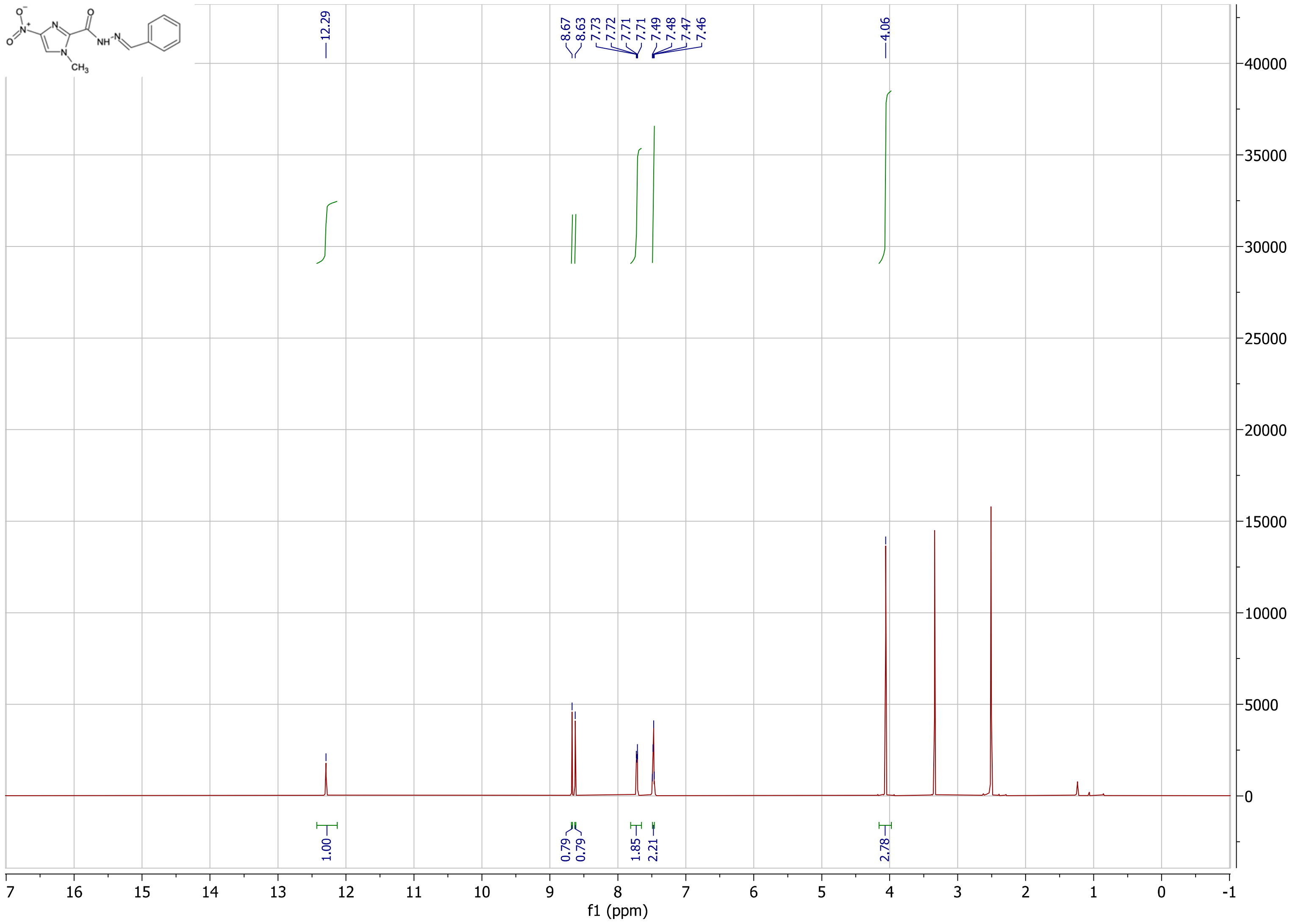

Figure S6. The ^{1}H NMR of compound **19**.

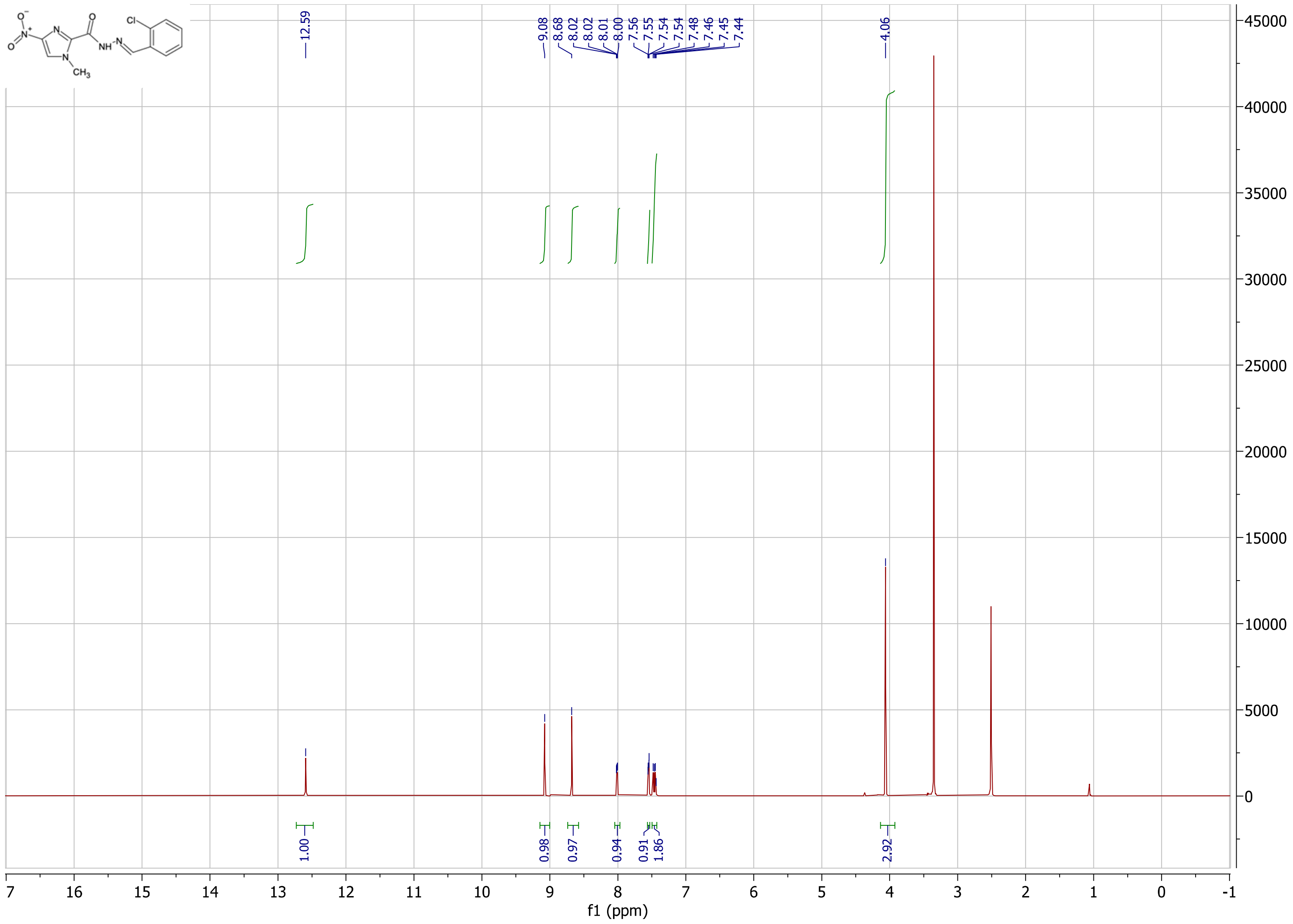

Figure S7. The ^{1}H NMR of compound **20**.

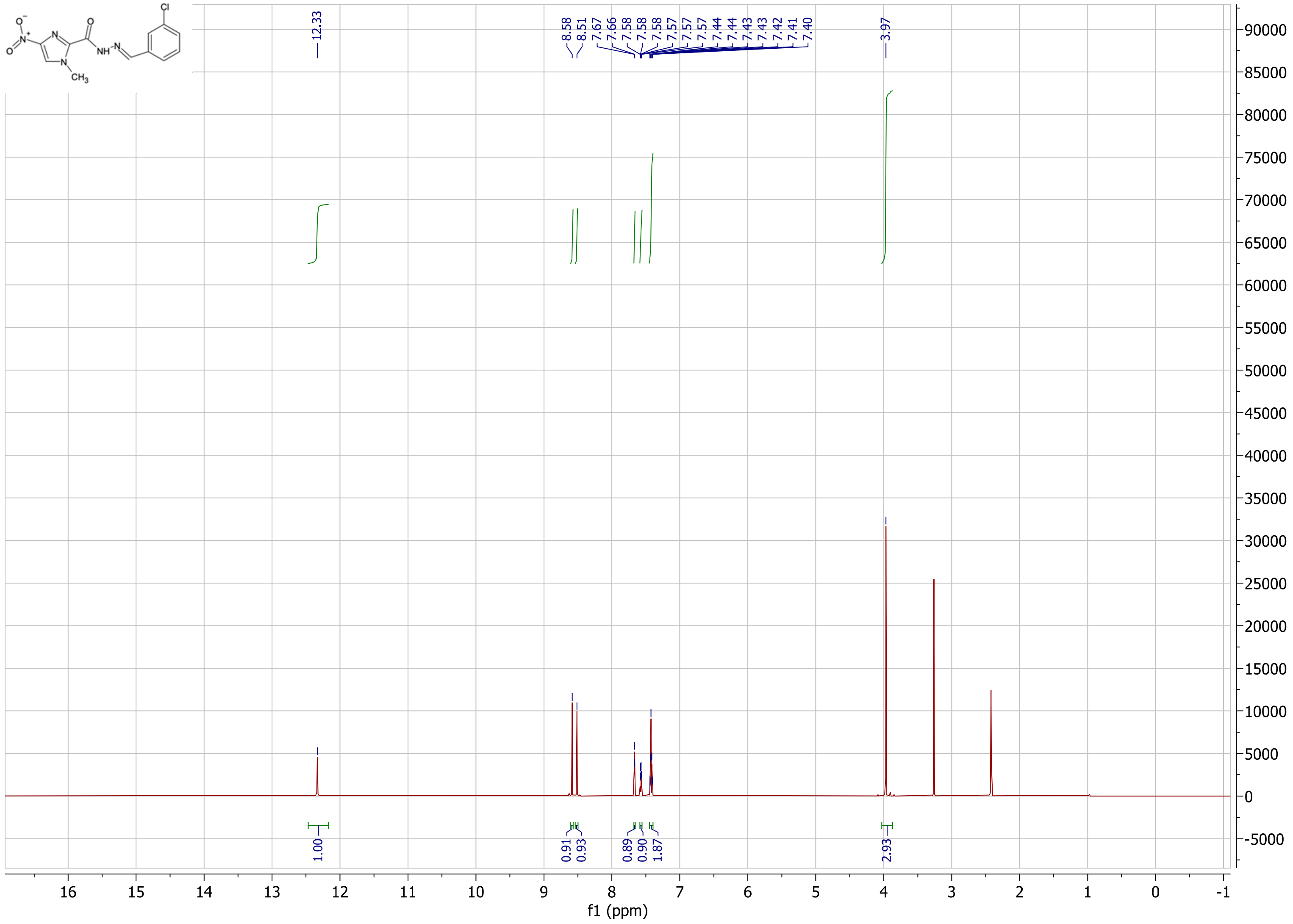

Figure S8. The ¹H NMR of compound **21**.

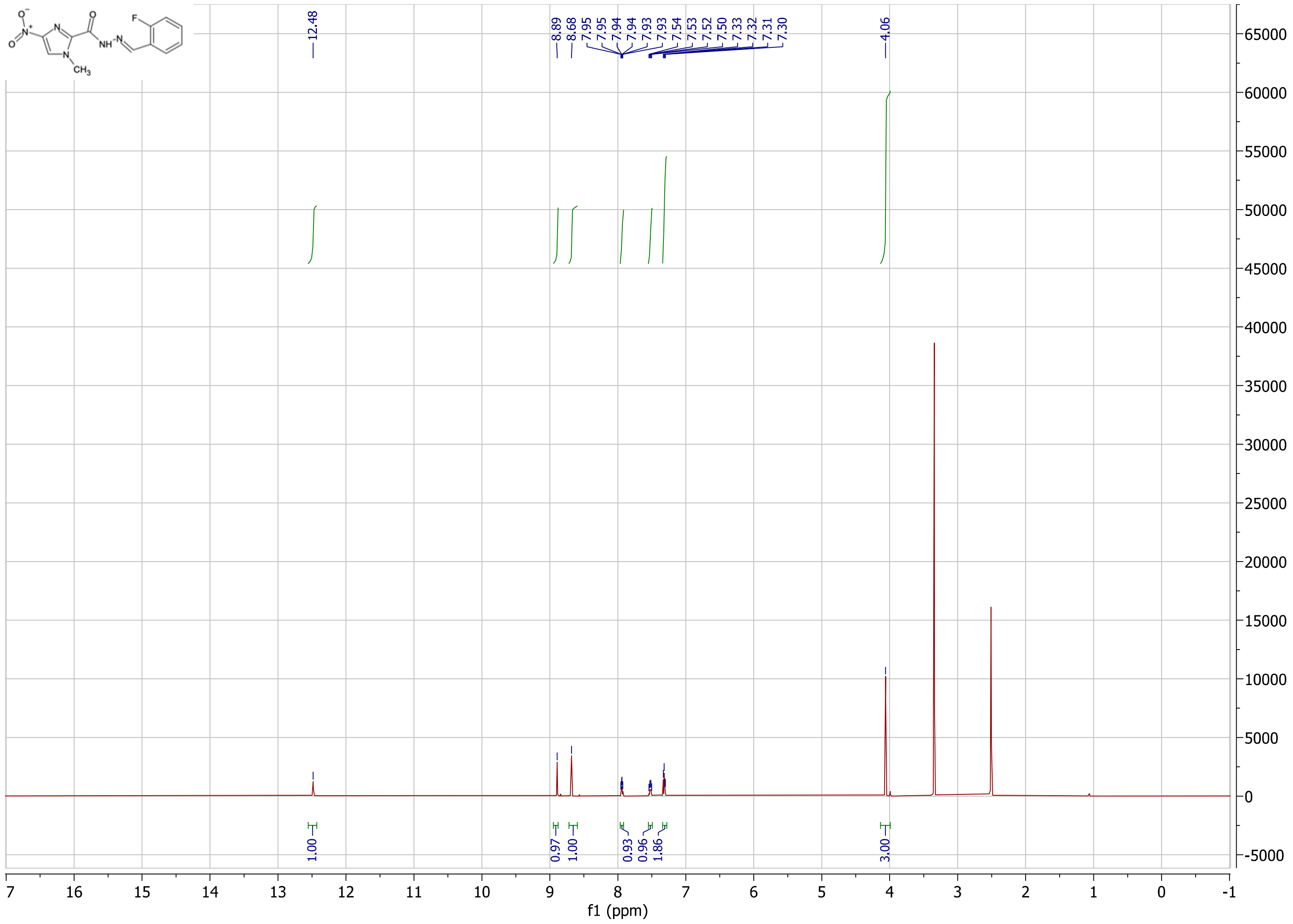

Figure S9. The ^{1}H NMR of compound **22**.

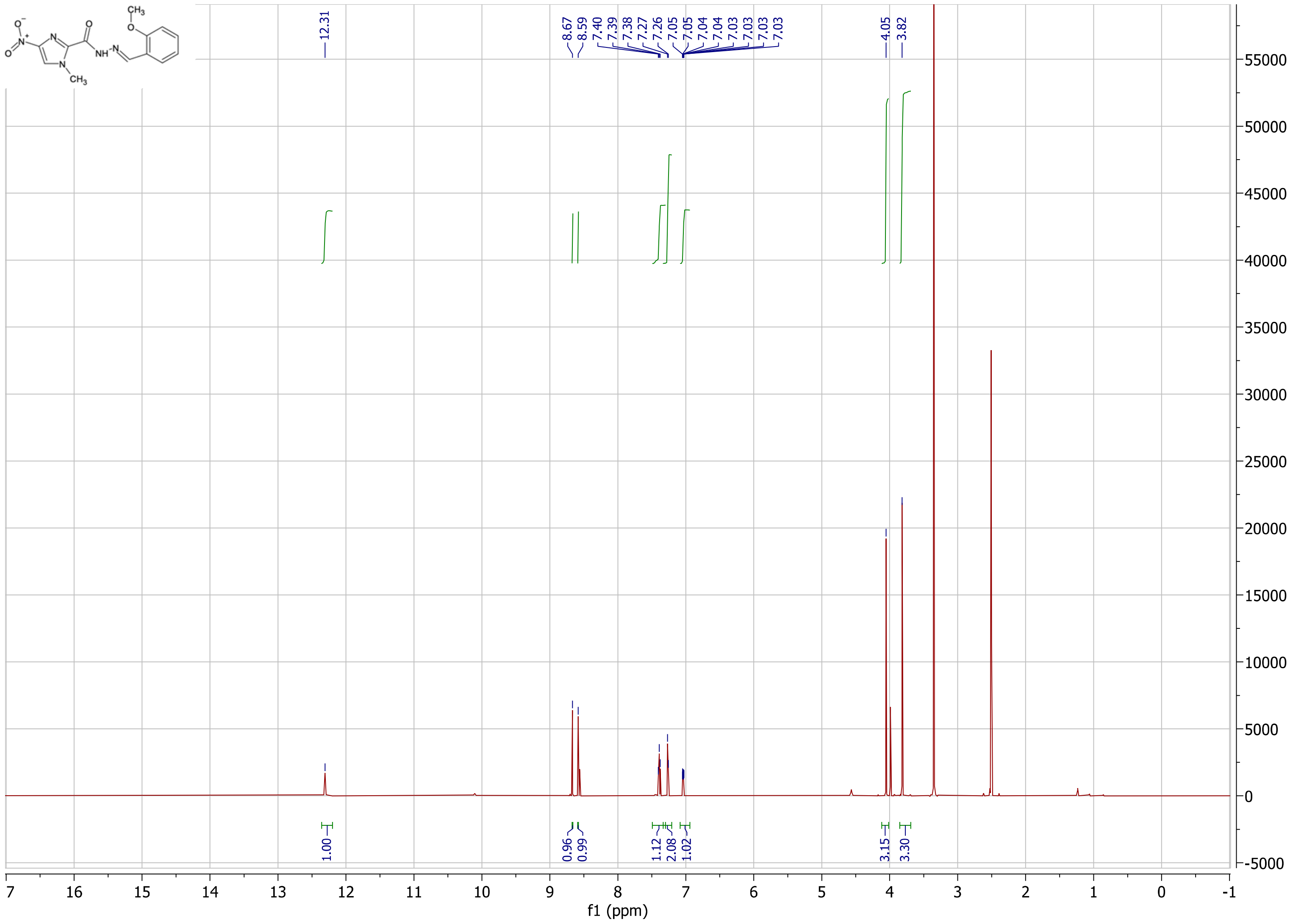

Figure S10. The ¹H NMR of compound **23**.

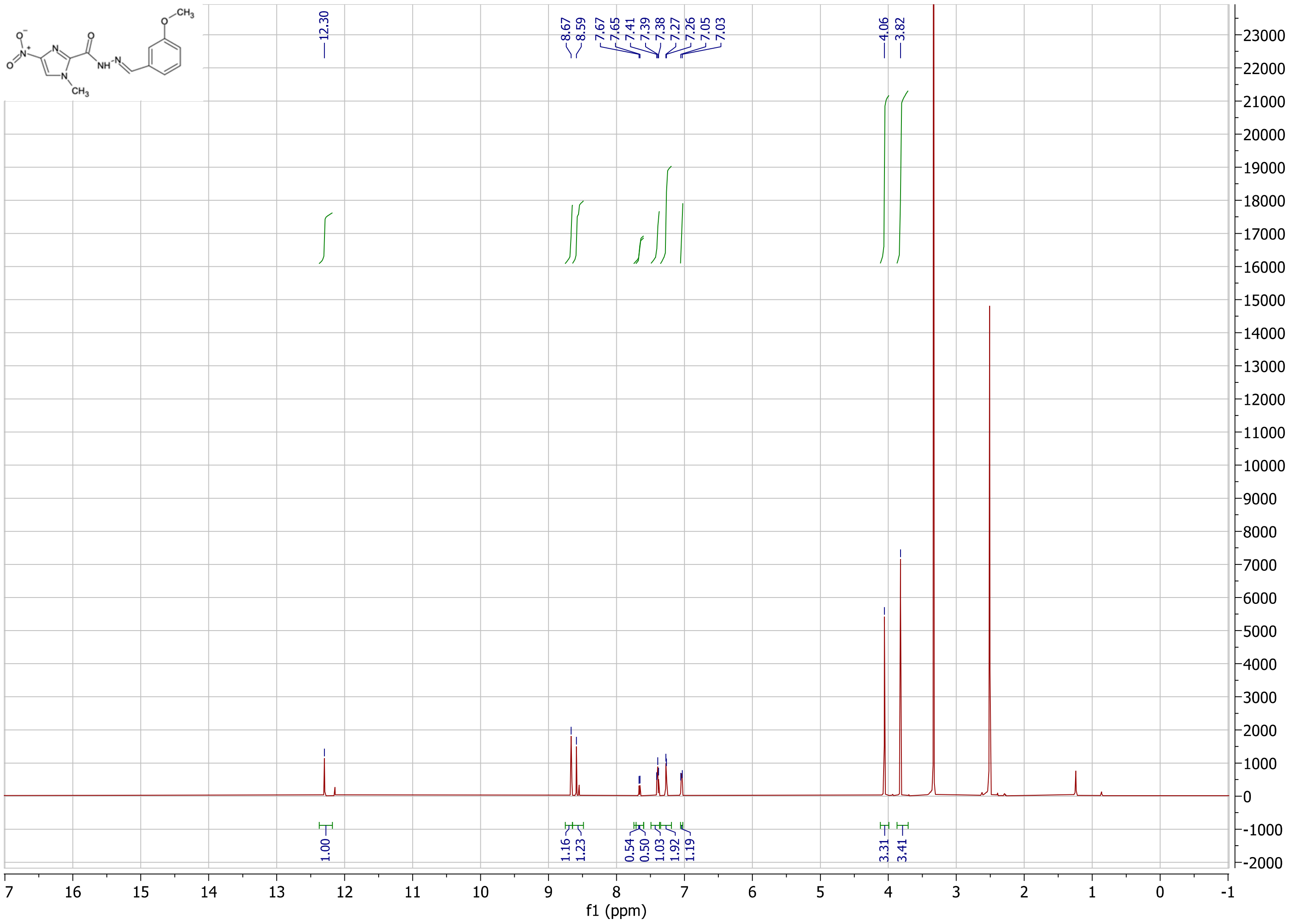

Figure S11. The ^{1}H NMR of compound **24**.

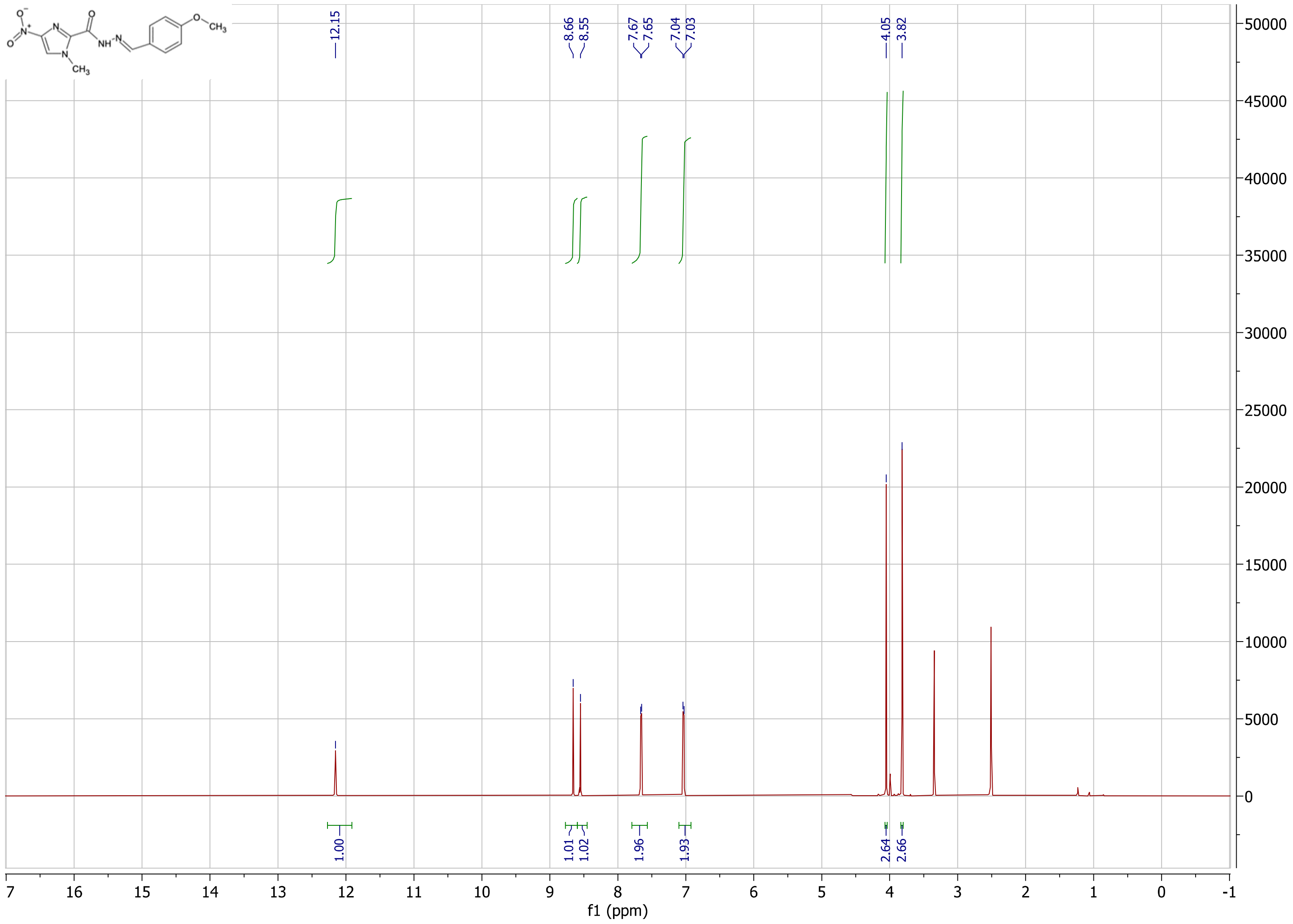

Figure S12. The ^{1}H NMR of compound **25**.

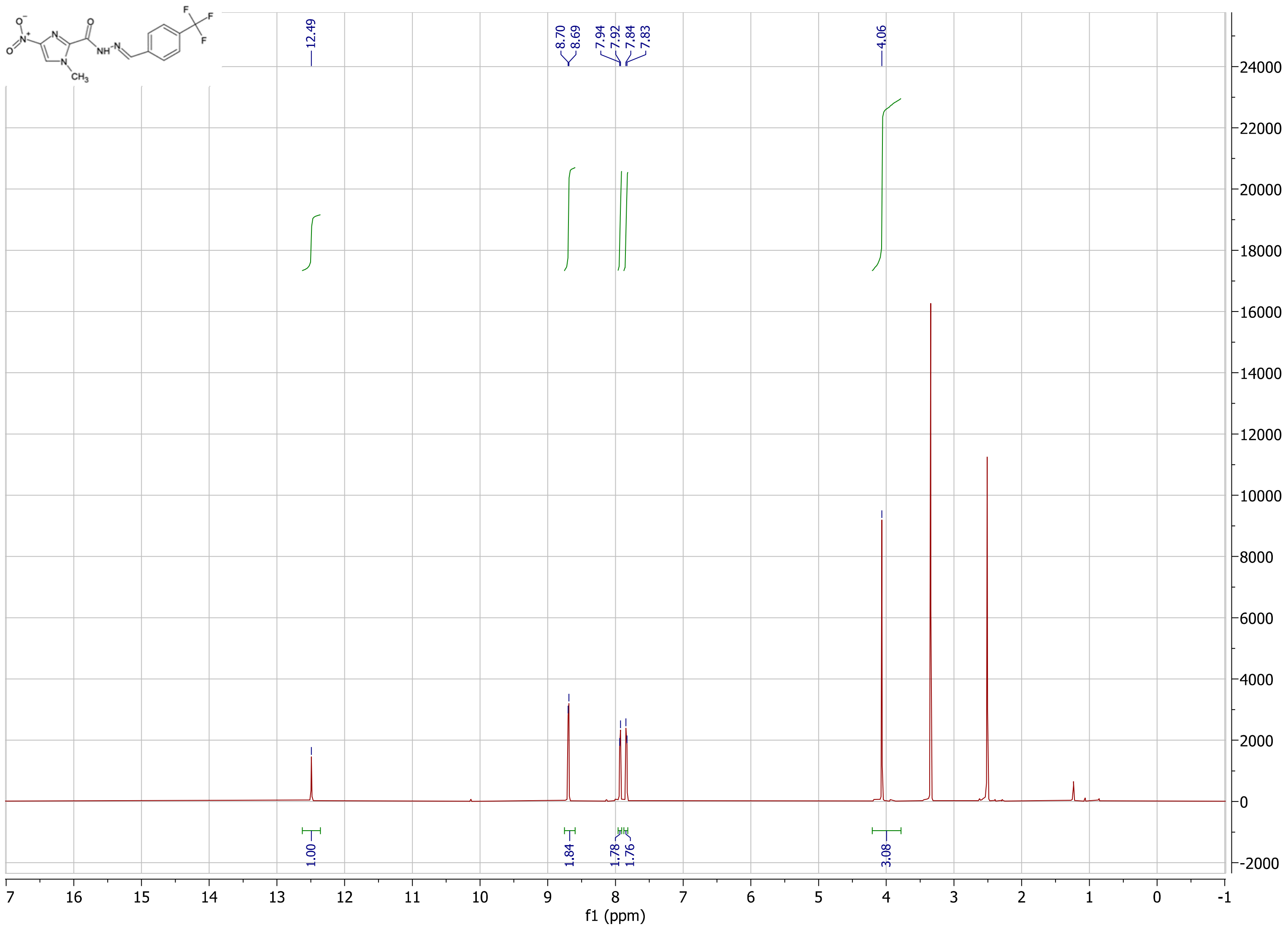

Figure S13. The ^{1}H NMR of compound **26**.

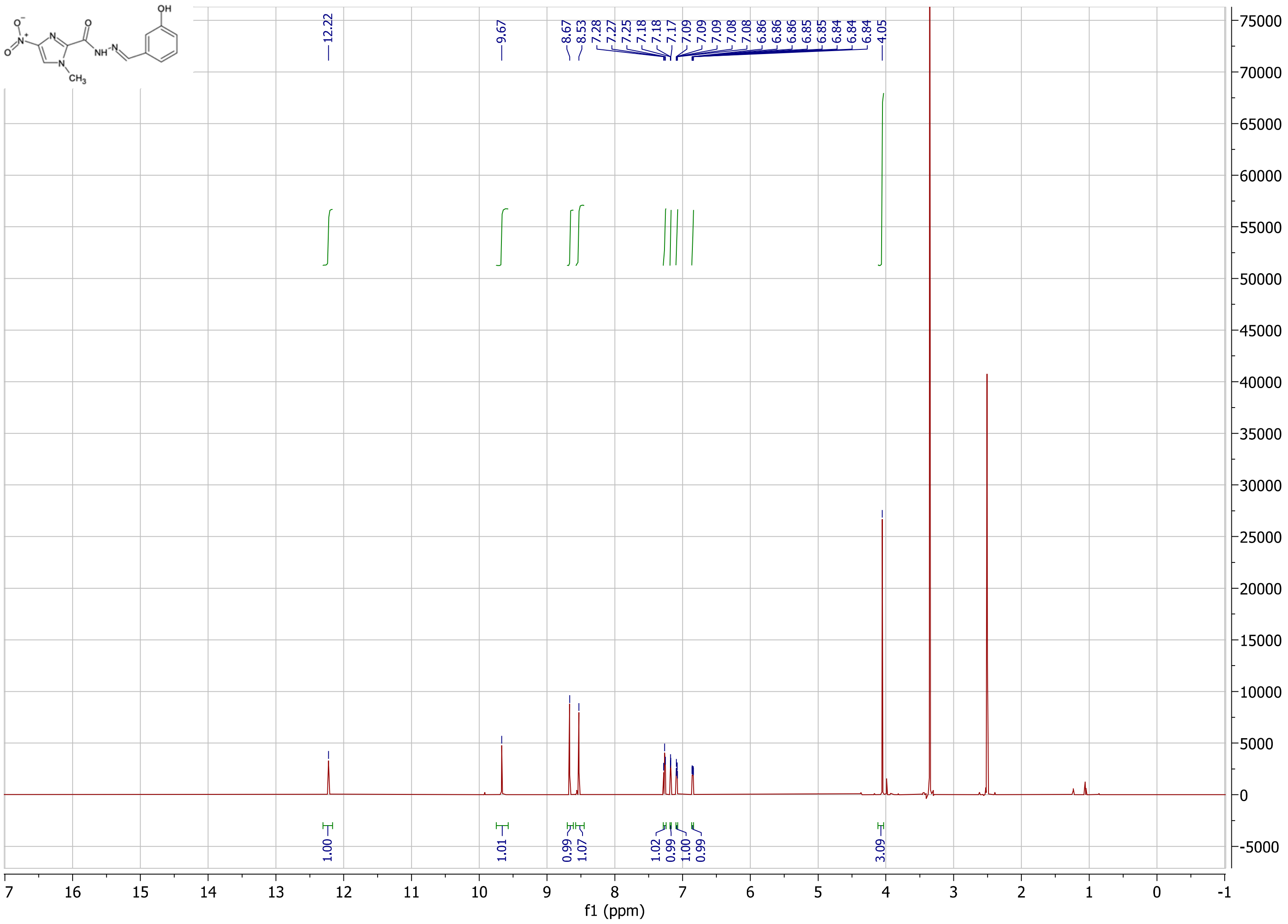

Figure S14. The ^{1}H NMR of compound **27**.

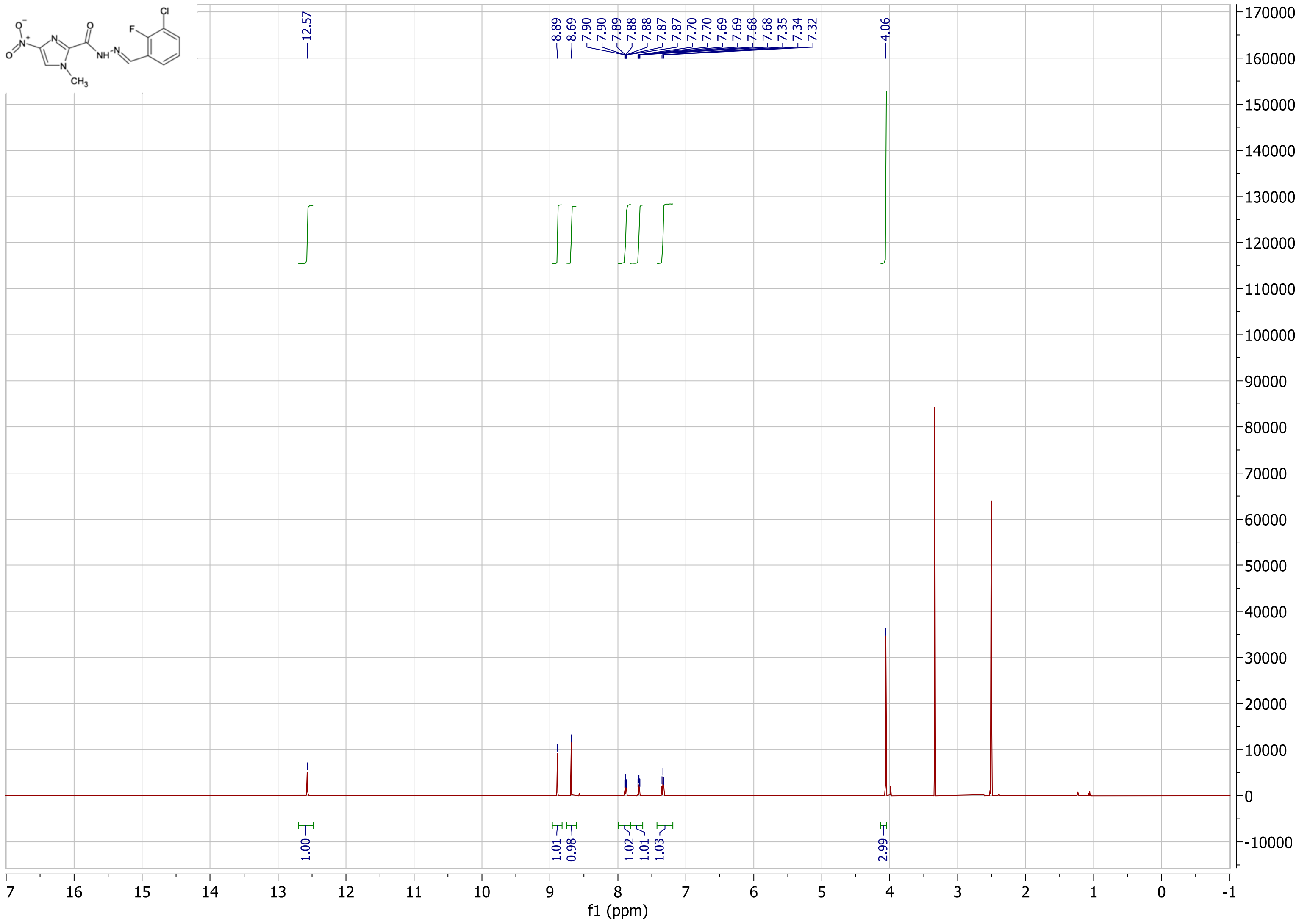

Figure S15. The ^{1}H NMR of compound **28**.

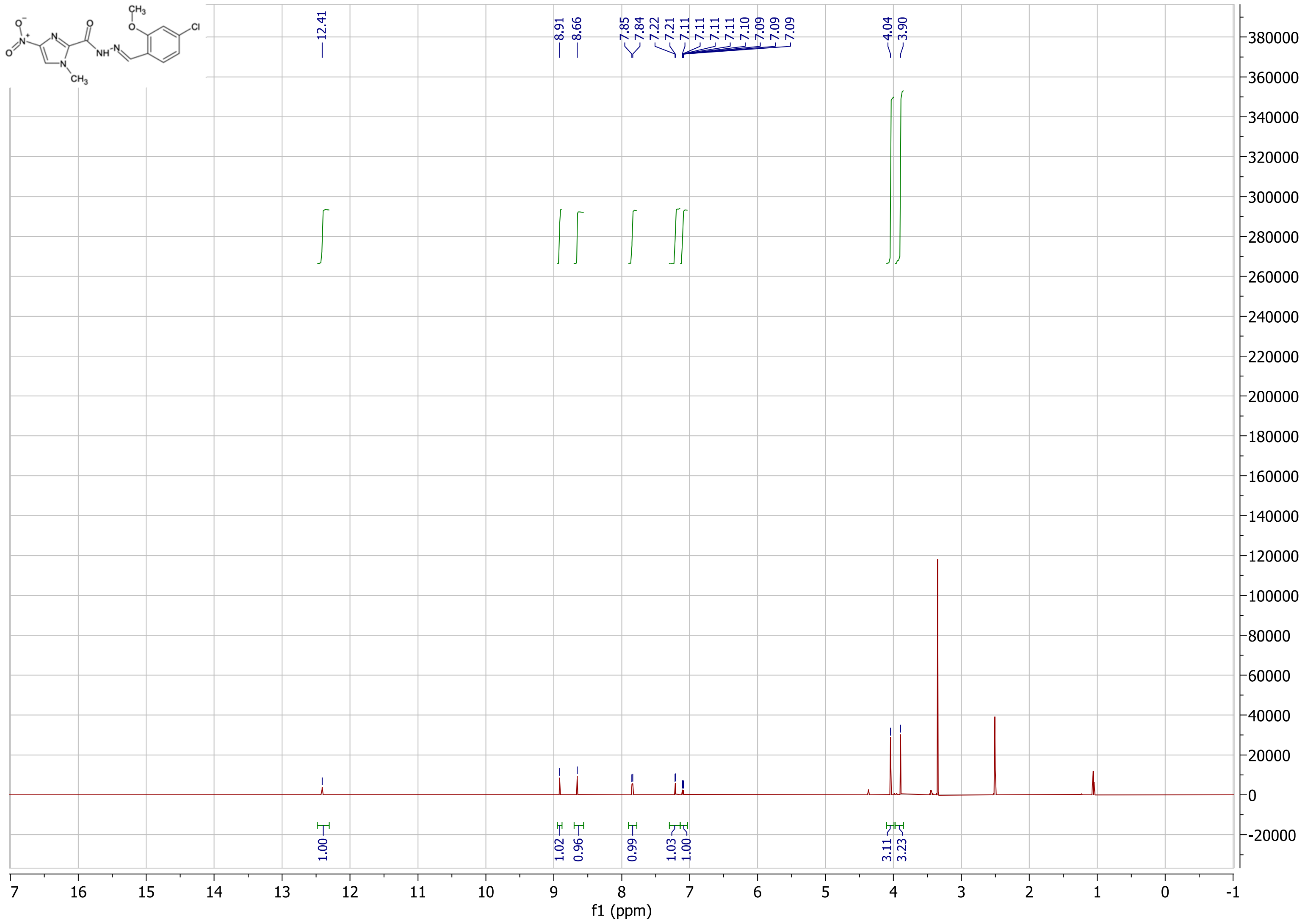

Figure S16. The ^{1}H NMR of compound **29**.

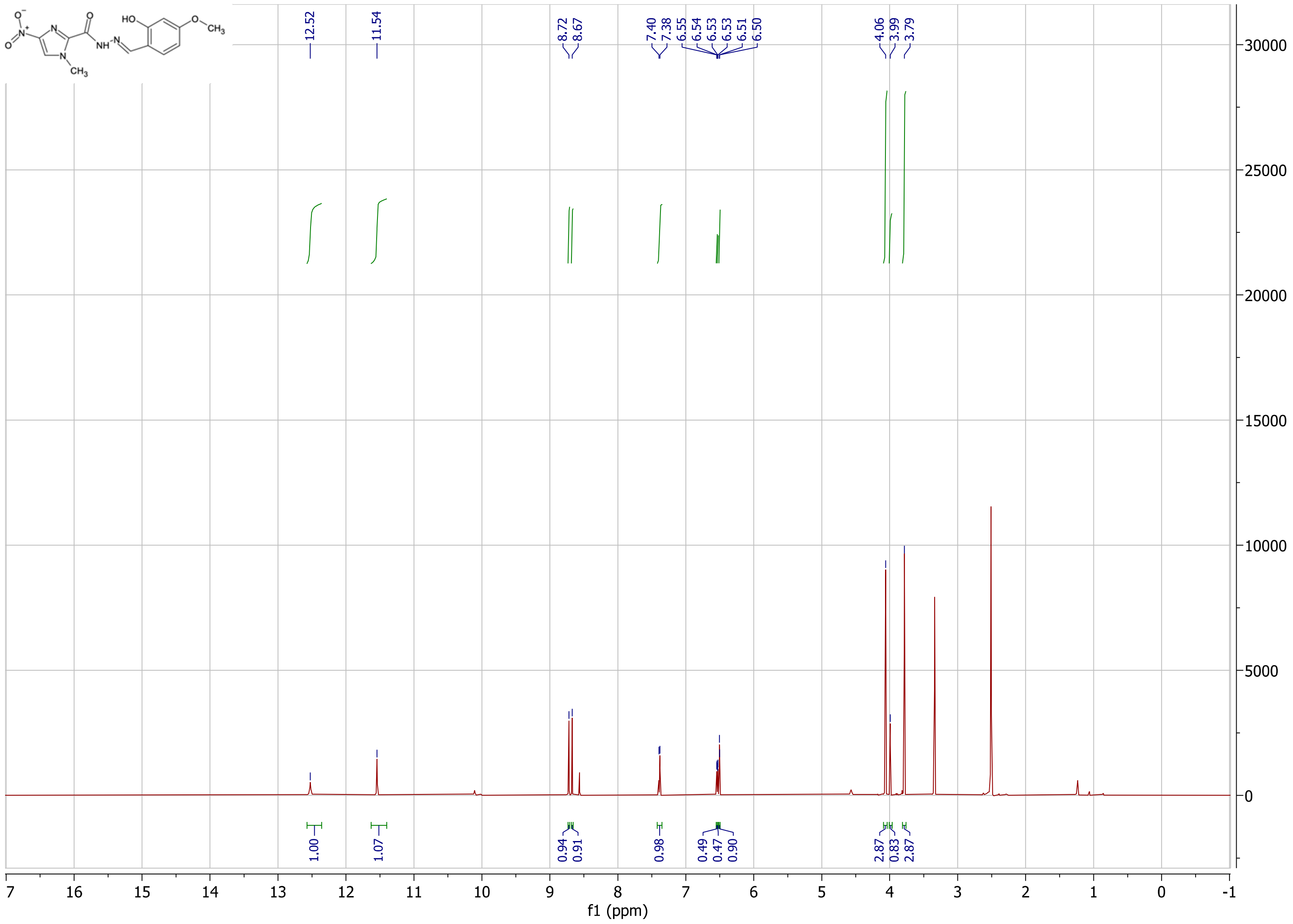

Figure S17. The ^{1}H NMR of compound **30**.

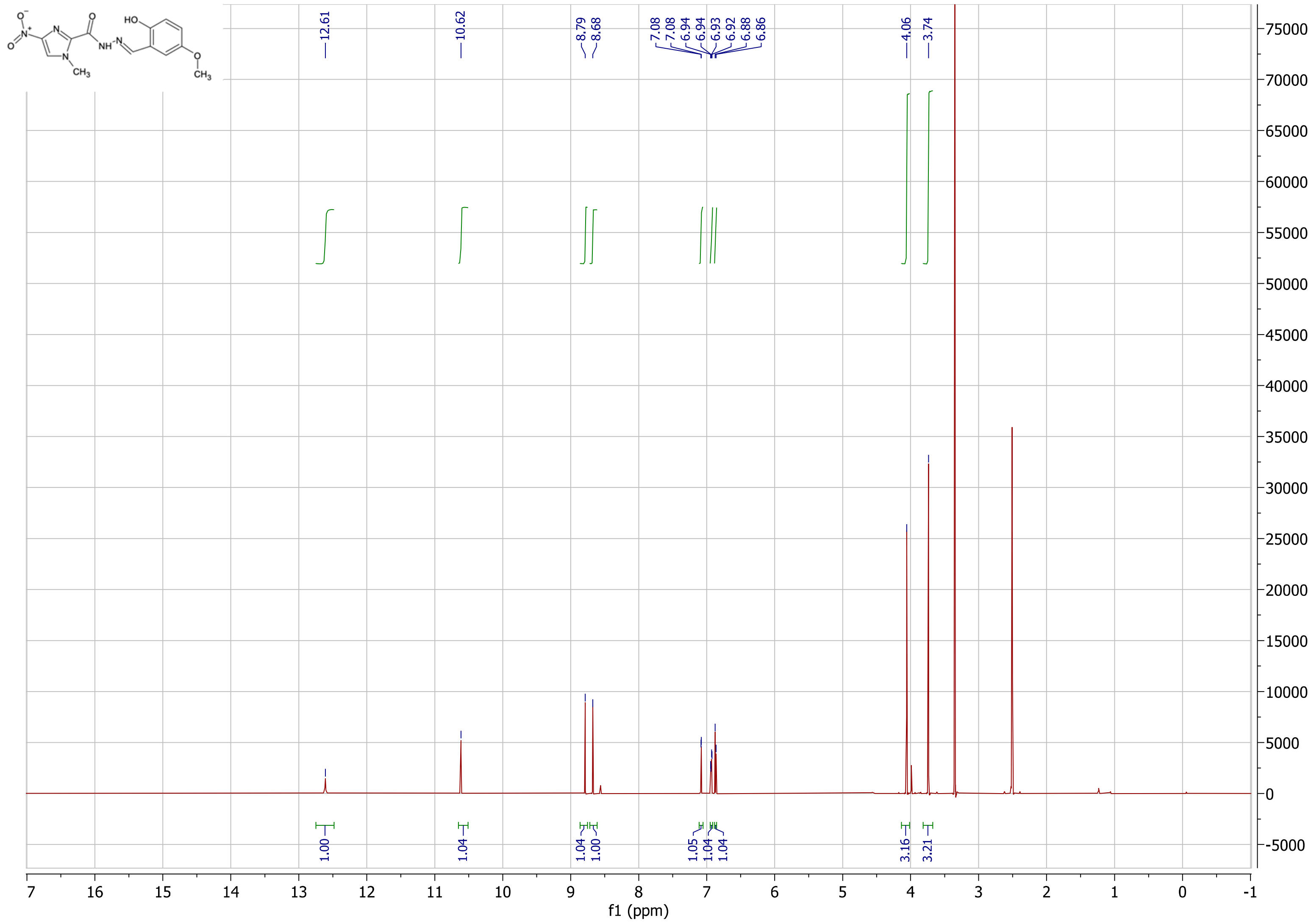

Figure S18. The ^{1}H NMR of compound **31**.

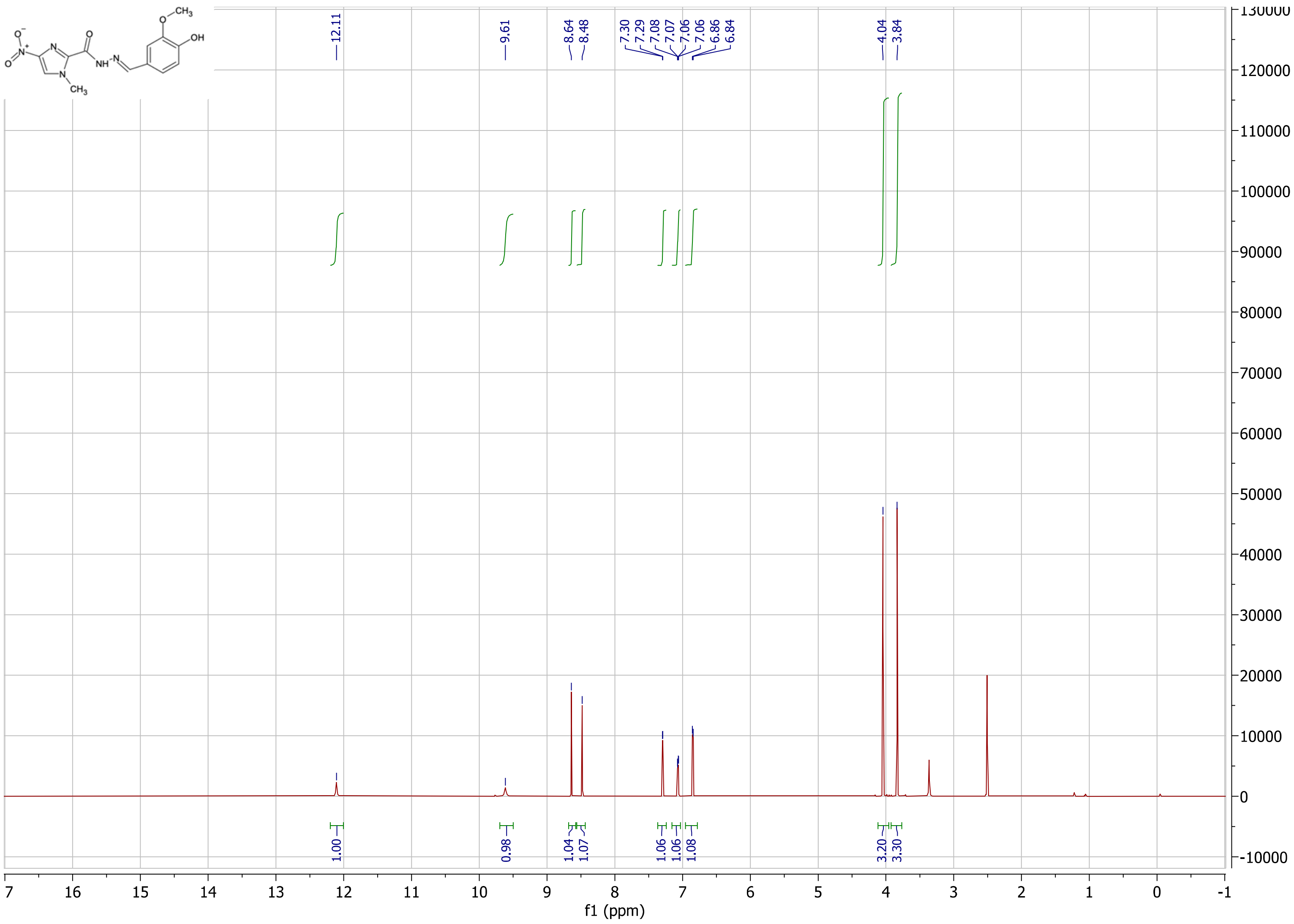

Figure S19. The ^{1}H NMR of compound **32**.

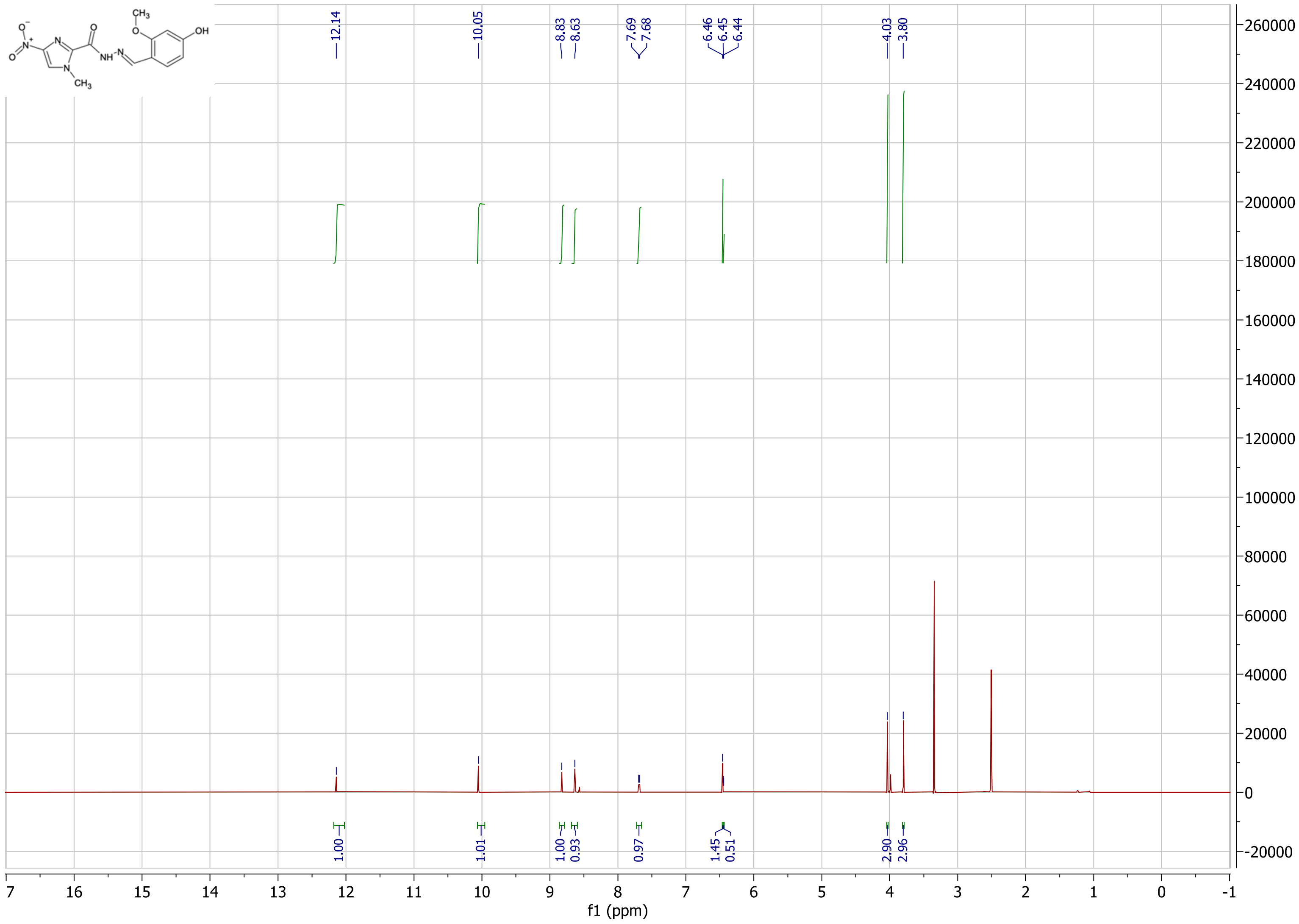

Figure S20. The ^{1}H NMR of compound **33**.

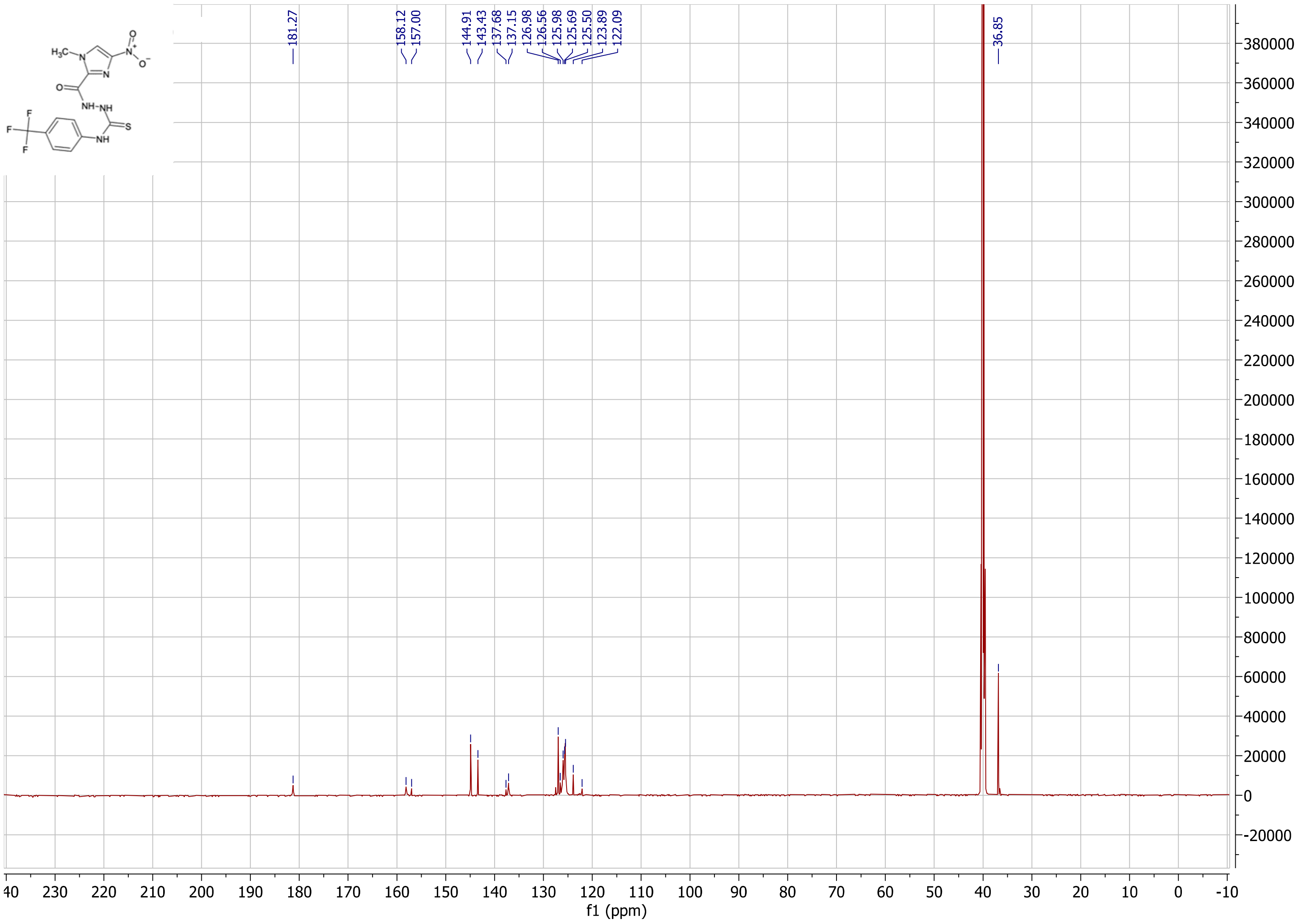

Figure S21. The ^{13}C NMR of compound **14**.

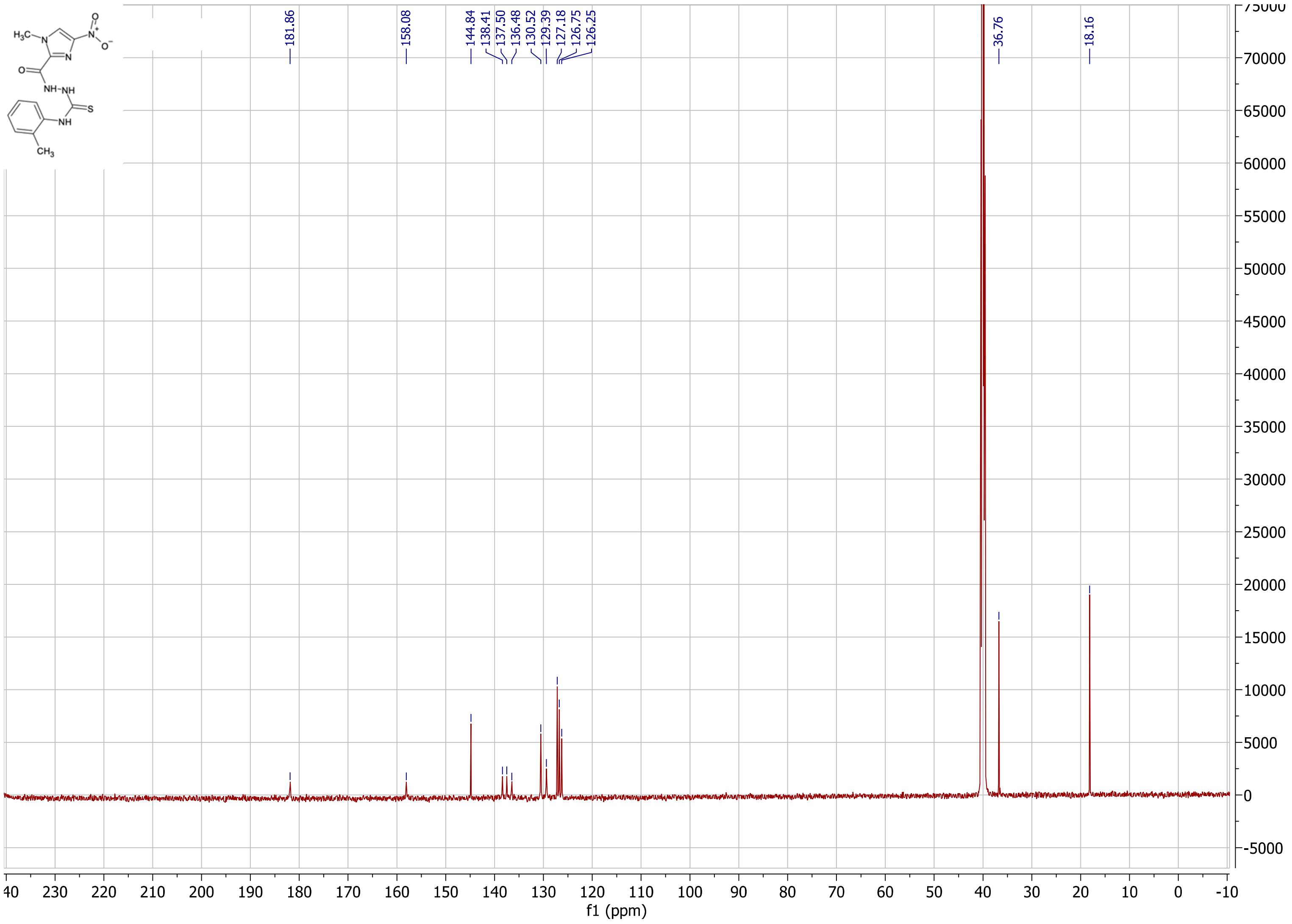

Figure S22. The ^{13}C NMR of compound **15**.

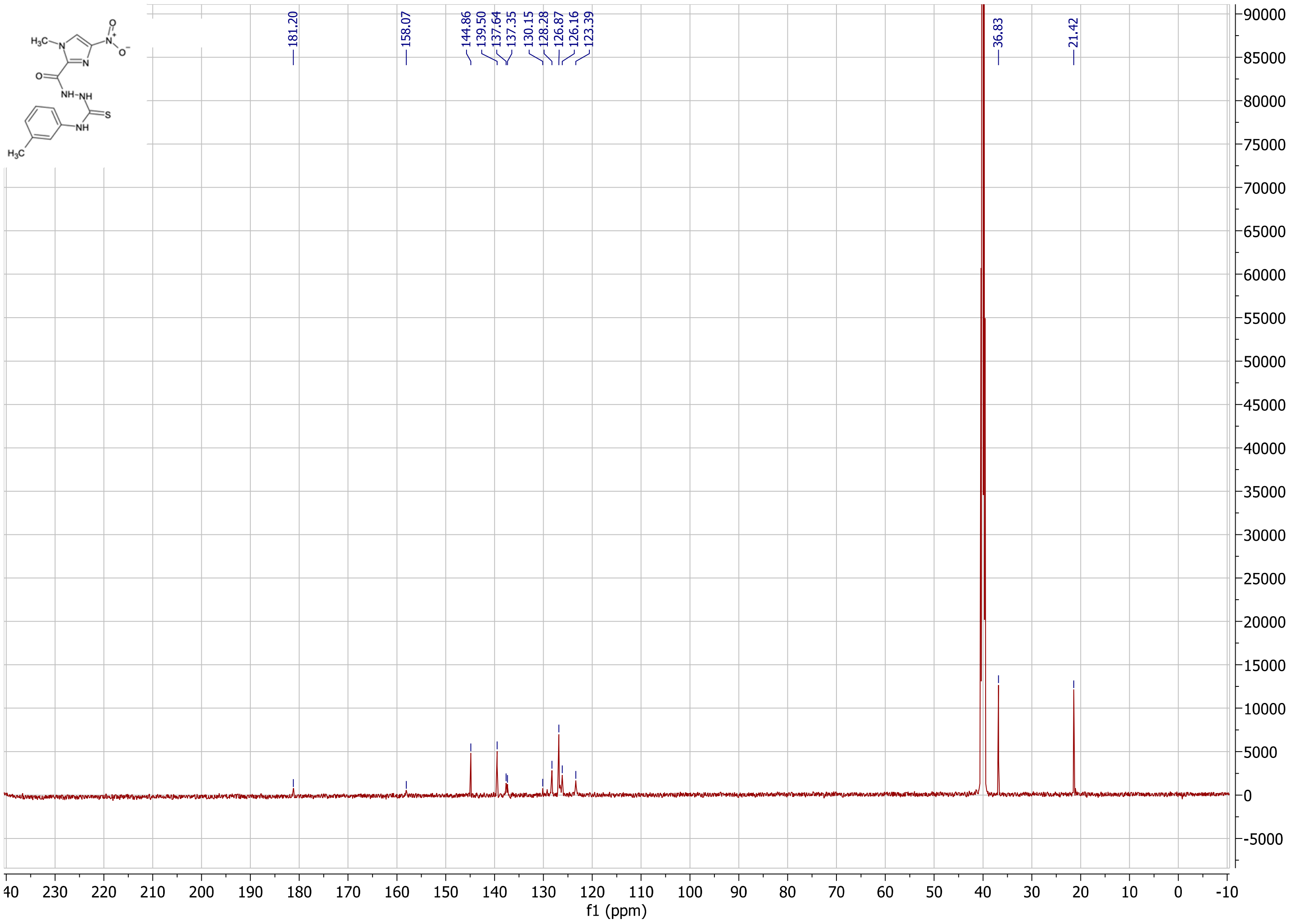

Figure S23. The ^{13}C NMR of compound **16**.

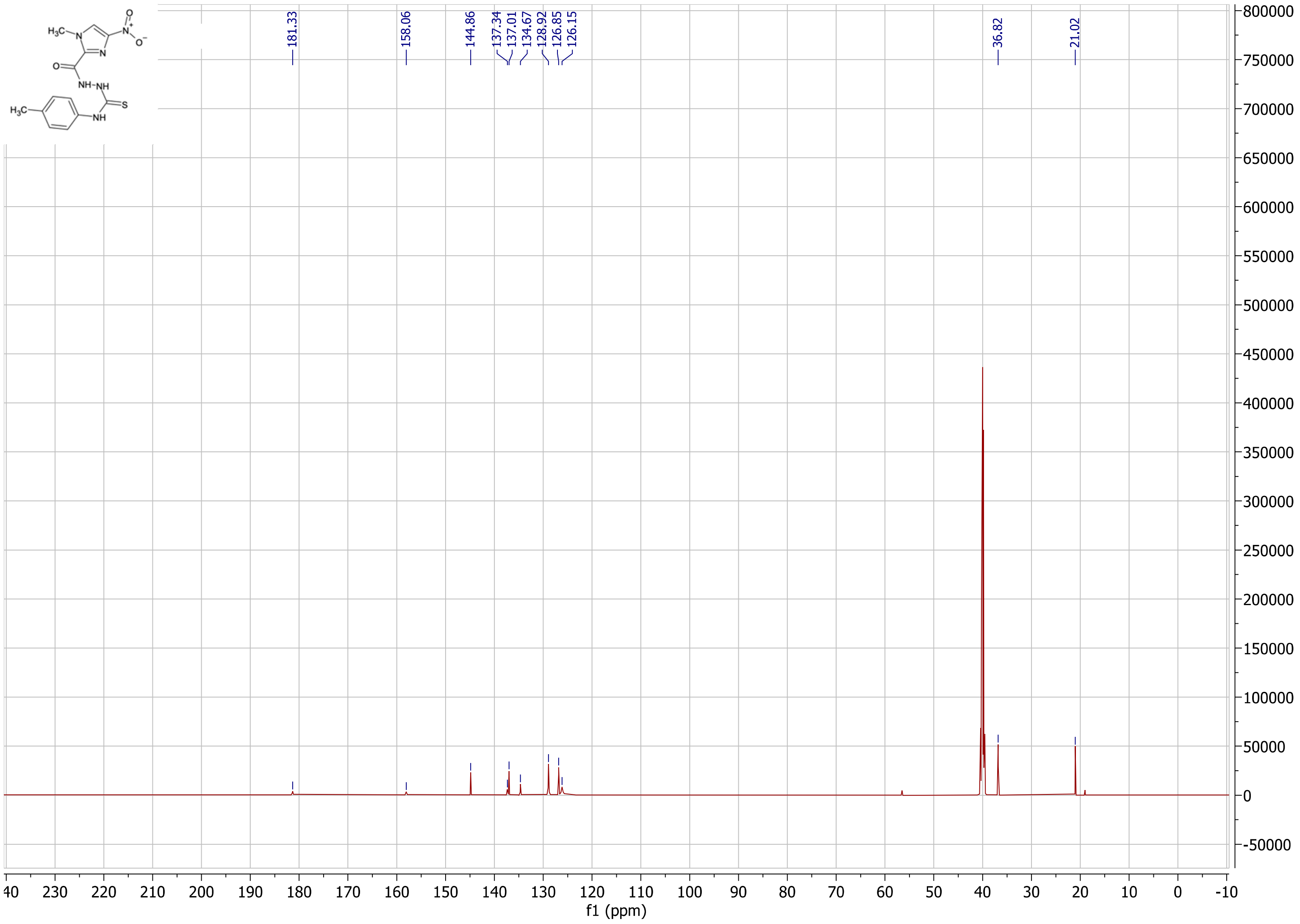

Figure S24. The ^{13}C NMR of compound **17**.

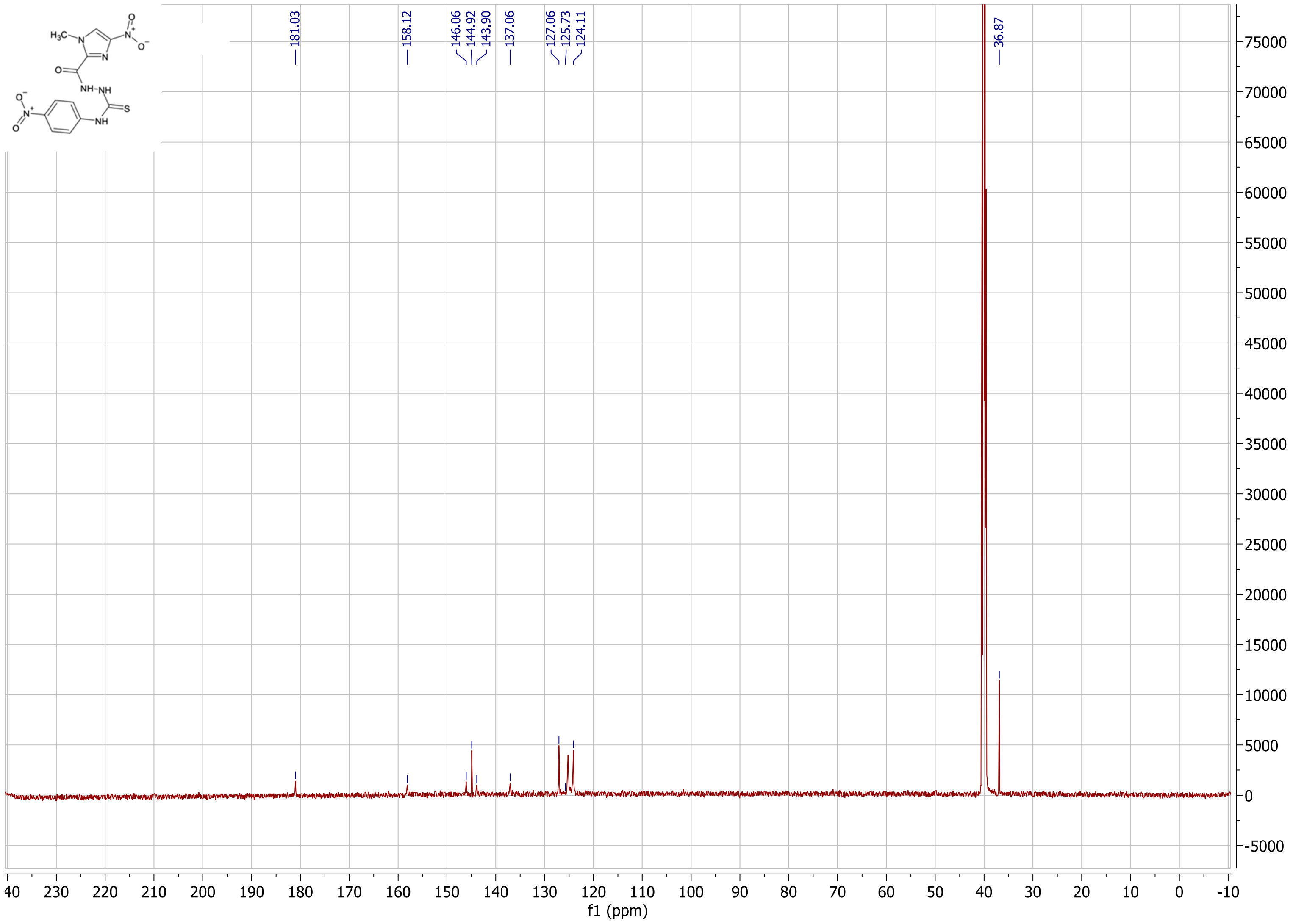

Figure S25. The ^{13}C NMR of compound **18**.

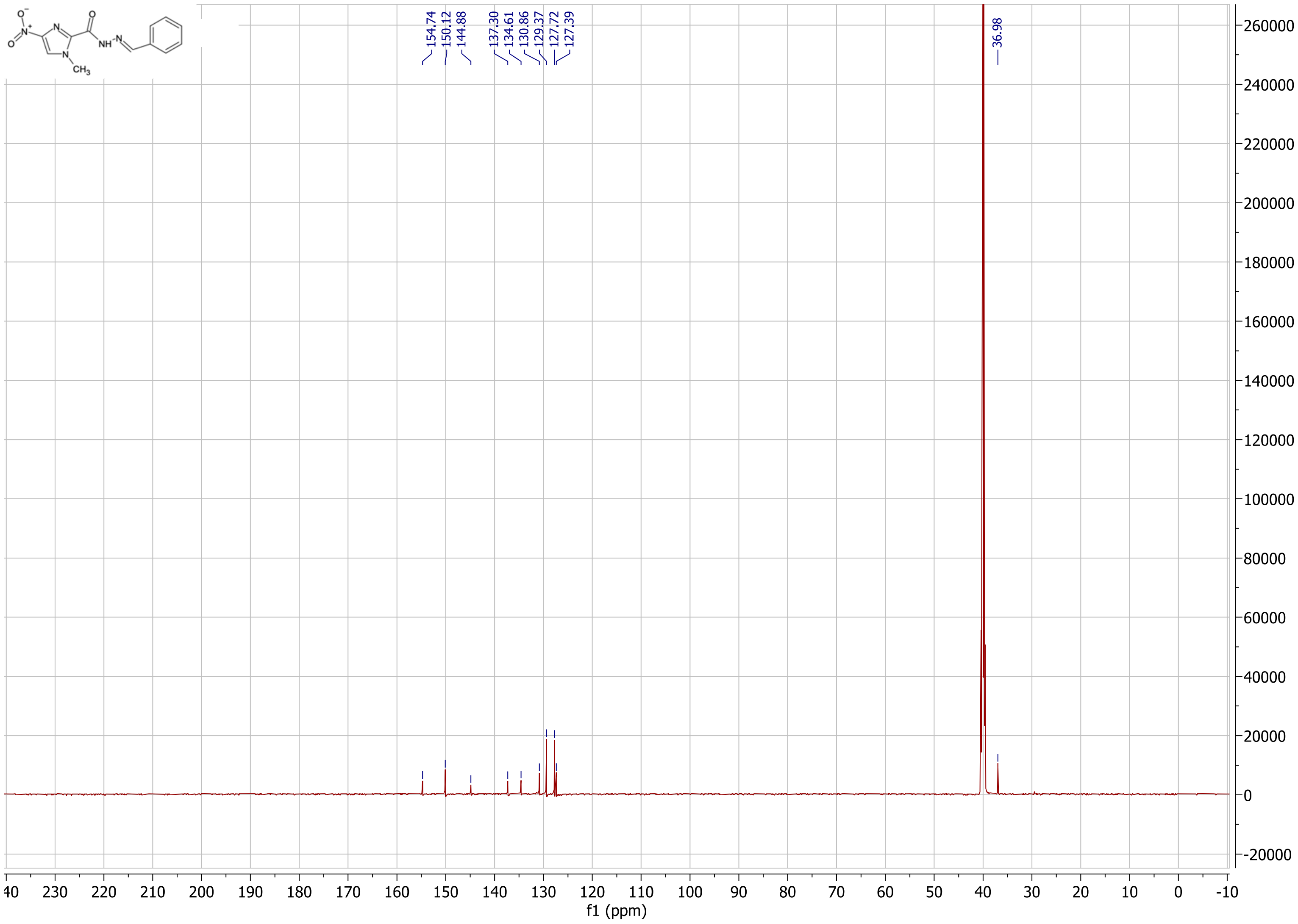

Figure S26. The ^{13}C NMR of compound **19**.

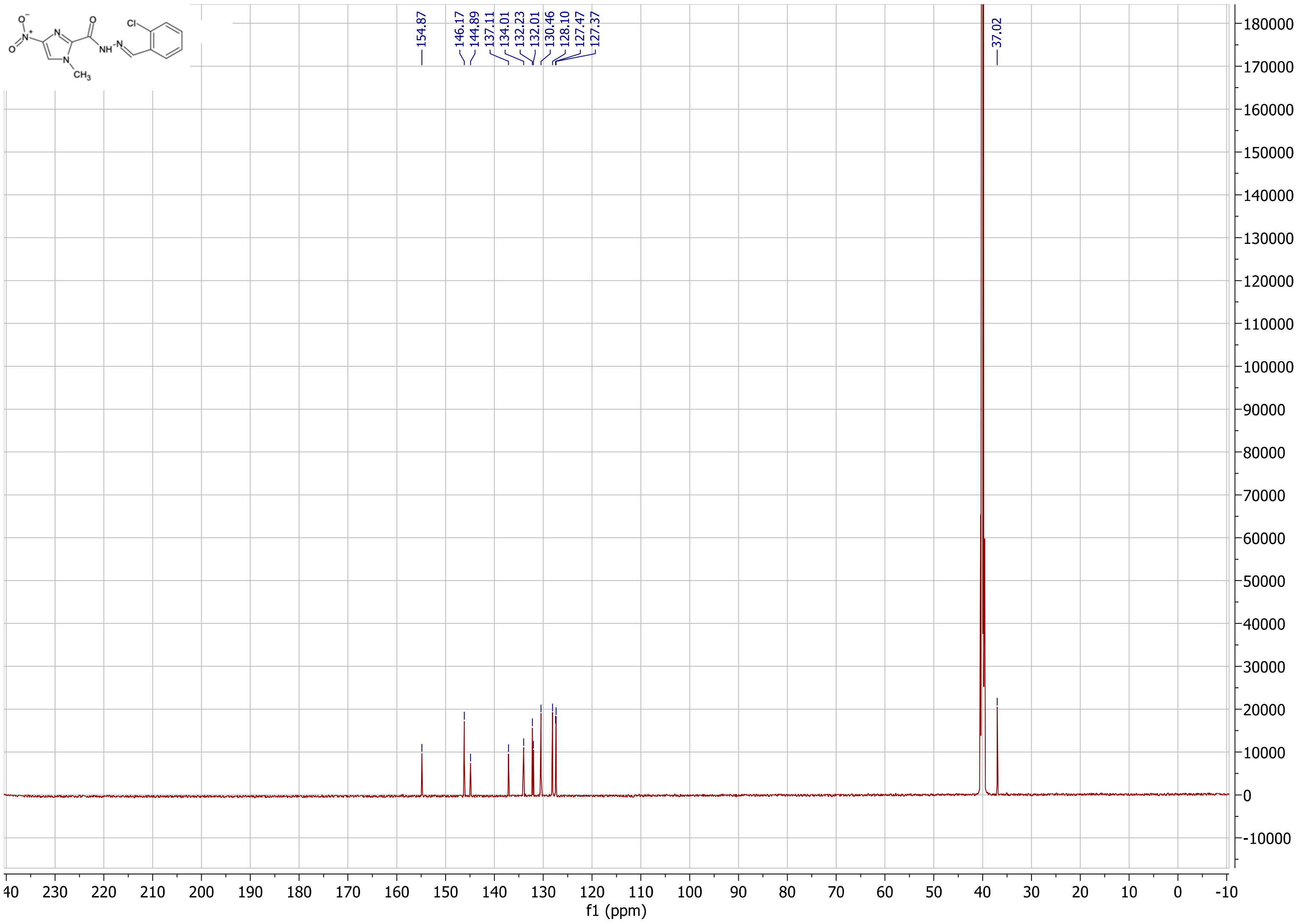

Figure S27. The ^{13}C NMR of compound **20**.

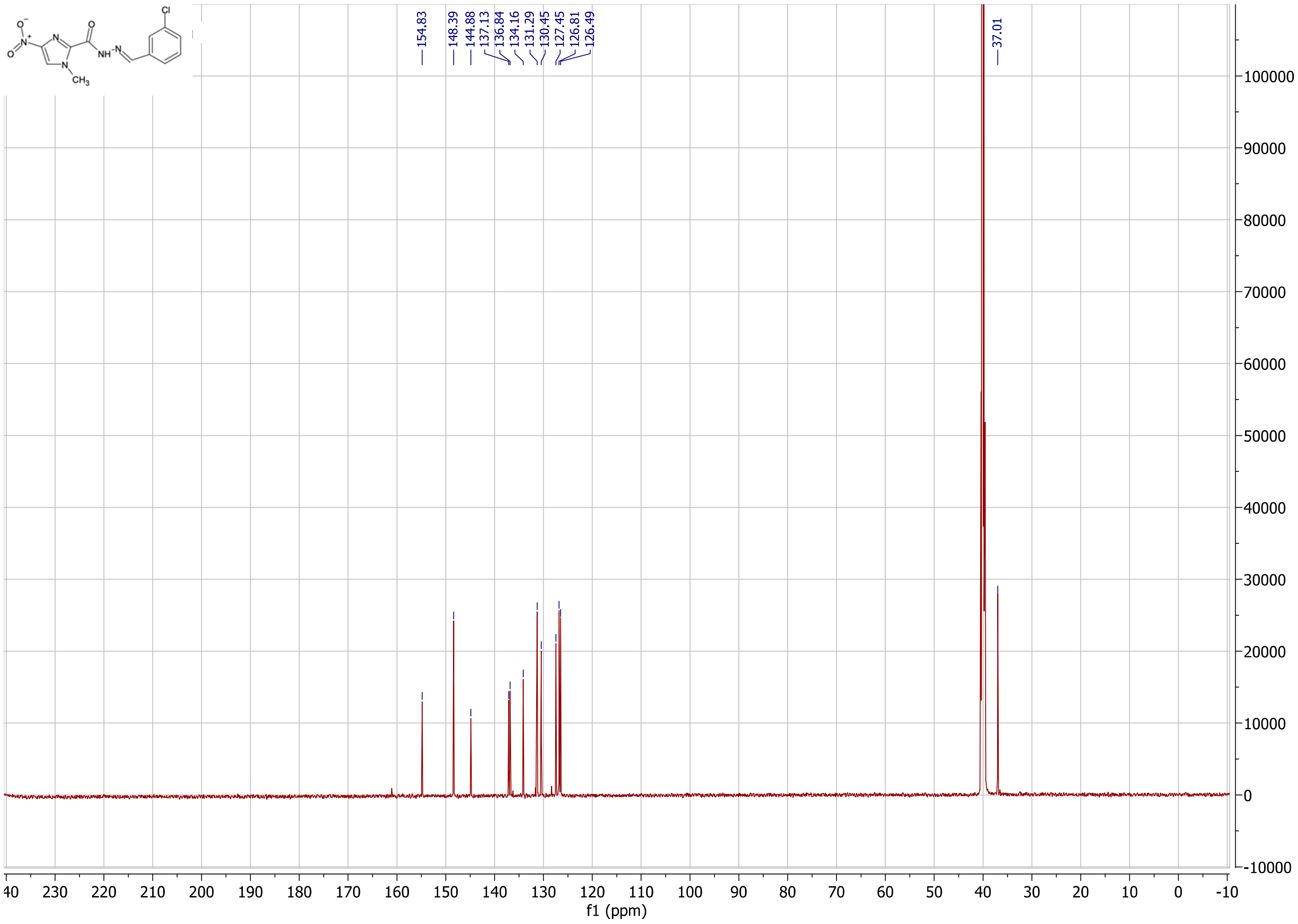

Figure S28. The ^{13}C NMR of compound **21**.

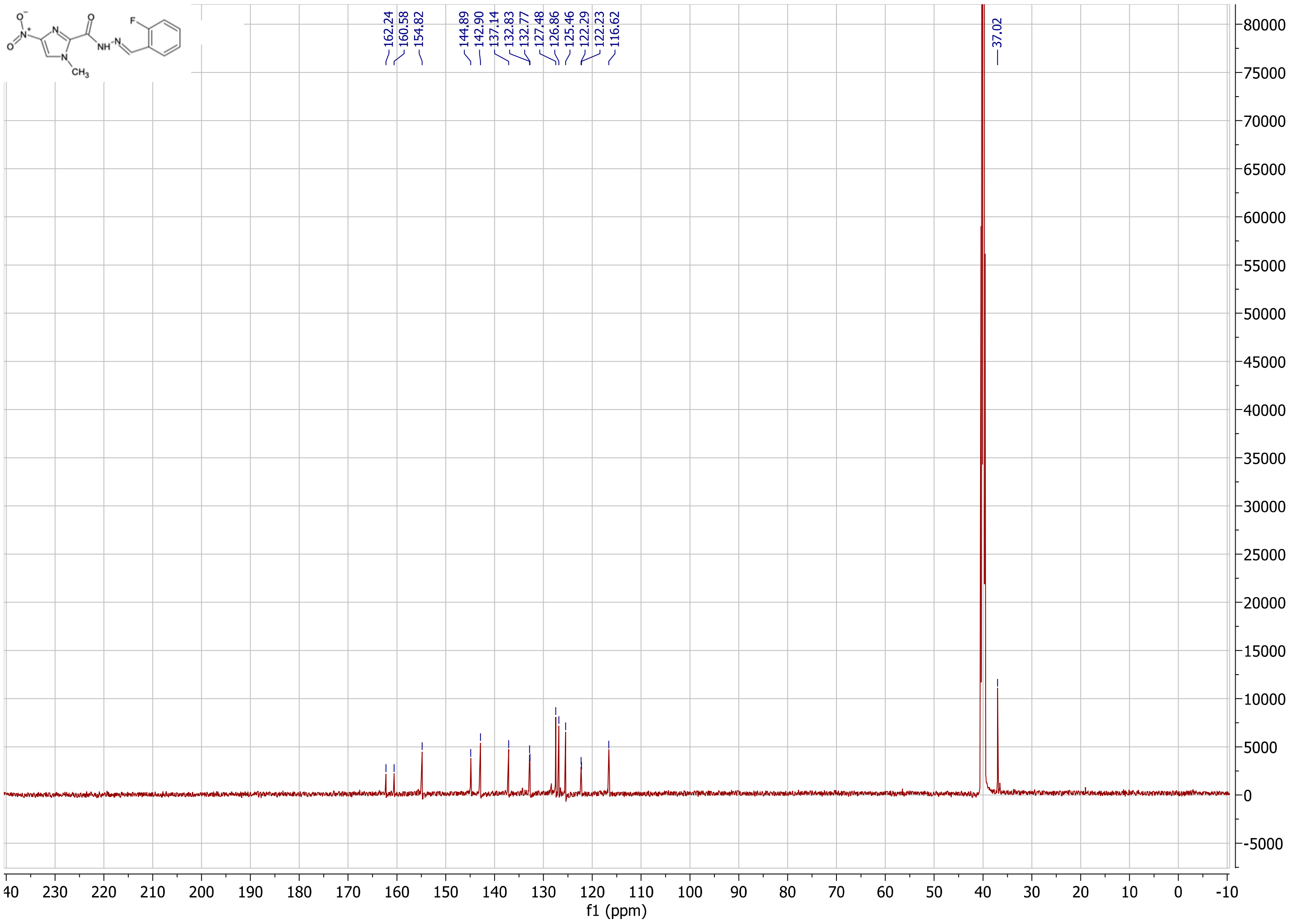

Figure S29. The ^{13}C NMR of compound **22**.

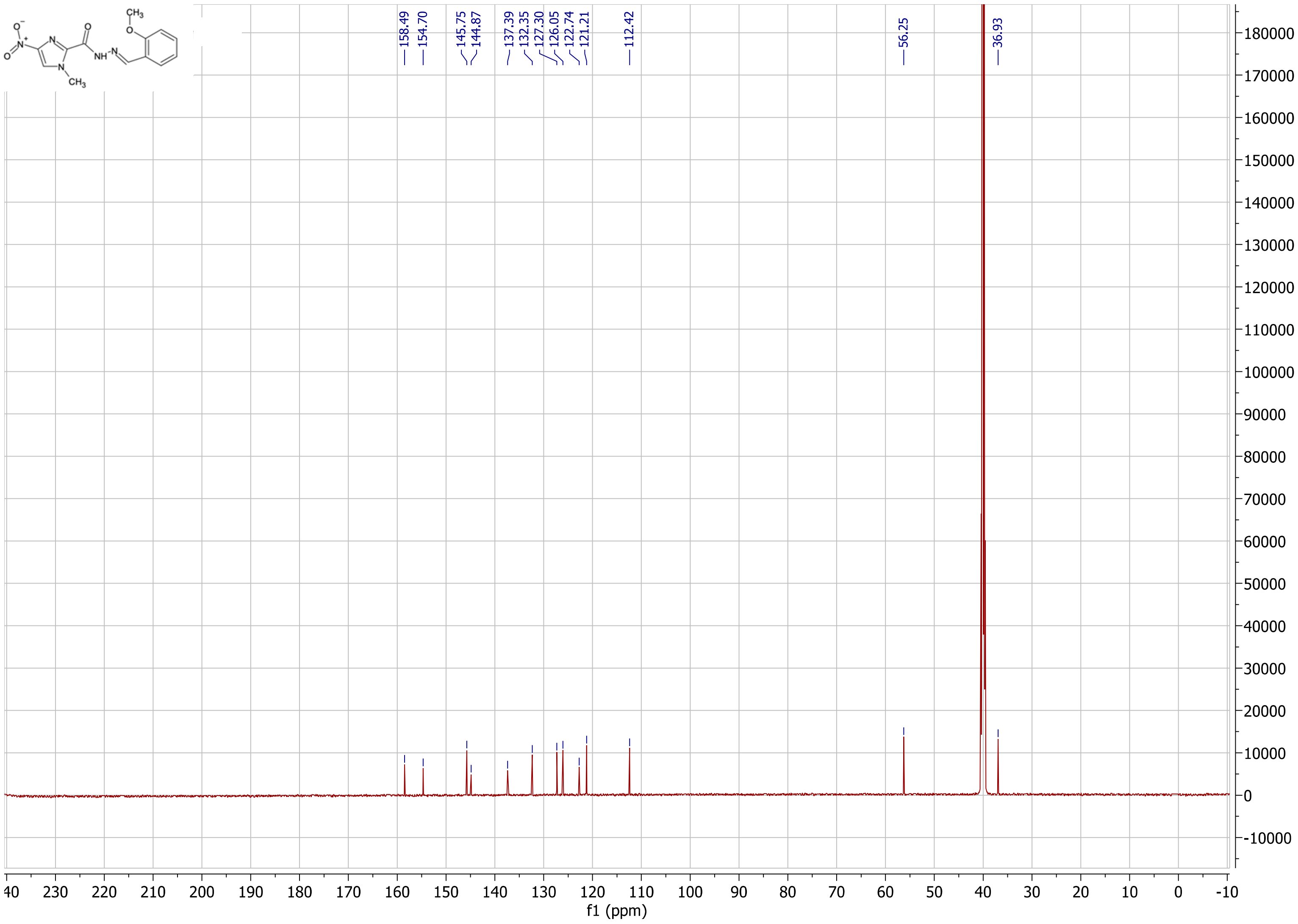

Figure S30. The ^{13}C NMR of compound **23**.

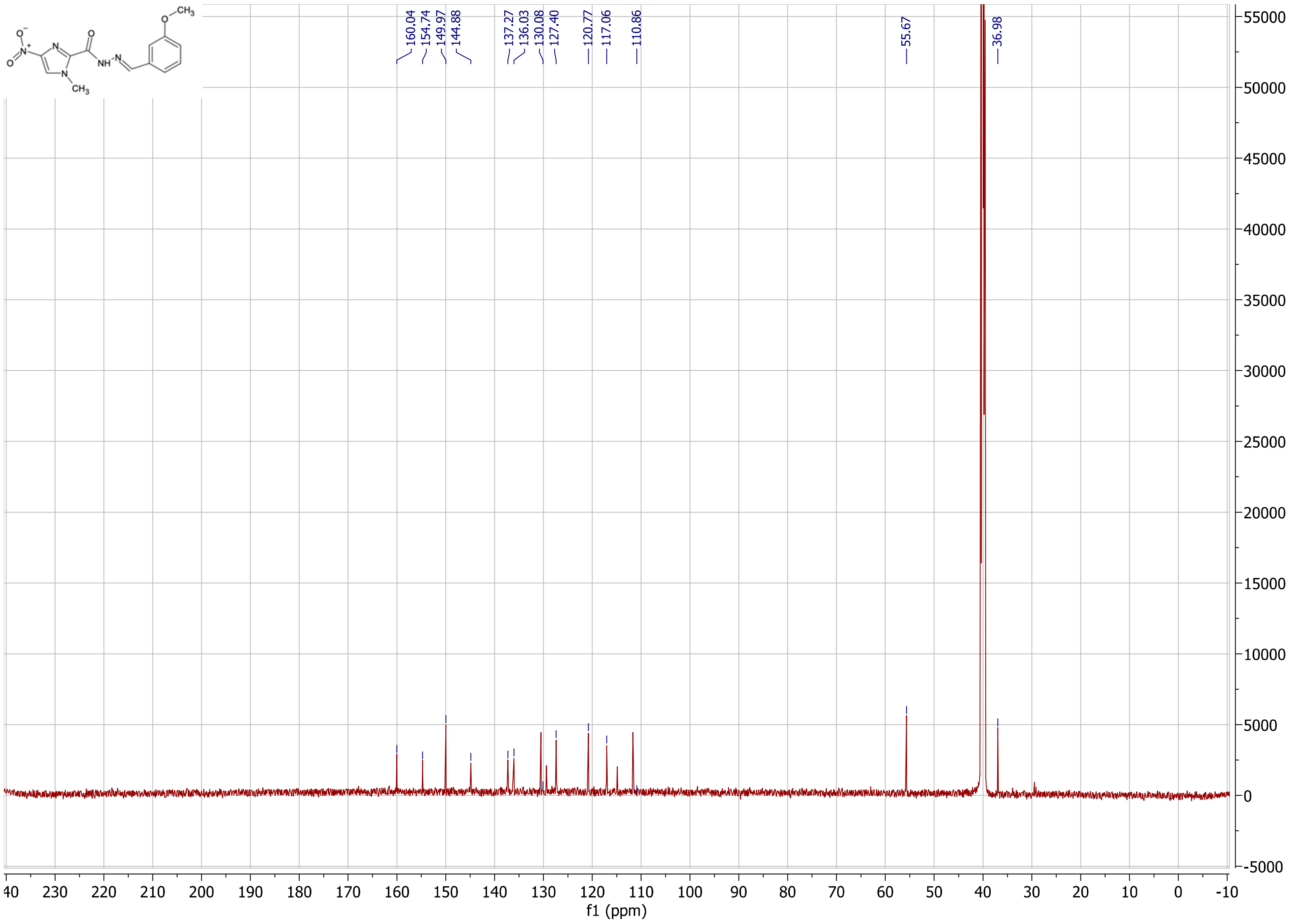

Figure S31. The ^{13}C NMR of compound **24**.

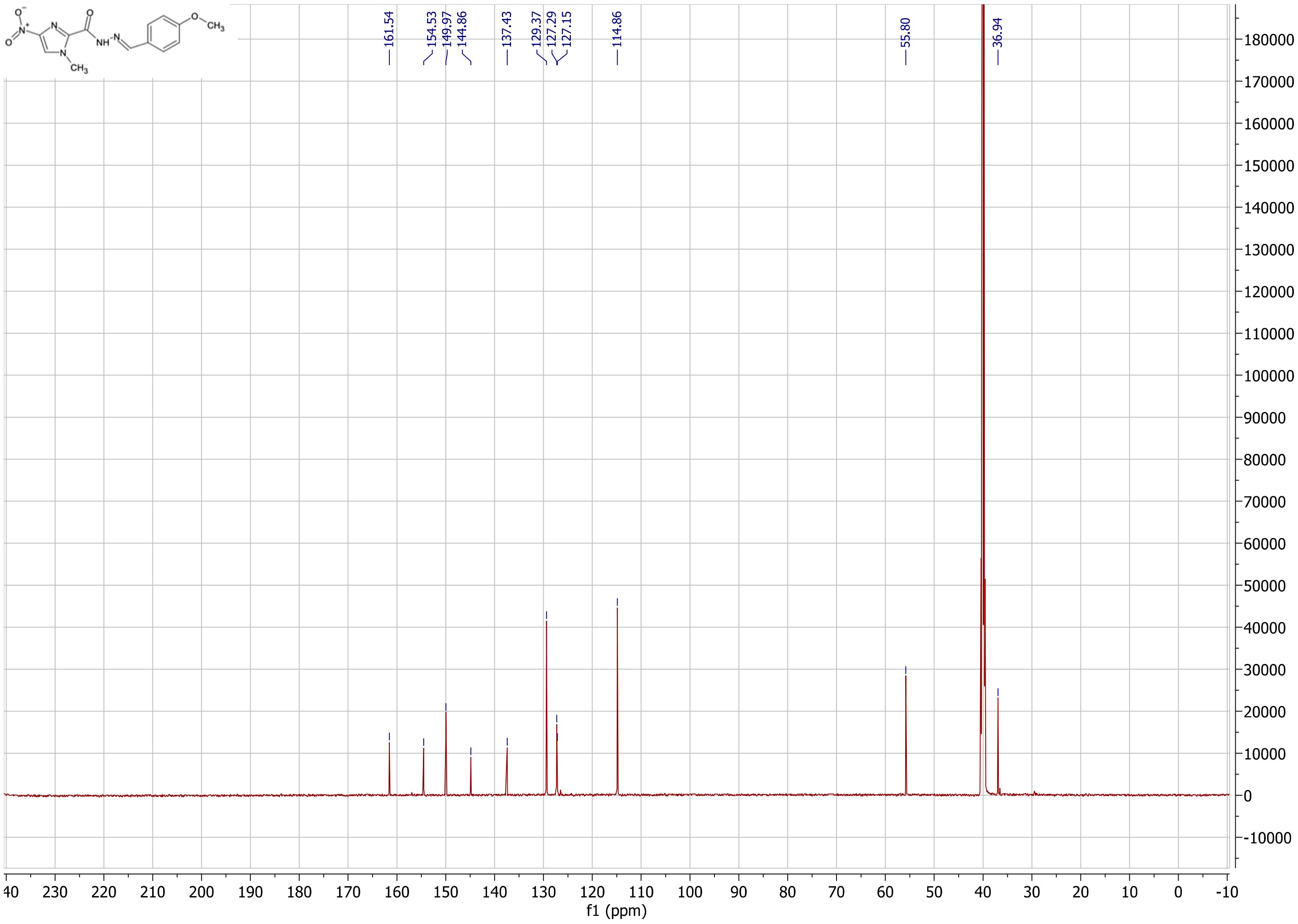

Figure S32. The ^{13}C NMR of compound **25**.

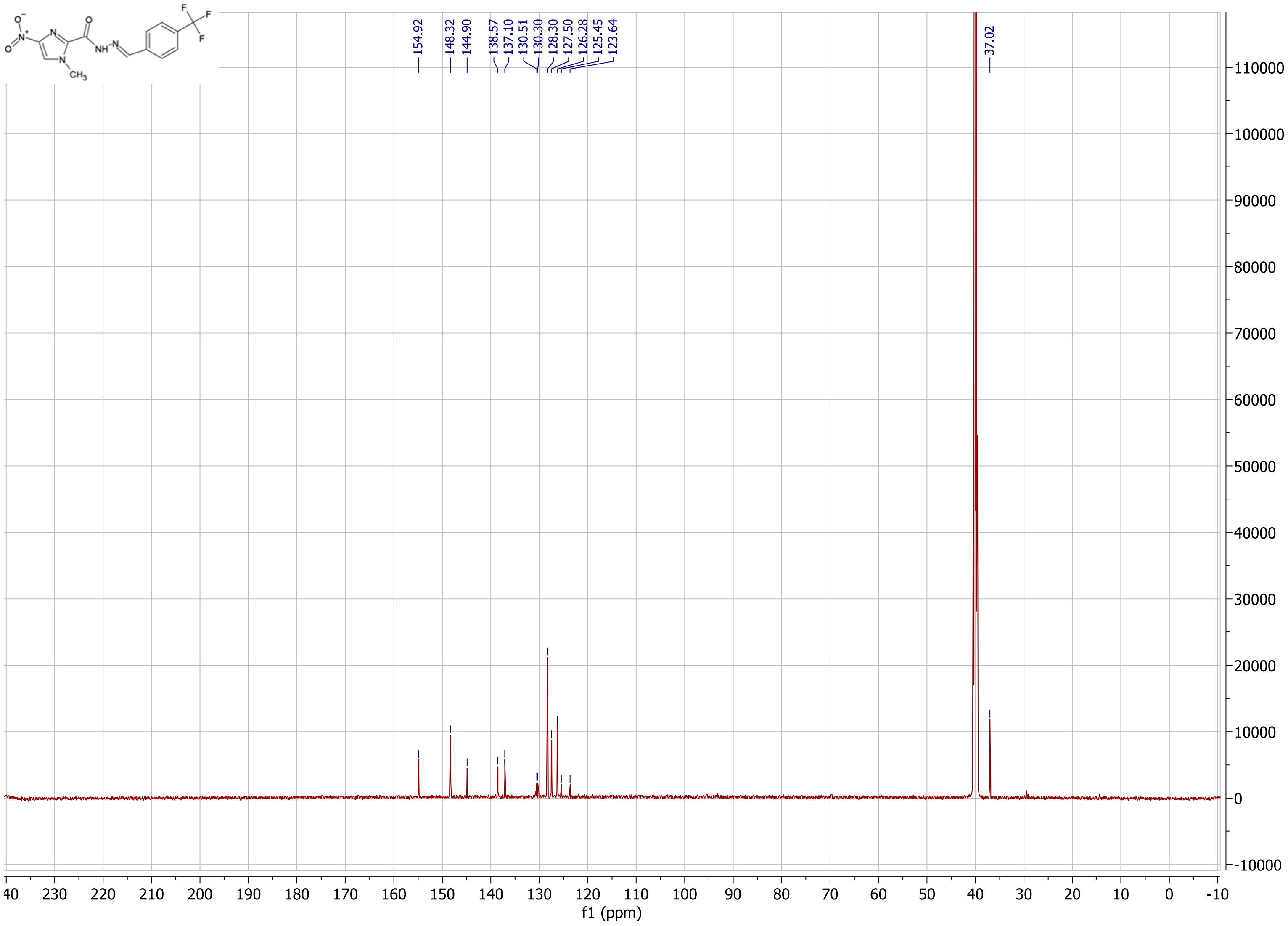

Figure S33. The ^{13}C NMR of compound **26**.

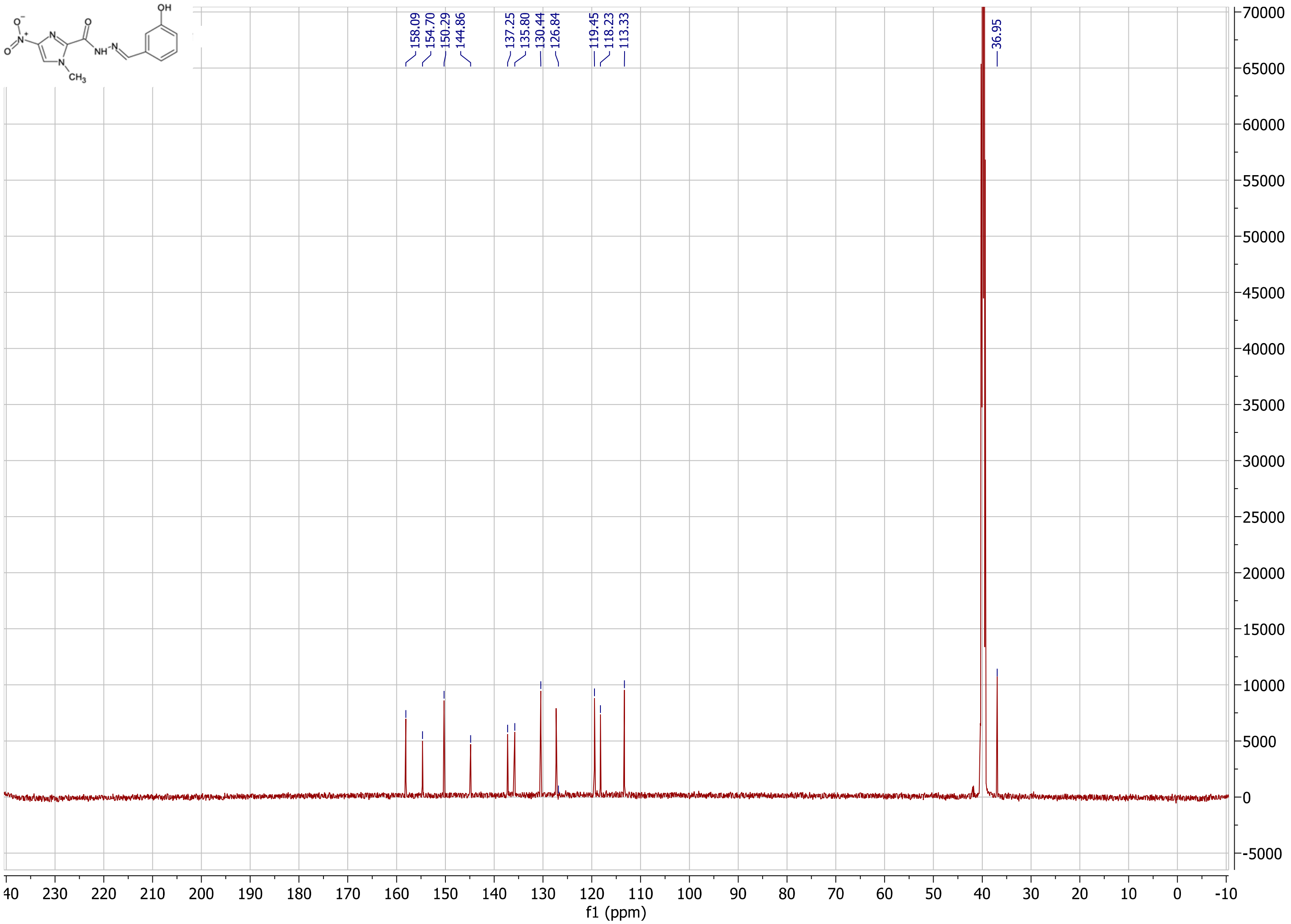

Figure S34. The ^{13}C NMR of compound **27**.

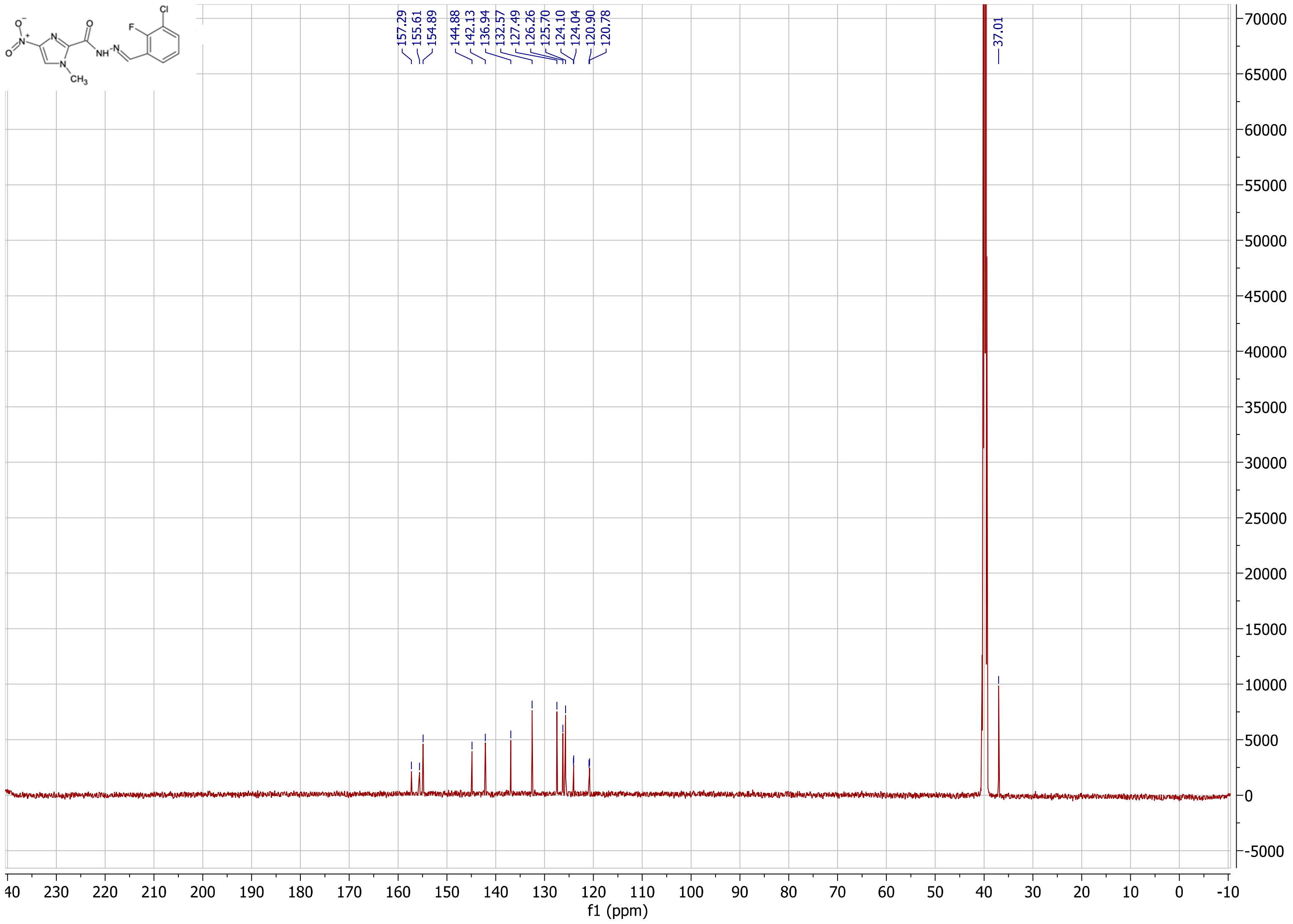

Figure S35. The ^{13}C NMR of compound **28**.

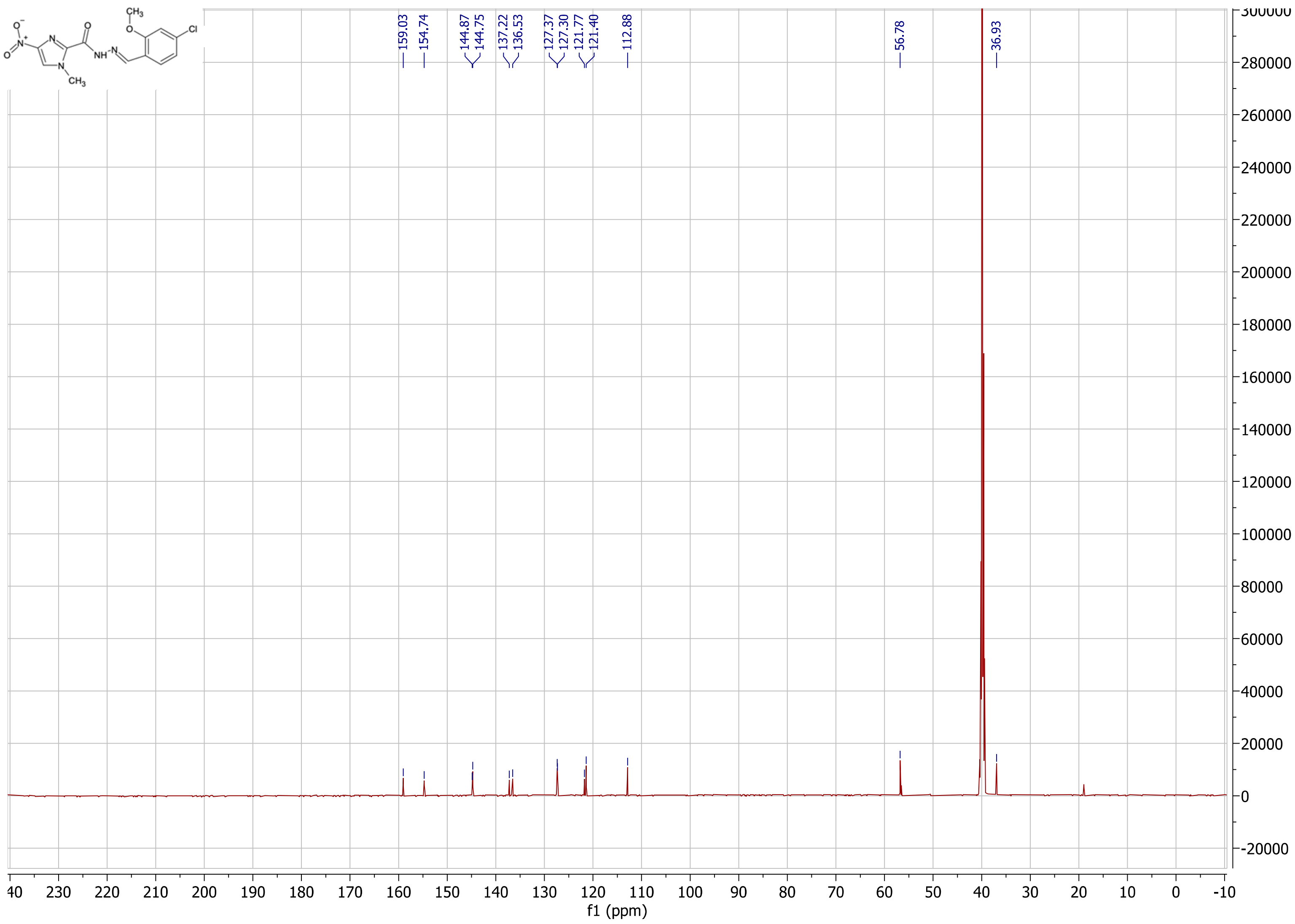

Figure S36. The ^{13}C NMR of compound **29**.

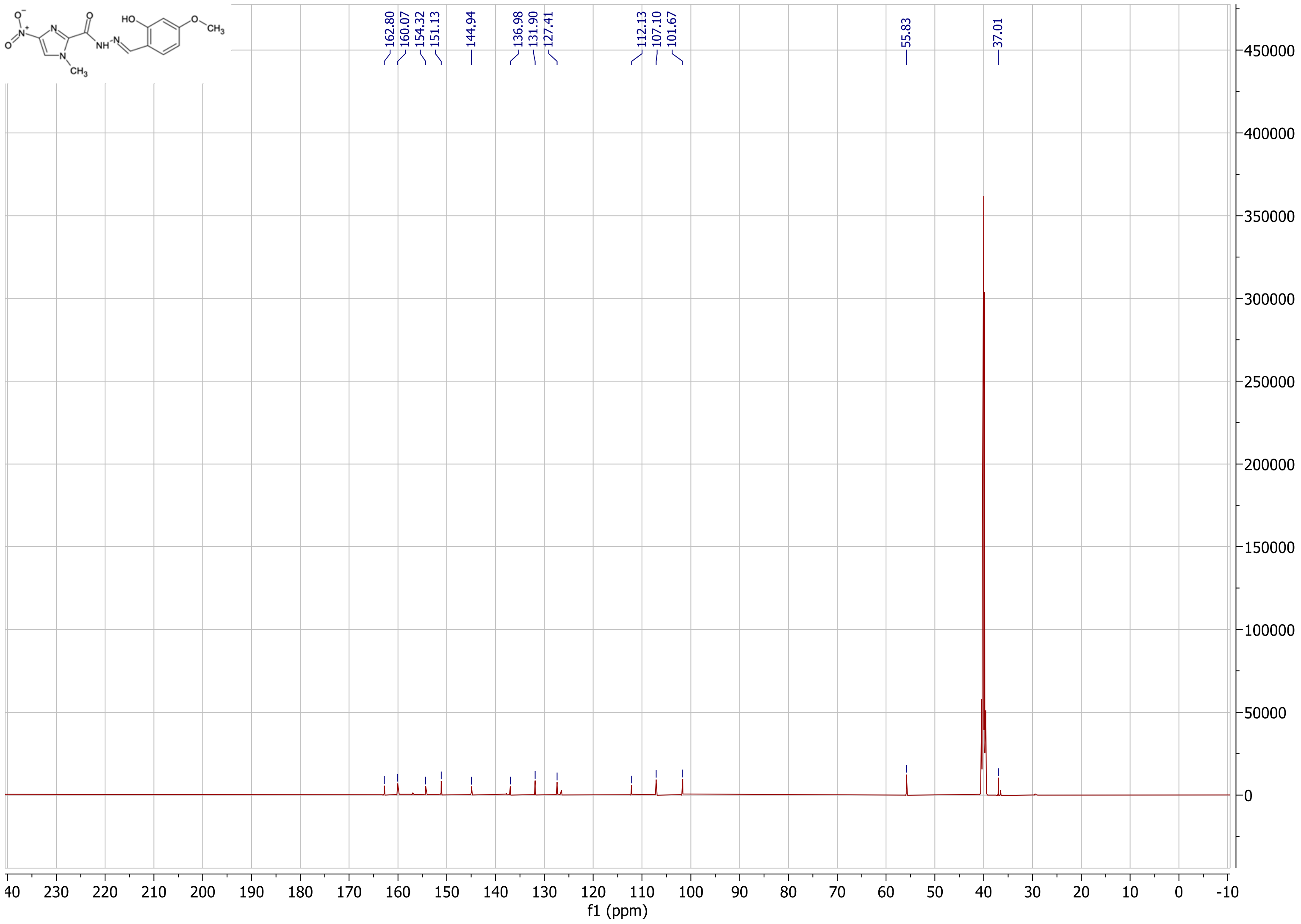

Figure S37. The ^{13}C NMR of compound **30**.

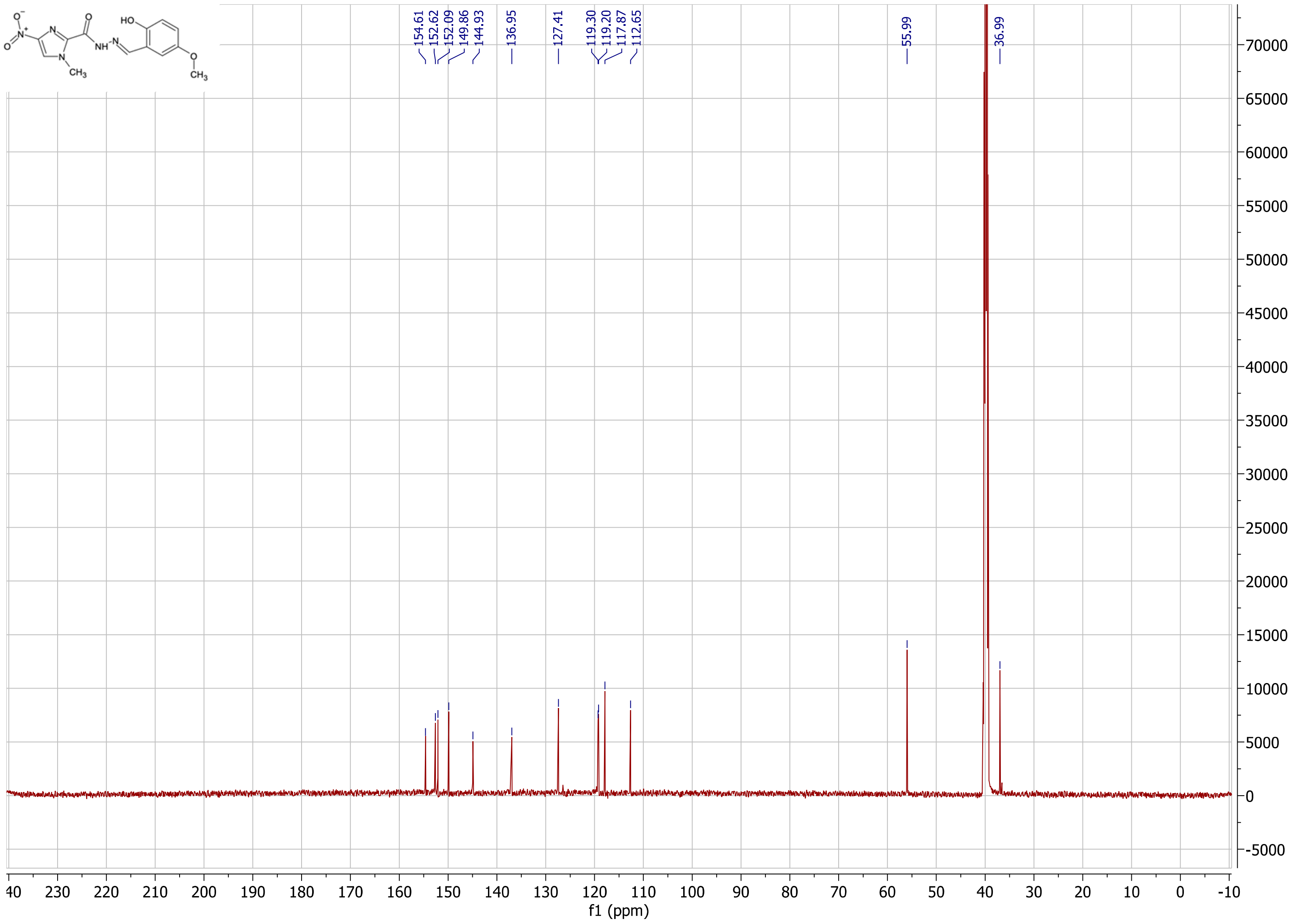

Figure S38. The ^{13}C NMR of compound **31**.

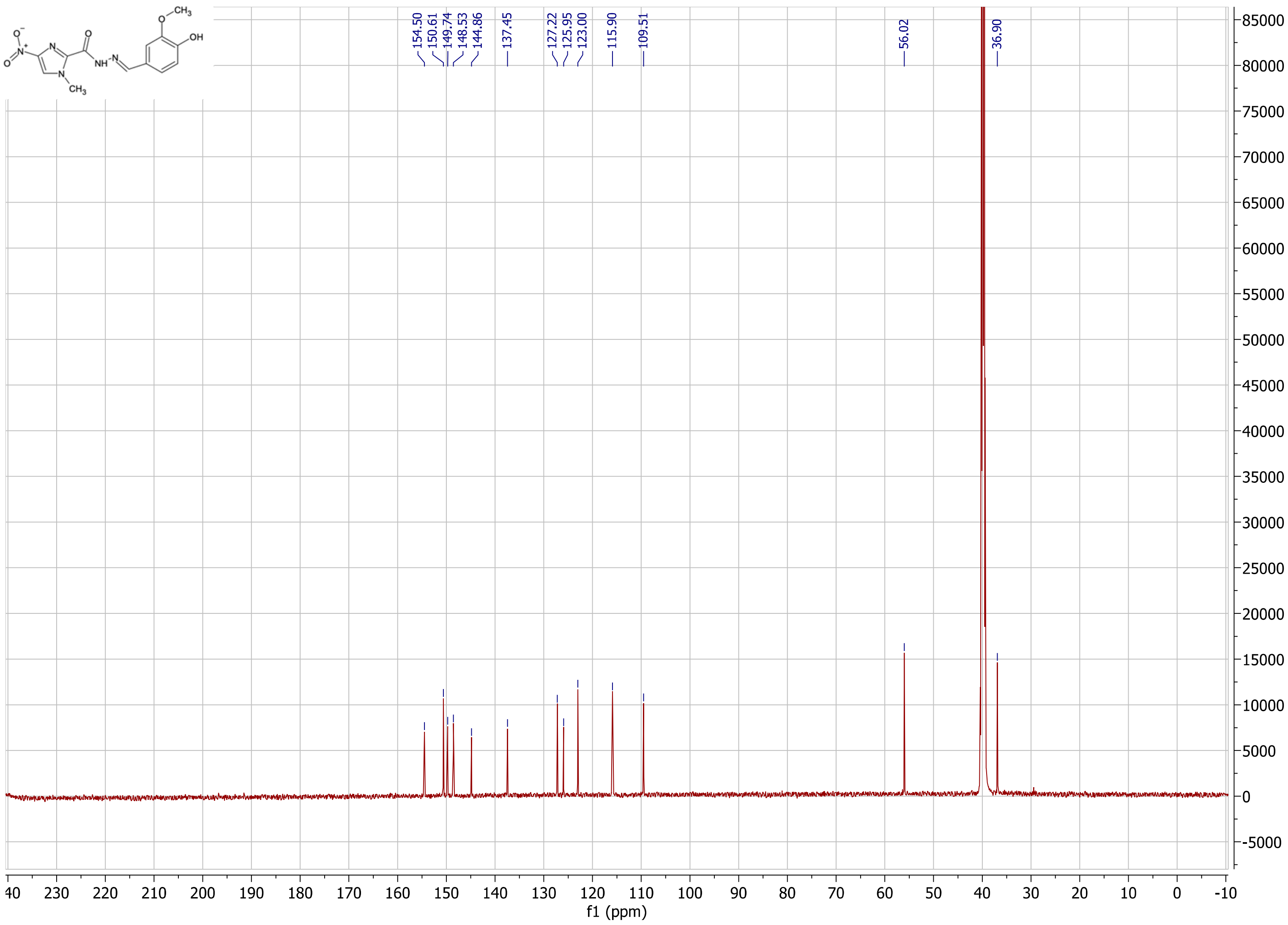

Figure S39. The ^{13}C NMR of compound **32**.

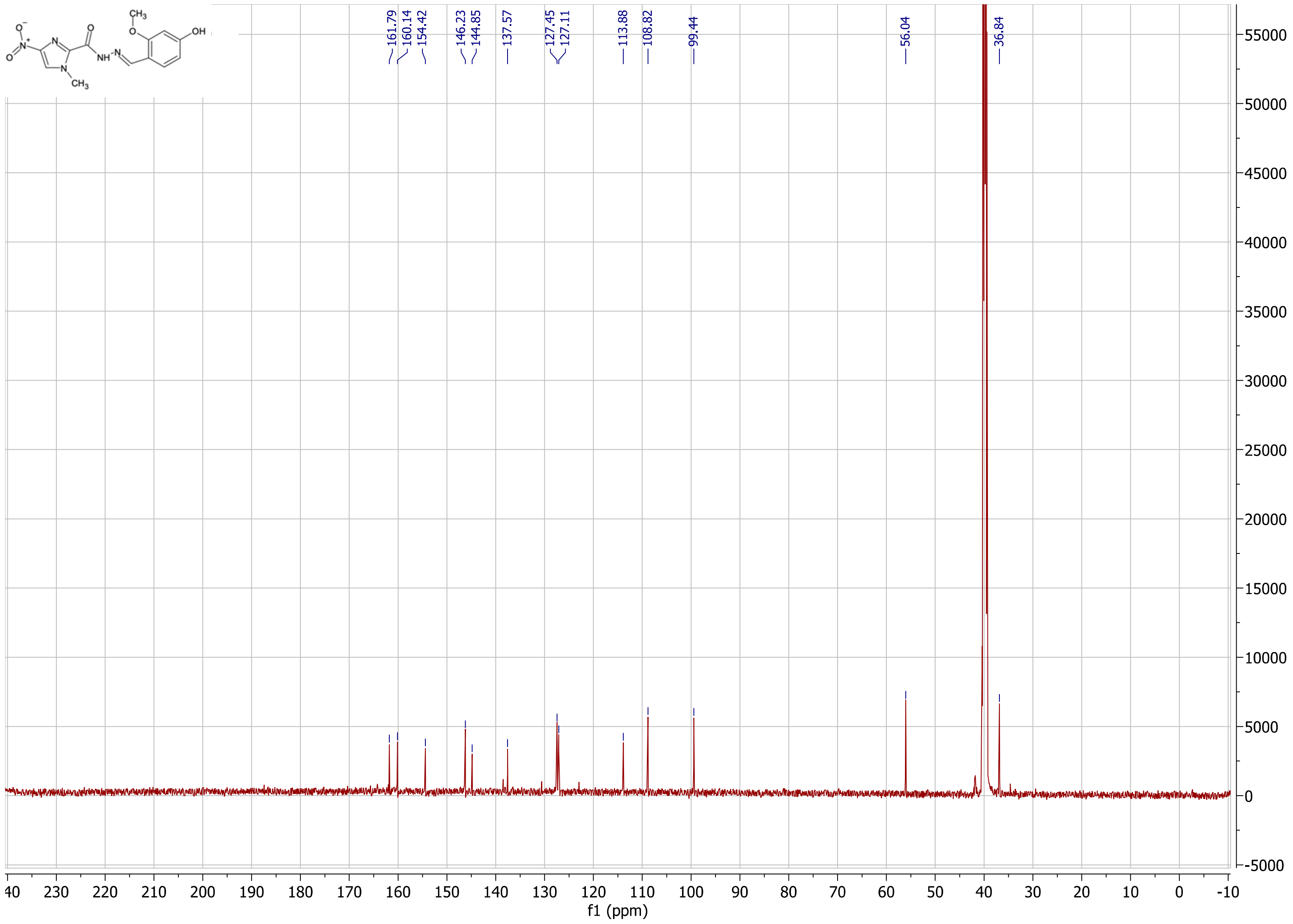

Figure S40. The ^{13}C NMR of compound **33**.

SHIMADZU LabSolutions

Analysis Report

Sample Name :
Sample ID :
Data Filename :
Method Filename : i-MeOH_70-15m-03-(100-800)_.lcm
Batch Filename : 14-6-2024.lcb
Vial # : 1-6 Sample Type : Unknown
Injection Volume : 0.1 uL
Date Acquired : 6/14/2024 6:24:18 PM Acquired by : System Administrator
Date Processed : 6/14/2024 6:39:19 PM Processed by : System Administrator

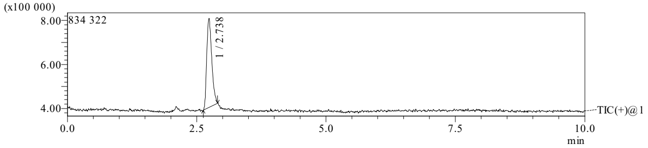

MASS Peak Table TIC

Peak#	Ret. Time	m/z	Area%
1	2.738	TIC	100.000
Total			100.000

MS Spectrum

Line#:1 R.Time:----(Scan#:----)
MassPeaks:422
Spectrum Mode:Averaged 2.730-2.740(547-549) Base Peak:389(198678)
BG Mode:Calc Segment 1 - Event 1

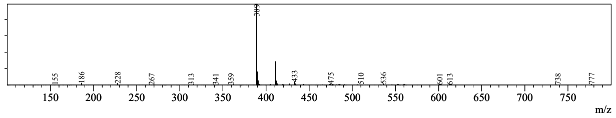

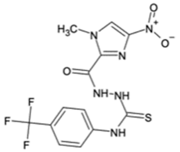

Formula Predictor Report

Printed at 15.06.2024 10:42:53

Formula Predictor Result	C13 H11 N6 O3 F3 S
Mass	389.06348
Error Margin	10 ppm
DBE Range	Not Used
Electron Ions	Both configurations
HC Ratio	Not Used
Nitrogen Rule	Used

#	Score	Pred. (M)	Pred. m/z	Meas. m/z	Diff. (mDa)	Formulae (M)	Ion	Diff. (ppm)	Iso Score	DBE
1	96.17	388.05654	389.06382	389.06348	-0.34	C13 H11 N6 O3 F3 S	[M+H]+	-0.875	95.74	10.0

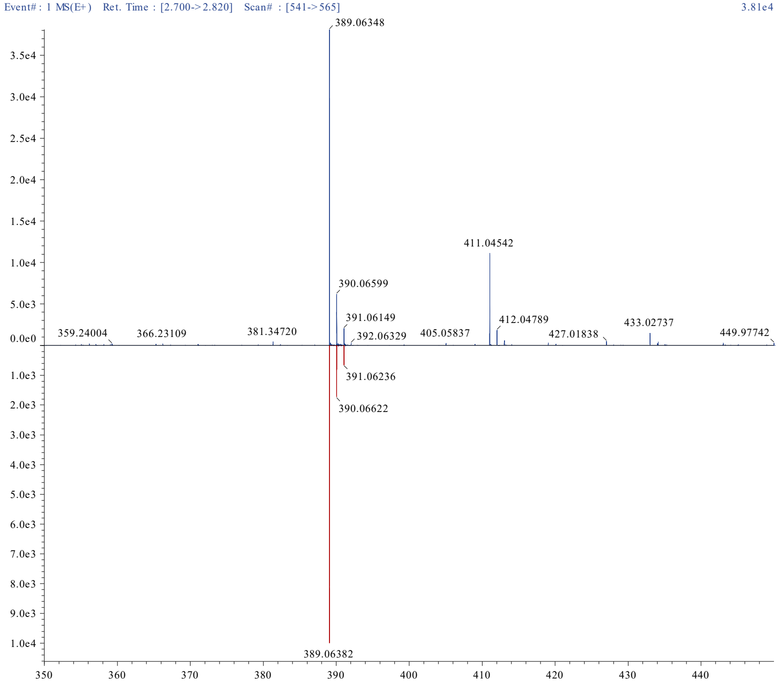

C:\LabSolutions\Data\DATA\KD-18-_i-MeOH_70-15m-03-(100-800)__14-6-2024_1.lcd

Figure S41. The MS of compound **14**.

SHIMADZU LabSolutions

Analysis Report

Sample Name	:		
Sample ID	:		
Data Filename	:		
Method Filename	: MeOH_70-30m-03_.lcm		
Batch Filename	: 17-6-2024.lcb		
Vial #	: 1-28	Sample Type	: Unknown
Injection Volume	: 0.6 uL		
Date Acquired	: 6/17/2024 2:17:40 PM	Acquired by	: System Administrator
Date Processed	: 6/17/2024 2:35:31 PM	Processed by	: System Administrator

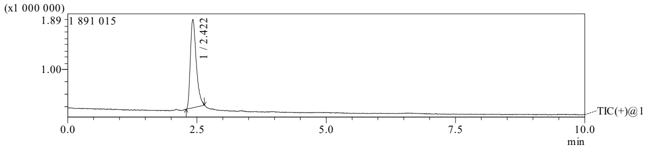

MASS Peak Table TIC

Peak#	Ret. Time	m/z	Area%
1	2.422	TIC	100.000
Total			100.000

MS Spectrum

Line#:1 R.Time:----(Scan#:----)
MassPeaks:700
Spectrum Mode:Averaged 2.415-2.425(484-486) Base Peak:335(373573)
BG Mode:Calc Segment 1 - Event 1

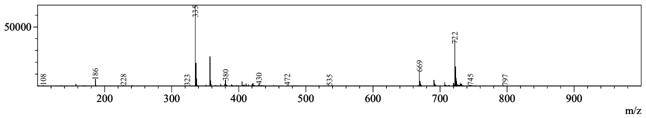

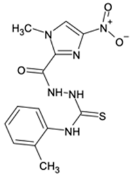

Formula Predictor Report

Printed at 17.06.2024 14:38:14

Formula Predictor Result	C13 H14 N6 O3 S
Mass	335.091774865
Error Margin	10 ppm
DBE Range	Not Used
Electron Ions	Both configurations
HC Ratio	Not Used
Nitrogen Rule	Used

#	Score	Pred. (M)	Pred. m/z	Meas. m/z	Diff. (mDa)	Formulae (M)	Ion	Diff. (ppm)	Iso Score	DBE
1	94.92	334.08481	335.09209	335.09177	-0.31	C13 H14 N6 O3 S	[M+H]+	-0.928	94.36	10.0

Event#: 1 MS(E+) Ret. Time : [2.375->2.570]-[3.145->4.575] Scan# : [476->515]-[630->916] 5.18e4

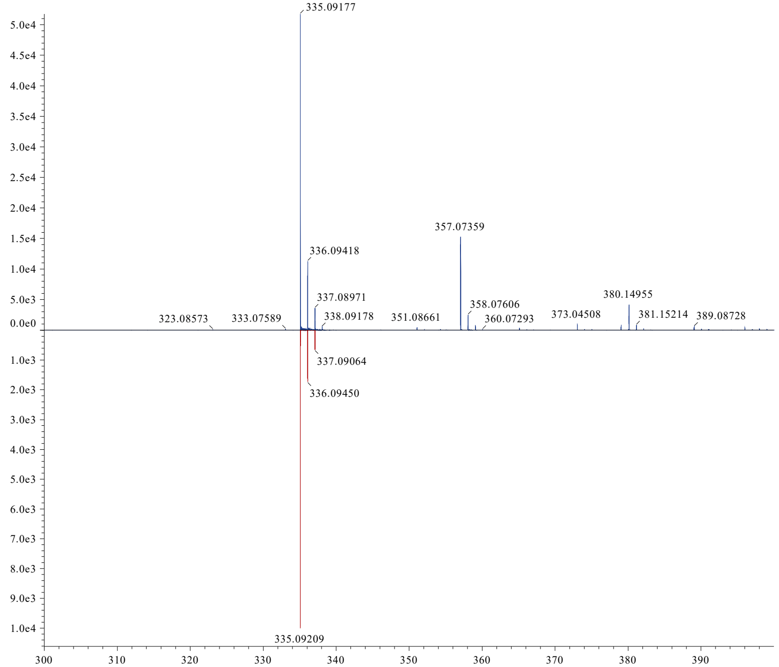

ts([C13 H14 N6 O3 S+H]+) 1.00e4

C:\LabSolutions\Data\DATA\KD-14New_MeOH_70-30m-03__17-6-2024_2.lcd

Figure S42. The MS of compound **15**.

Analysis Report

SHIMADZU LabSolutions

Sample Name	:		
Sample ID	:		
Data Filename	:		
Method Filename	: MeOH_70-30m-03_.lcm		
Batch Filename	: 14-6-2024.lcb		
Vial #	: 1-3	Sample Type	: Unknown
Injection Volume	: 0.1 uL		
Date Acquired	: 6/14/2024 5:15:30 PM	Acquired by	: System Administrator
Date Processed	: 6/14/2024 5:27:30 PM	Processed by	: System Administrator

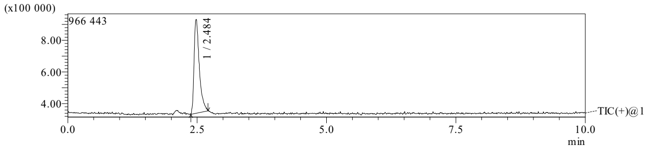

MASS Peak Table TIC

Peak#	Ret. Time	m/z	Area%
1	2.484	TIC	100.000
Total			100.000

MS Spectrum

Line#:1 R.Time:----(Scan#:----)
MassPeaks:416
Spectrum Mode:Averaged 2.475-2.485(496-498) Base Peak:335(200845)
BG Mode:Calc Segment 1 - Event 1

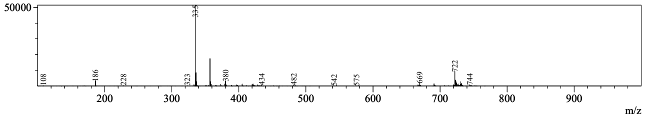

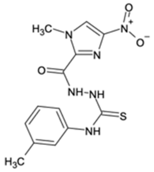

Formula Predictor Report

Printed at 15.06.2024 10:36:14

Formula Predictor Result	C13 H14 N6 O3 S
Mass	335.09156
Error Margin	10 ppm
DBE Range	Not Used
Electron Ions	Both configurations
HC Ratio	Not Used
Nitrogen Rule	Used

#	Score	Pred. (M)	Pred. m/z	Meas. m/z	Diff. (mDa)	Formulae (M)	Ion	Diff. (ppm)	Iso Score	DBE
2	87.94	334.08481	335.09209	335.09156	-0.53	C13 H14 N6 O3 S	[M+H]+	-1.570	86.60	10.0

Event# : 1 MS(E+) Ret. Time : [2.445->2.505] Scan# : [490->502] 4.72e4

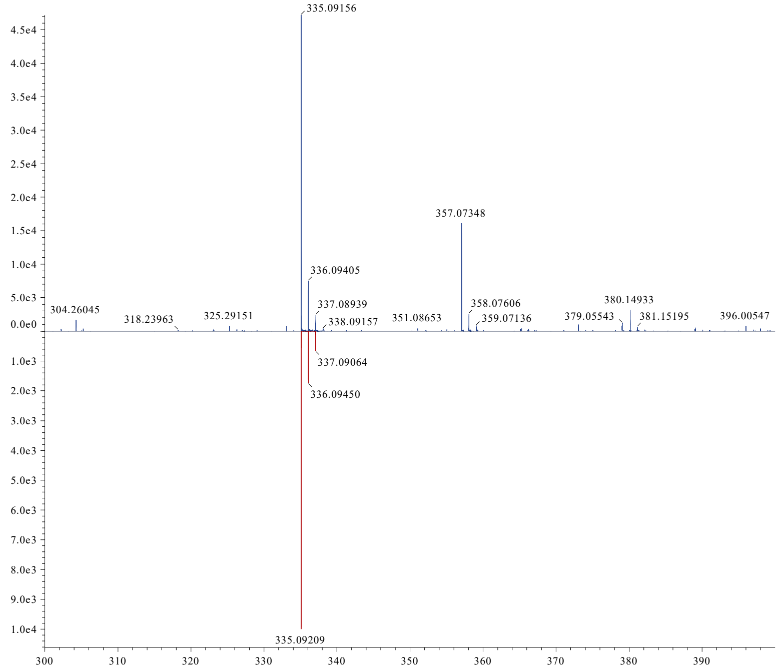

ts([C13 H14 N6 O3 S+H]+) 1.00e4

C:\LabSolutions\Data\DATA\KD-15-_MeOH_70-30m-03__14-6-2024_3.lcd

Figure S43. The MS of compound **16**.

SHIMADZU LabSolutions

Analysis Report

Sample Name	:		
Sample ID	:		
Data Filename	:		
Method Filename	: i-MeOH_70-15m-03-(100-800)_.lcm		
Batch Filename	: 14-6-2024.lcb		
Vial #	: 1-4	Sample Type	: Unknown
Injection Volume	: 0.1 uL		
Date Acquired	: 6/14/2024 5:27:56 PM	Acquired by	: System Administrator
Date Processed	: 6/14/2024 5:42:57 PM	Processed by	: System Administrator

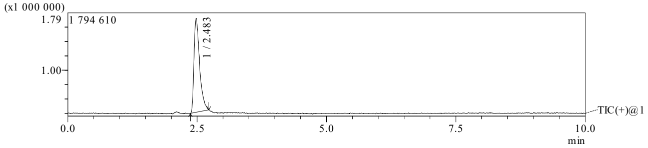

MASS Peak Table TIC

Peak#	Ret. Time	m/z	Area%
1	2.483	TIC	100.000
Total			100.000

MS Spectrum

Line#:1 R.Time:----(Scan#:----)
MassPeaks:673
Spectrum Mode:Averaged 2.475-2.485(496-498) Base Peak:335(390183)
BG Mode:Calc Segment 1 - Event 1

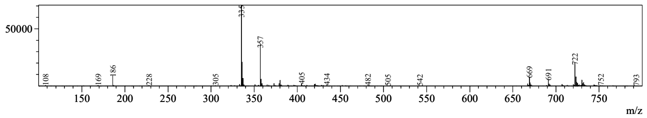

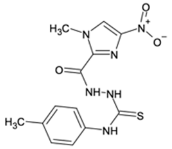

Formula Predictor Report

Printed at 15.06.2024 10:37:54

Formula Predictor Result	C13 H14 N6 O3 S
Mass	335.09167
Error Margin	10 ppm
DBE Range	Not Used
Electron Ions	Both configurations
HC Ratio	Not Used
Nitrogen Rule	Used

#	Score	Pred. (M)	Pred. m/z	Meas. m/z	Diff. (mDa)	Formulae (M)	Ion	Diff. (ppm)	Iso Score	DBE
4	87.50	334.08481	335.09209	335.09167	-0.42	C13 H14 N6 O3 S	[M+H]+	-1.241	86.11	10.0

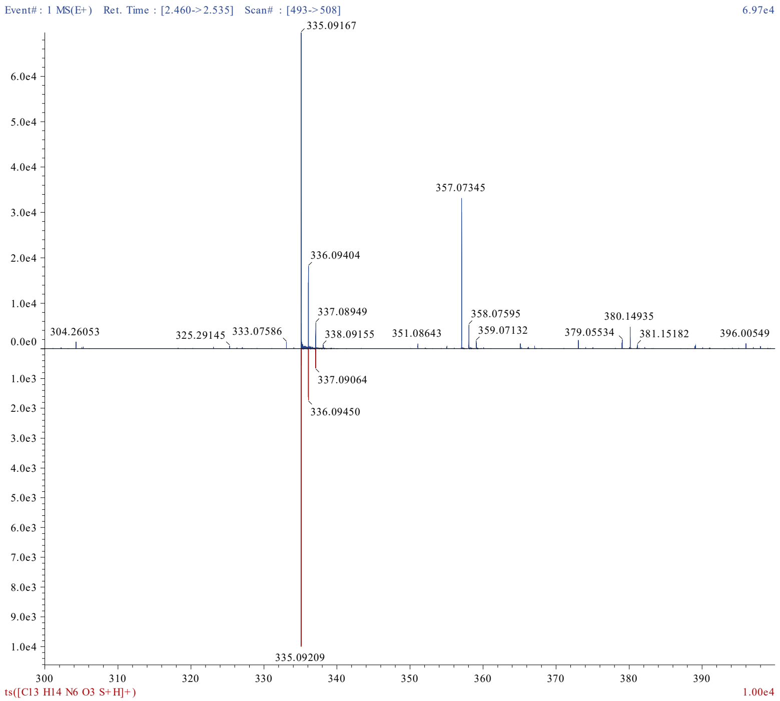

C:\LabSolutions\Data\DATA\KD-16-_i-MeOH_70-15m-03-(100-800)__14-6-2024_1.lcd

Figure S44. The MS of compound **17**.

SHIMADZU LabSolutions

Analysis Report

Sample Name	:		
Sample ID	:		
Data Filename	:		
Method Filename	: i-MeOH_70-15m-03-(100-800)_.lcm		
Batch Filename	: 14-6-2024.lcb		
Vial #	: 1-5	Sample Type	: Unknown
Injection Volume	: 0.1 uL		
Date Acquired	: 6/14/2024 5:43:22 PM	Acquired by	: System Administrator
Date Processed	: 6/14/2024 5:58:23 PM	Processed by	: System Administrator

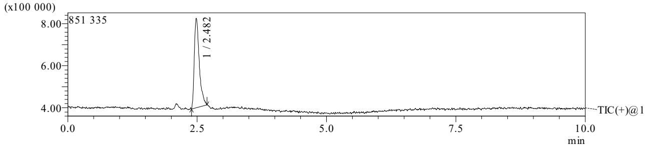

MASS Peak Table TIC

Peak#	Ret. Time	m/z	Area%
1	2.482	TIC	100.000
Total			100.000

MS Spectrum

Line#:1 R.Time:----(Scan#:----)
MassPeaks:424
Spectrum Mode:Averaged 2.475-2.485(496-498) Base Peak:366(175169)
BG Mode:Calc Segment 1 - Event 1

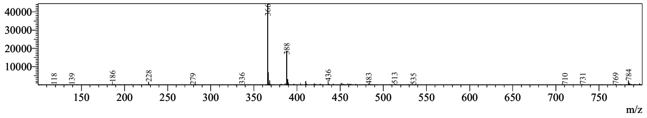

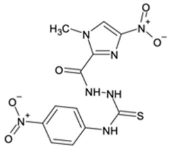

Formula Predictor Report

Printed at 15.06.2024 10:40:13

Formula Predictor Result	C12 H11 N7 O5 S
Mass	366.06104
Error Margin	10 ppm
DBE Range	Not Used
Electron Ions	Both configurations
HC Ratio	Not Used
Nitrogen Rule	Used

#	Score	Pred. (M)	Pred. m/z	Meas. m/z	Diff. (mDa)	Formulae (M)	Ion	Diff. (ppm)	Iso Score	DBE
2	91.52	365.05424	366.06151	366.06104	-0.47	C12 H11 N7 O5 S	[M+H]+	-1.295	90.58	11.0

Event#: 1 MS(E+) Ret. Time : [2.550->2.685]-[0.975->1.785] Scan# : [511->538]-[196->358] 7.38e3

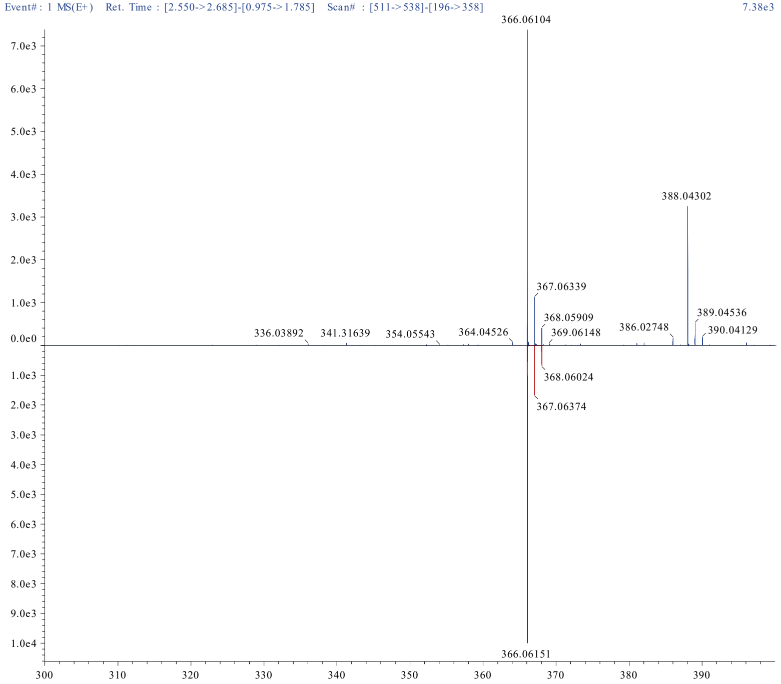

ts([C12 H11 N7 O5 S+H]+) 1.00e4

C:\LabSolutions\Data\DATA\KD-17-_i-MeOH_70-15m-03-(100-800)__14-6-2024_1.lcd

Figure S45. The MS of compound **18**.

SHIMADZU LabSolutions

Analysis Report

Sample Name	:
Sample ID	:
Data Filename	:
Method Filename	: i-MeOH_70-15m-03-(100-800)_.lcm
Batch Filename	: 14-6-2024.lcb
Vial #	: 1-7
Injection Volume	: 0.1 uL
Date Acquired	: 6/14/2024 6:53:35 PM
Date Processed	: 6/14/2024 7:08:36 PM
Sample Type	: Unknown
Acquired by	: System Administrator
Processed by	: System Administrator

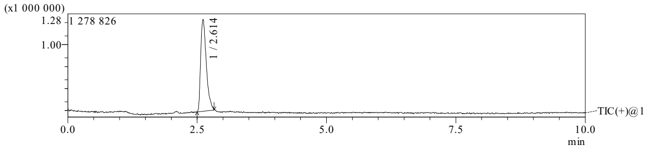

MASS Peak Table TIC

Peak#	Ret. Time	m/z	Area%
1	2.614	TIC	100.000
Total			100.000

MS Spectrum

Line#:1 R.Time:----(Scan#:----)
MassPeaks:606
Spectrum Mode:Averaged 2.605-2.615(522-524) Base Peak:274(319841)
BG Mode:Calc Segment 1 - Event 1

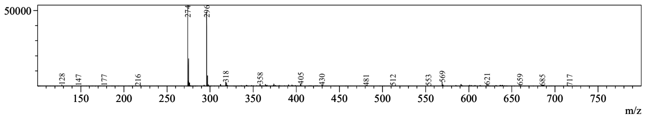

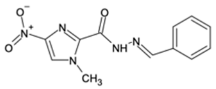

Formula Predictor Report

Printed at 15.06.2024 10:44:33

Formula Predictor Result	C12 H11 N5 O3
Mass	274.09288
Error Margin	10 ppm
DBE Range	Not Used
Electron Ions	Both configurations
HC Ratio	Not Used
Nitrogen Rule	Used

#	Score	Pred. (M)	Pred. m/z	Meas. m/z	Diff. (mDa)	Formulae (M)	Ion	Diff. (ppm)	Iso Score	DBE
2	96.91	273.08619	274.09347	274.09288	-0.59	C12 H11 N5 O3	[M+H]+	-2.137	96.57	10.0

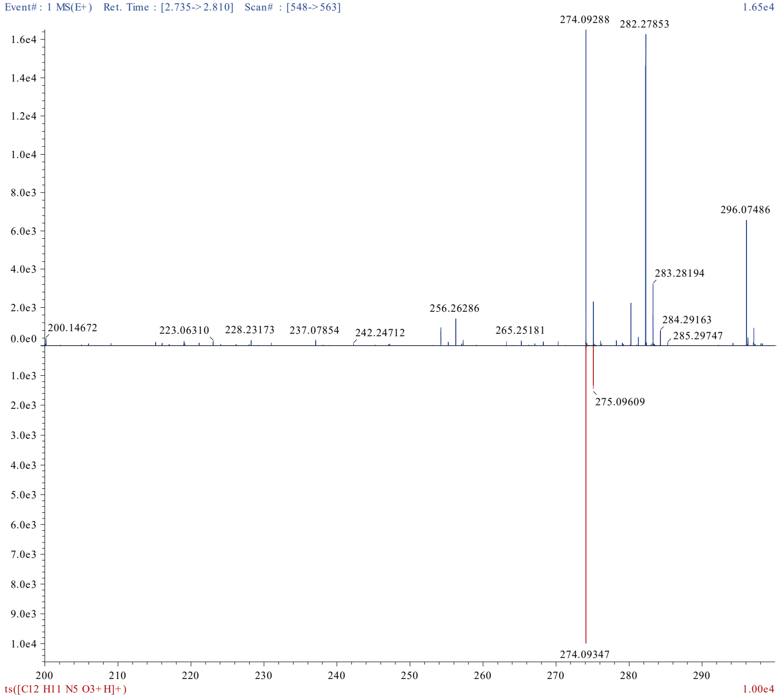

C:\LabSolutions\Data\DATA\KD-19-_i-MeOH_70-15m-03-(100-800)__14-6-2024_1.lcd

Figure S46. The MS of compound **19**.

SHIMADZU LabSolutions

Analysis Report

Sample Name :
Sample ID :
Data Filename :
Method Filename : i-MeOH_70-15m-03-(100-800)_.lcm
Batch Filename : 14-6-2024.lcb
Vial # : 1-18 Sample Type : Unknown
Injection Volume : 0.1 uL
Date Acquired : 6/15/2024 12:00:08 AM Acquired by : System Administrator
Date Processed : 6/15/2024 12:15:09 AM Processed by : System Administrator

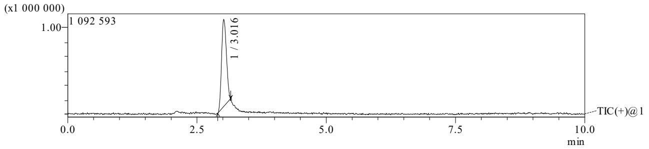

MASS Peak Table TIC

Peak#	Ret. Time	m/z	Area%
1	3.016	TIC	100.000
Total			100.000

MS Spectrum

Line#:1 R.Time:----(Scan#:----)
MassPeaks:552
Spectrum Mode:Averaged 3.010-3.020(603-605) Base Peak:308(218712)
BG Mode:Calc Segment 1 - Event 1

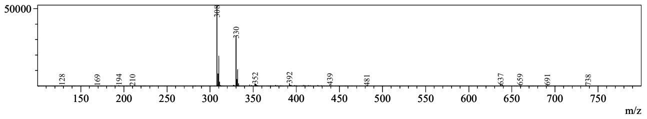

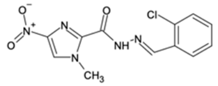

Formula Predictor Report

Printed at 15.06.2024 11:02:55

Formula Predictor Result	C12 H10 N5 O3 Cl
Mass	308.05423
Error Margin	10 ppm
DBE Range	Not Used
Electron Ions	Both configurations
HC Ratio	Not Used
Nitrogen Rule	Used

#	Score	Pred. (M)	Pred. m/z	Meas. m/z	Diff. (mDa)	Formulae (M)	Ion	Diff. (ppm)	Iso Score	DBE
1	98.70	307.04722	308.05449	308.05423	-0.26	C12 H10 N5 O3 Cl	[M+H]+	-0.855	98.55	10.0

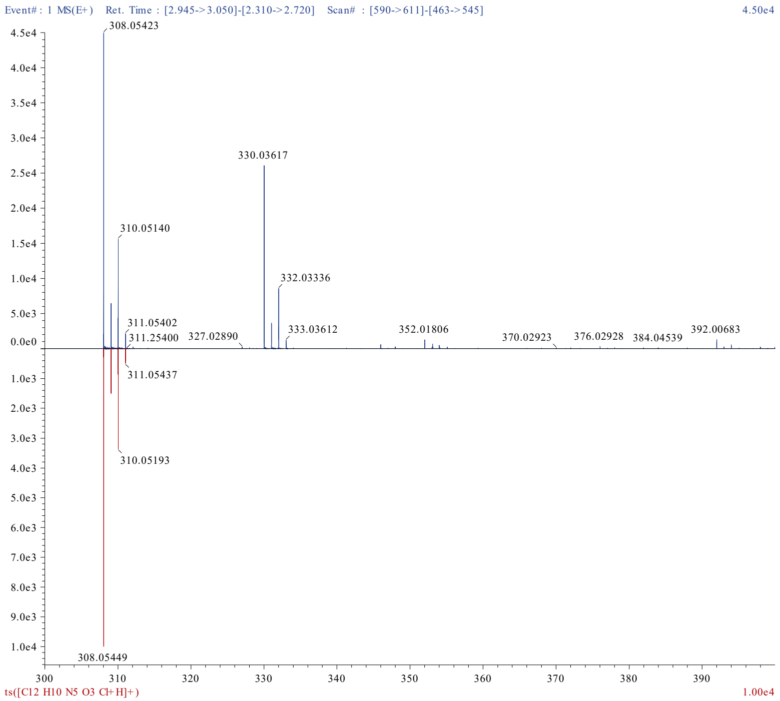

C:\LabSolutions\Data\DATA\KD-36-_i-MeOH_70-15m-03-(100-800)__14-6-2024_4.lcd

Figure S47. The MS of compound **20**.

SHIMADZU LabSolutions

Analysis Report

Sample Name	:
Sample ID	:
Data Filename	:
Method Filename	: i-MeOH_70-15m-03-(100-800)_.lcm
Batch Filename	: 14-6-2024.lcb
Vial #	: 1-19
Injection Volume	: 0.1 uL
Date Acquired	: 6/15/2024 12:15:35 AM
Date Processed	: 6/15/2024 12:30:36 AM

Sample Type	: Unknown
Acquired by	: System Administrator
Processed by	: System Administrator

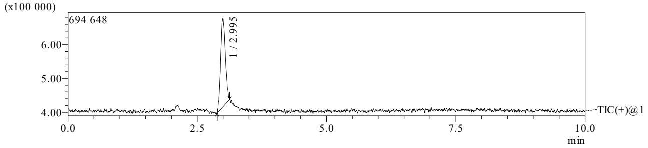

MASS Peak Table TIC

Peak#	Ret. Time	m/z	Area%
1	2.995	TIC	100.000
Total			100.000

MS Spectrum

Line#:1 R.Time:----(Scan#:----)
MassPeaks:353
Spectrum Mode:Averaged 2.990-3.000(599-601) Base Peak:308(114059)
BG Mode:Calc Segment 1 - Event 1

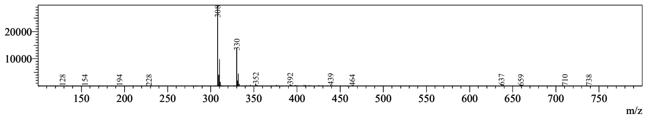

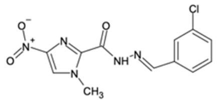

Formula Predictor Report

Printed at 15.06.2024 11:04:17

Formula Predictor Result	C12 H10 N5 O3 Cl
Mass	308.05430
Error Margin	10 ppm
DBE Range	Not Used
Electron Ions	Both configurations
HC Ratio	Not Used
Nitrogen Rule	Used

#	Score	Pred. (M)	Pred. m/z	Meas. m/z	Diff. (mDa)	Formulae (M)	Ion	Diff. (ppm)	Iso Score	DBE
1	99.33	307.04722	308.05449	308.05430	-0.19	C12 H10 N5 O3 Cl	[M+H]+	-0.628	99.25	10.0

Event#: 1 MS(E+) Ret. Time : [2.895->3.035]-[1.530->2.265] Scan# : [580->608]-[307->454] 1.98e4

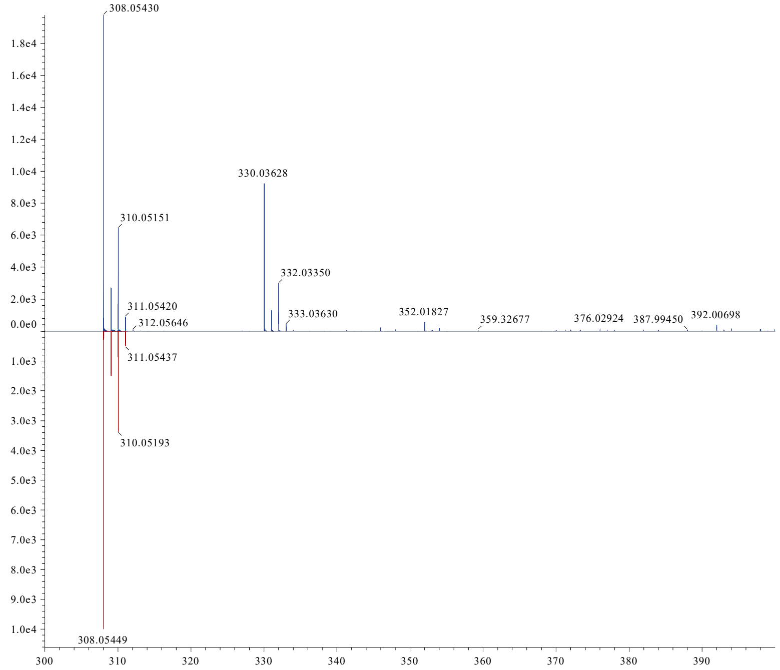

ts([C12 H10 N5 O3 Cl+H]+) 1.00e4

C:\LabSolutions\Data\DATA\KD-37-_i-MeOH_70-15m-03-(100-800)__14-6-2024_5.lcd

Figure S48. The MS of compound **21**.

Analysis Report

Sample Name :
Sample ID :
Data Filename :
Method Filename : i-MeOH_70-15m-03-(100-800)_.lcm
Batch Filename : 14-6-2024.lcb
Vial # : 1-20
Sample Type : Unknown
Injection Volume : 0.1 uL
Date Acquired : 6/15/2024 12:31:02 AM
Acquired by : System Administrator
Date Processed : 6/15/2024 12:46:02 AM
Processed by : System Administrator

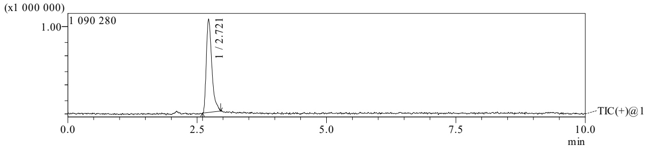

MASS Peak Table TIC

Peak#	Ret. Time	m/z	Area%
1	2.721	TIC	100.000
Total			100.000

MS Spectrum

Line#:1 R.Time:----(Scan#:----)
MassPeaks:497
Spectrum Mode:Averaged 2.715-2.725(544-546) Base Peak:292(258975)
BG Mode:Calc Segment 1 - Event 1

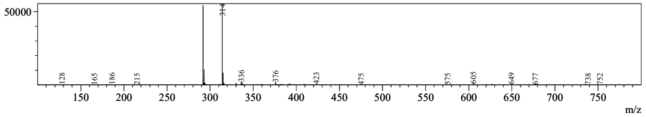

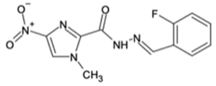

Formula Predictor Report

Printed at 15.06.2024 11:06:10

Formula Predictor Result	C12 H10 N5 O3 F
Mass	292.08392
Error Margin	10 ppm
DBE Range	Not Used
Electron Ions	Both configurations
HC Ratio	Not Used
Nitrogen Rule	Used

#	Score	Pred. (M)	Pred. m/z	Meas. m/z	Diff. (mDa)	Formulae (M)	Ion	Diff. (ppm)	Iso Score	DBE
1	100.00	291.07677	292.08404	292.08392	-0.12	C12 H10 N5 O3 F	[M+H]+	-0.424	100.00	10.0

Event# : 1 MS(E+) Ret. Time : [2.720->2.810]-[2.010->2.505] Scan# : [545->563]-[403->502] 4.68e4

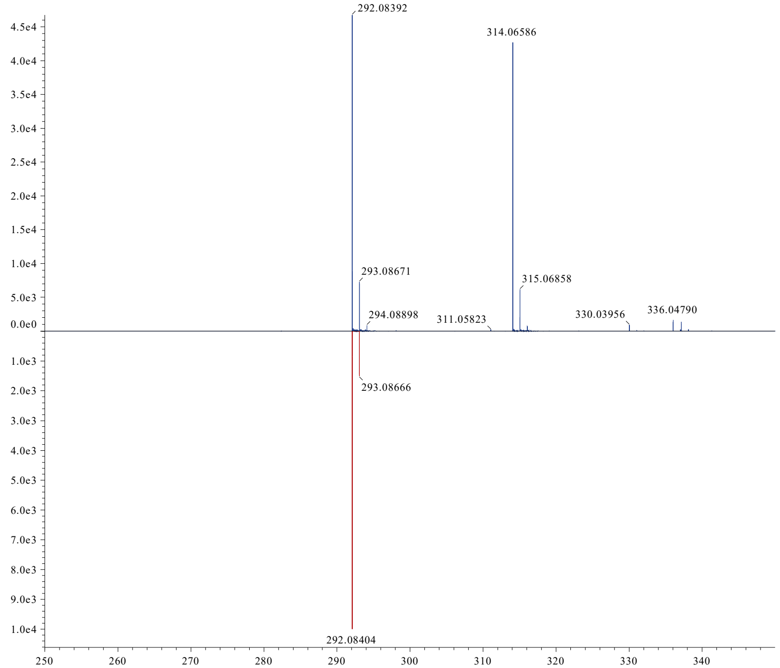

ts([C12 H10 N5 O3 F+H]+) 1.00e4

C:\LabSolutions\Data\DATA\KD-39-_i-MeOH_70-15m-03-(100-800)__14-6-2024_6.lcd

Figure S49. The MS of compound **22**.

SHIMADZU LabSolutions

Analysis Report

Sample Name	:
Sample ID	:
Data Filename	:
Method Filename	: i-MeOH_70-15m-03-(100-800)_.lcm
Batch Filename	: 14-6-2024.lcb
Vial #	: 1-9
Injection Volume	: 0.1 uL
Date Acquired	: 6/14/2024 9:41:17 PM
Date Processed	: 6/14/2024 9:56:18 PM
Sample Type	: Unknown
Acquired by	: System Administrator
Processed by	: System Administrator

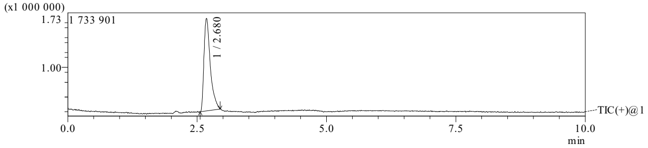

MASS Peak Table TIC

Peak#	Ret. Time	m/z	Area%
1	2.680	TIC	100.000
Total			100.000

MS Spectrum

Line#:1 R.Time:----(Scan#:----)
MassPeaks:702
Spectrum Mode:Averaged 2.670-2.680(535-537) Base Peak:304(426564)
BG Mode:Calc Segment 1 - Event 1

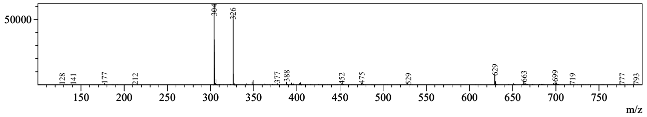

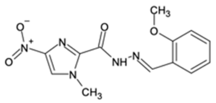

Formula Predictor Report

Printed at 15.06.2024 10:48:08

Formula Predictor Result	C13 H13 N5 O4
Mass	304.10331
Error Margin	10 ppm
DBE Range	Not Used
Electron Ions	Both configurations
HC Ratio	Not Used
Nitrogen Rule	Used

#	Score	Pred. (M)	Pred. m/z	Meas. m/z	Diff. (mDa)	Formulae (M)	Ion	Diff. (ppm)	Iso Score	DBE
2	93.11	303.09675	304.10403	304.10331	-0.72	C13 H13 N5 O4	[M+H]+	-2.369	92.34	10.0

Event# : 1 MS(E+) Ret. Time : [2.735->2.855]-[1.420->2.325] Scan# : [548->572]-[285->466]

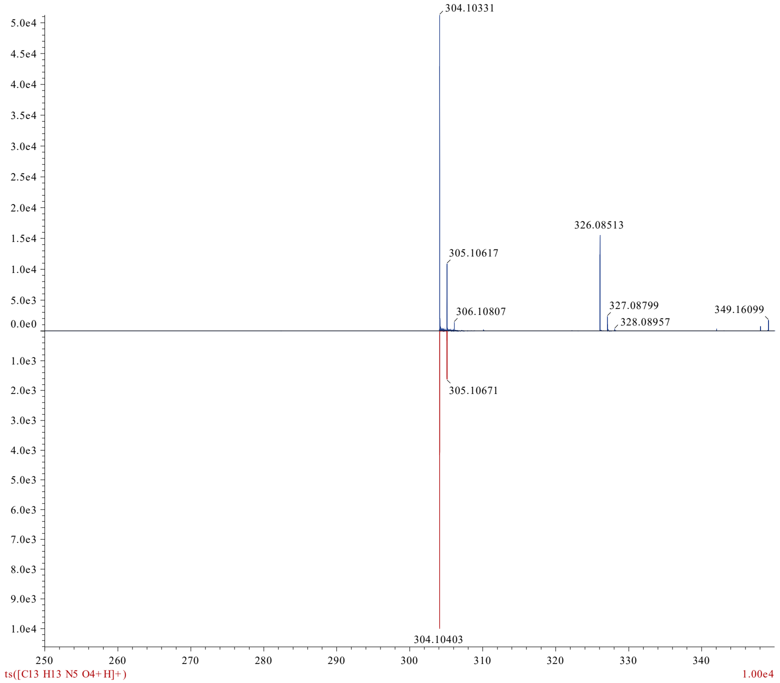

C:\LabSolutions\Data\DATA\KD-21-_i-MeOH_70-15m-03-(100-800)__14-6-2024_1.lcd

Figure S50. The MS of compound **23**.

SHIMADZU LabSolutions

Analysis Report

Sample Name	:		
Sample ID	:		
Data Filename	:		
Method Filename	: i-MeOH_70-15m-03-(100-800)_.lcm		
Batch Filename	: 14-6-2024.lcb		
Vial #	: 1-10	Sample Type	: Unknown
Injection Volume	: 0.1 uL		
Date Acquired	: 6/14/2024 9:56:43 PM	Acquired by	: System Administrator
Date Processed	: 6/14/2024 10:11:44 PM	Processed by	: System Administrator

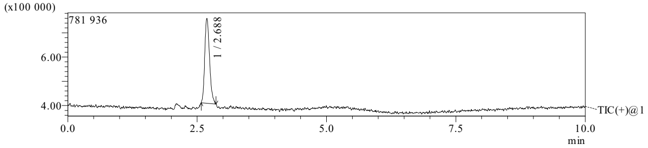

MASS Peak Table TIC

Peak#	Ret. Time	m/z	Area%
1	2.688	TIC	100.000
Total			100.000

MS Spectrum

Line#:1 R.Time:----(Scan#:----)
MassPeaks:356
Spectrum Mode:Averaged 2.680-2.690(537-539) Base Peak:304(185890)
BG Mode:Calc Segment 1 - Event 1

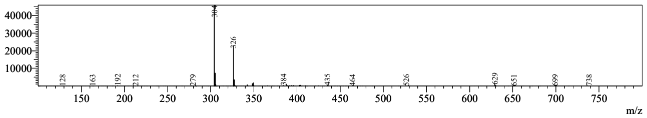

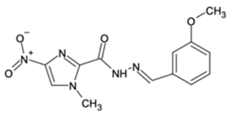

Formula Predictor Report

Printed at 15.06.2024 10:49:27

Formula Predictor Result	C13 H13 N5 O4
Mass	304.10343
Error Margin	10 ppm
DBE Range	Not Used
Electron Ions	Both configurations
HC Ratio	Not Used
Nitrogen Rule	Used

#	Score	Pred. (M)	Pred. m/z	Meas. m/z	Diff. (mDa)	Formulae (M)	Ion	Diff. (ppm)	Iso Score	DBE
2	97.33	303.09675	304.10403	304.10343	-0.60	C13 H13 N5 O4	[M+H]+	-1.974	97.04	10.0

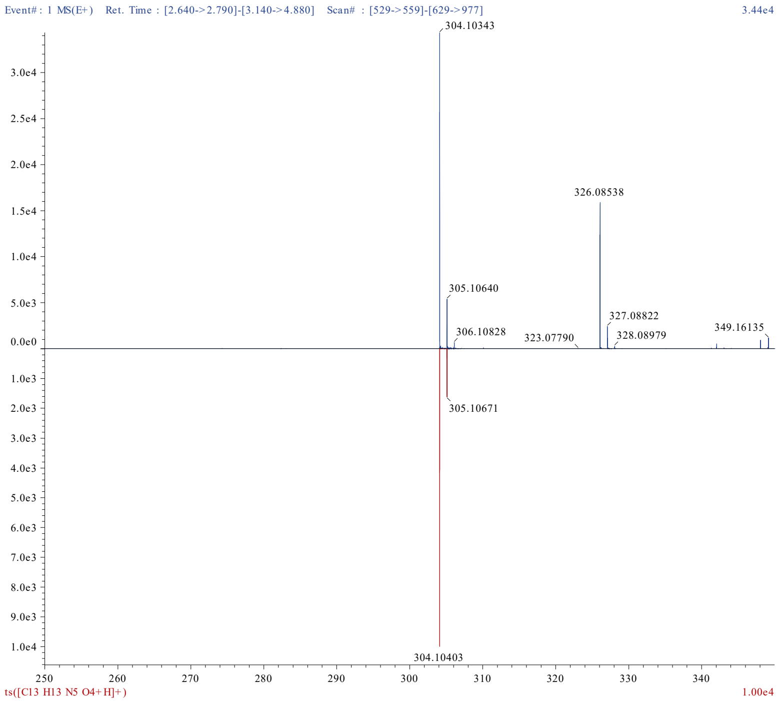

C:\LabSolutions\Data\DATA\KD-22-_i-MeOH_70-15m-03-(100-800)__14-6-2024_2.lcd

Figure S51. The MS of compound **24**.

SHIMADZU LabSolutions

Analysis Report

Sample Name :
Sample ID :
Data Filename :
Method Filename : i-MeOH_70-15m-03-(100-800)_.lcm
Batch Filename : 14-6-2024.lcb
Vial # : 1-14 Sample Type : Unknown
Injection Volume : 0.1 uL
Date Acquired : 6/14/2024 10:58:27 PM Acquired by : System Administrator
Date Processed : 6/14/2024 11:13:29 PM Processed by : System Administrator

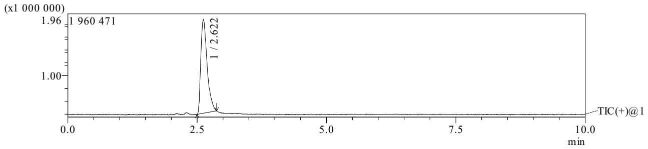

MASS Peak Table TIC

Peak#	Ret. Time	m/z	Area%
1	2.622	TIC	100.000
Total			100.000

MS Spectrum

Line#:1 R.Time:----(Scan#:----)
MassPeaks:783
Spectrum Mode:Averaged 2.615-2.625(524-526) Base Peak:304(435779)
BG Mode:Calc Segment 1 - Event 1

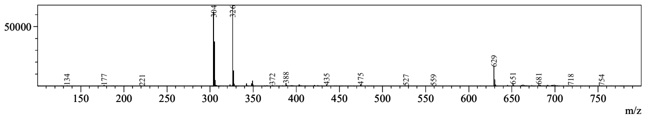

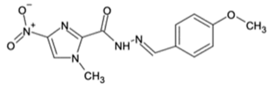

Formula Predictor Report

Printed at 15.06.2024 10:57:48

Formula Predictor Result	C13 H13 N5 O4
Mass	304.10360
Error Margin	10 ppm
DBE Range	Not Used
Electron Ions	Both configurations
HC Ratio	Not Used
Nitrogen Rule	Used

#	Score	Pred. (M)	Pred. m/z	Meas. m/z	Diff. (mDa)	Formulae (M)	Ion	Diff. (ppm)	Iso Score	DBE
2	98.94	303.09675	304.10403	304.10360	-0.43	C13 H13 N5 O4	[M+H]+	-1.415	98.83	10.0

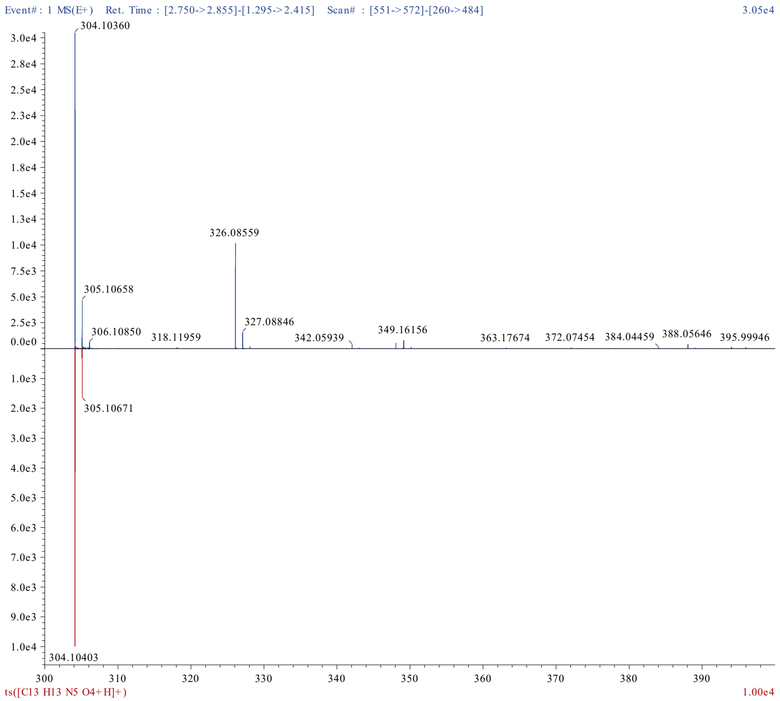

C:\LabSolutions\Data\DATA\KD-26-_i-MeOH_70-15m-03-(100-800)__14-6-2024_6.lcd

Figure S52. The MS of compound **25**.

SHIMADZU LabSolutions

Analysis Report

Sample Name :
Sample ID :
Data Filename :
Method Filename : i-MeOH_70-15m-03-(100-800)_.lcm
Batch Filename : 14-6-2024.lcb
Vial # : 1-21 Sample Type : Unknown
Injection Volume : 0.1 uL
Date Acquired : 6/15/2024 12:46:27 AM Acquired by : System Administrator
Date Processed : 6/15/2024 1:01:28 AM Processed by : System Administrator

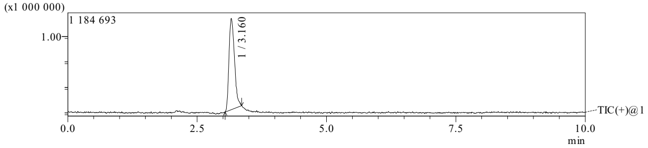

MASS Peak Table TIC

Peak#	Ret. Time	m/z	Area%
1	3.160	TIC	100.000
Total			100.000

MS Spectrum

Line#:1 R.Time:----(Scan#:----)
MassPeaks:499
Spectrum Mode:Averaged 3.155-3.165(632-634) Base Peak:342(369757)
BG Mode:Calc Segment 1 - Event 1

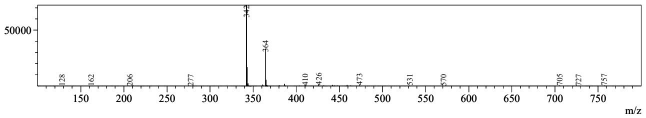

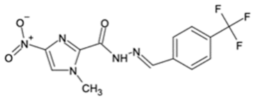

Formula Predictor Report

Printed at 15.06.2024 11:07:42

Formula Predictor Result	C13 H10 N5 O3 F3
Mass	342.08072
Error Margin	10 ppm
DBE Range	Not Used
Electron Ions	Both configurations
HC Ratio	Not Used
Nitrogen Rule	Used

#	Score	Pred. (M)	Pred. m/z	Meas. m/z	Diff. (mDa)	Formulae (M)	Ion	Diff. (ppm)	Iso Score	DBE
1	99.99	341.07357	342.08085	342.08072	-0.13	C13 H10 N5 O3 F3	[M+H]+	-0.380	99.99	10.0

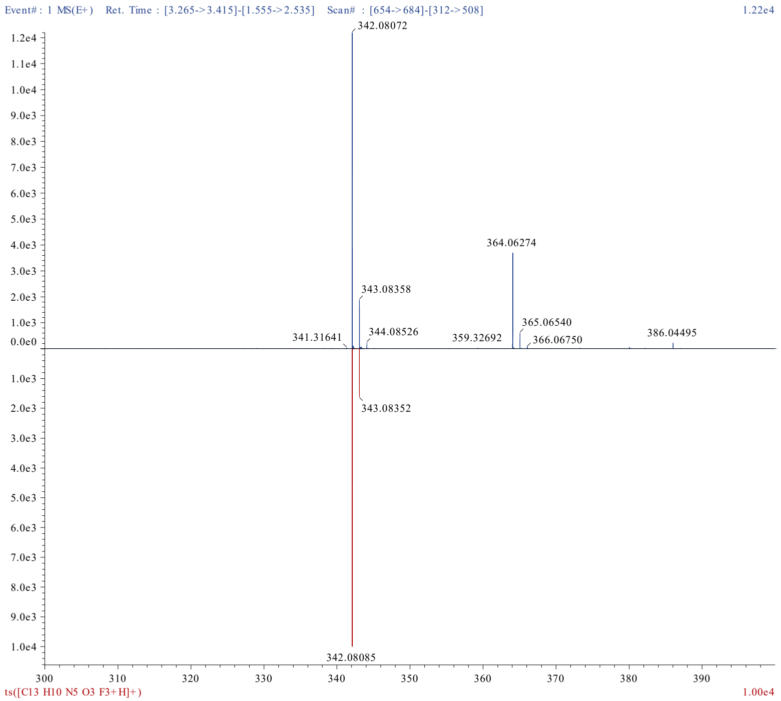

C:\LabSolutions\Data\DATA\KD-42-_i-MeOH_70-15m-03-(100-800)__14-6-2024_7.lcd

Figure S53. The MS of compound **26**.

Analysis Report

Sample Name	:
Sample ID	:
Data Filename	:
Method Filename	: i-MeOH_70-15m-03-(100-800)_.lcm
Batch Filename	: 14-6-2024.lcb
Vial #	: 1-8
Sample Type	: Unknown
Injection Volume	: 0.1 uL
Date Acquired	: 6/14/2024 7:09:01 PM
Acquired by	: System Administrator
Date Processed	: 6/14/2024 7:12:20 PM
Processed by	: System Administrator

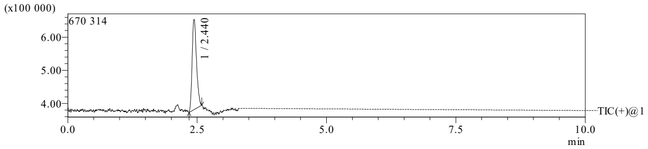

MASS Peak Table TIC

Peak#	Ret. Time	m/z	Area%
1	2.440	TIC	100.000
Total			100.000

MS Spectrum

Line#:1 R.Time:----(Scan#:----)
MassPeaks:335
Spectrum Mode:Averaged 2.435-2.445(488-490) Base Peak:290(153162)
BG Mode:Calc Segment 1 - Event 1

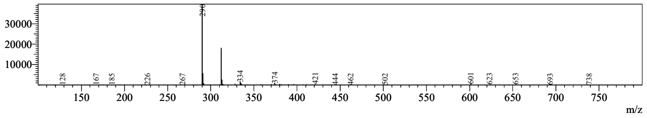

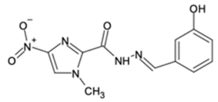

Formula Predictor Report

Printed at 15.06.2024 10:46:36

Formula Predictor Result	C12 H11 N5 O4
Mass	290.08798
Error Margin	10 ppm
DBE Range	Not Used
Electron Ions	Both configurations
HC Ratio	Not Used
Nitrogen Rule	Used

#	Score	Pred. (M)	Pred. m/z	Meas. m/z	Diff. (mDa)	Formulae (M)	Ion	Diff. (ppm)	Iso Score	DBE
2	99.12	289.08110	290.08838	290.08798	-0.40	C12 H11 N5 O4	[M+H]+	-1.380	99.02	10.0

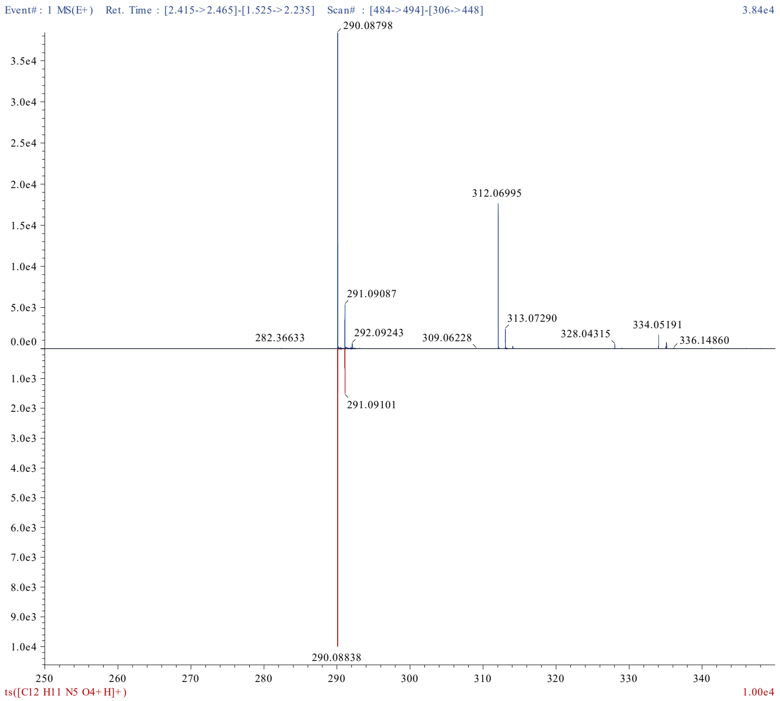

C:\LabSolutions\Data\DATA\KD-20-_i-MeOH_70-15m-03-(100-800)__14-6-2024_2.lcd

Figure S54. The MS of compound **27**.

SHIMADZU LabSolutions

Analysis Report

Sample Name	:
Sample ID	:
Data Filename	:
Method Filename	: i-MeOH_70-15m-03-(100-800)_.lcm
Batch Filename	: 14-6-2024.lcb
Vial #	: 1-12
Sample Type	: Unknown
Injection Volume	: 0.1 uL
Date Acquired	: 6/14/2024 10:27:35 PM
Acquired by	: System Administrator
Date Processed	: 6/14/2024 10:42:37 PM
Processed by	: System Administrator

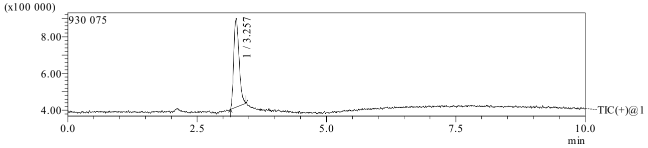

MASS Peak Table TIC

Peak#	Ret. Time	m/z	Area%
1	3.257	TIC	100.000
Total			100.000

MS Spectrum

Line#:1 R.Time:----(Scan#:----)
MassPeaks:467
Spectrum Mode:Averaged 3.250-3.260(651-653) Base Peak:326(177609)
BG Mode:Calc Segment 1 - Event 1

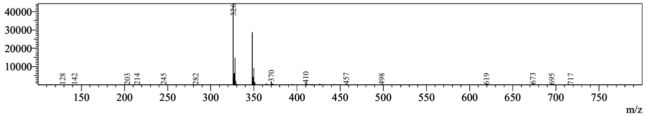

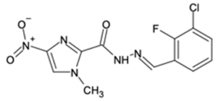

Formula Predictor Report

Printed at 15.06.2024 10:53:22

Formula Predictor Result	C12 H9 N5 O3 F Cl
Mass	326.04462
Error Margin	10 ppm
DBE Range	Not Used
Electron Ions	Both configurations
HC Ratio	Not Used
Nitrogen Rule	Used

#	Score	Pred. (M)	Pred. m/z	Meas. m/z	Diff. (mDa)	Formulae (M)	Ion	Diff. (ppm)	Iso Score	DBE
2	87.59	325.03780	326.04507	326.04462	-0.45	C12 H9 N5 O3 F Cl	[M+H]+	-1.385	97.33	10.0

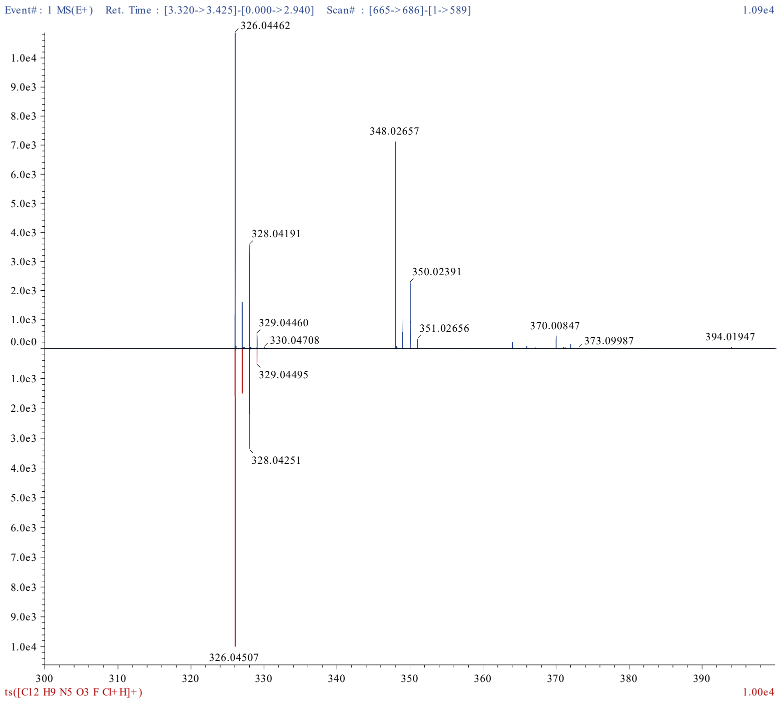

C:\LabSolutions\Data\DATA\KD-24-_i-MeOH_70-15m-03-(100-800)__14-6-2024_4.lcd

Figure S55. The MS of compound **28**.

Analysis Report

SHIMADZU LabSolutions

Sample Name :
Sample ID :
Data Filename :
Method Filename : i-MeOH_70-15m-03-(100-800)_.lcm
Batch Filename : 14-6-2024.lcb
Vial # : 1-11 Sample Type : Unknown
Injection Volume : 0.1 uL
Date Acquired : 6/14/2024 10:12:10 PM Acquired by : System Administrator
Date Processed : 6/14/2024 10:27:11 PM Processed by : System Administrator

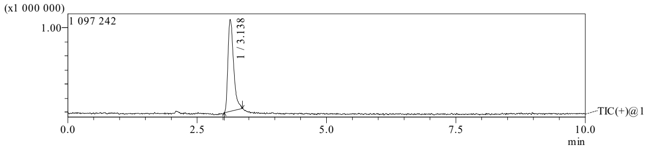

MASS Peak Table TIC

Peak#	Ret. Time	m/z	Area%
1	3.138	TIC	100.000
Total			100.000

MS Spectrum

Line#:1 R.Time:----(Scan#:----)
MassPeaks:499
Spectrum Mode:Averaged 3.130-3.140(627-629) Base Peak:338(274179)
BG Mode:Calc Segment 1 - Event 1

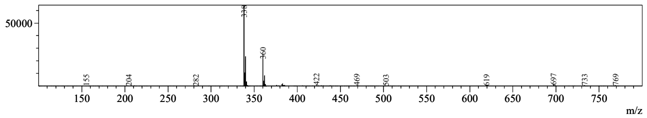

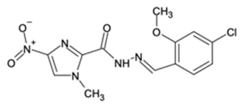

Formula Predictor Report

Printed at 15.06.2024 10:51:36

Formula Predictor Result	C13 H12 N5 O4 Cl
Mass	338.06457
Error Margin	10 ppm
DBE Range	Not Used
Electron Ions	Both configurations
HC Ratio	Not Used
Nitrogen Rule	Used

#	Score	Pred. (M)	Pred. m/z	Meas. m/z	Diff. (mDa)	Formulae (M)	Ion	Diff. (ppm)	Iso Score	DBE
2	85.91	337.05778	338.06506	338.06457	-0.49	C13 H12 N5 O4 Cl	[M+H]+	-1.444	95.46	10.0

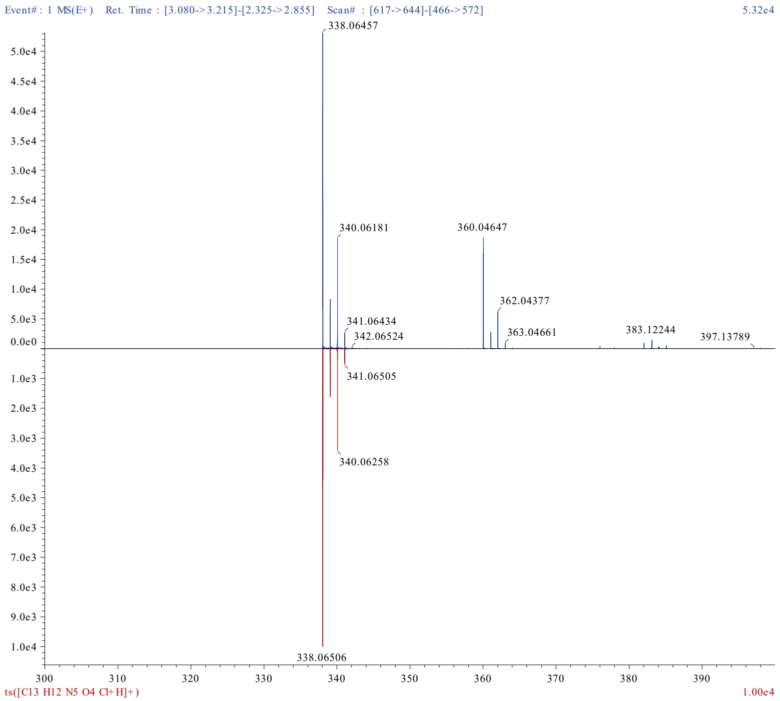

Figure S56. The MS of compound **29**.

SHIMADZU LabSolutions

Analysis Report

Sample Name	:
Sample ID	:
Data Filename	:
Method Filename	: i-MeOH_70-15m-03-(100-800)_.lcm
Batch Filename	: 14-6-2024.lcb
Vial #	: 1-13
Injection Volume	: 0.1 uL
Date Acquired	: 6/14/2024 10:43:02 PM
Date Processed	: 6/14/2024 10:58:02 PM
Sample Type	: Unknown
Acquired by	: System Administrator
Processed by	: System Administrator

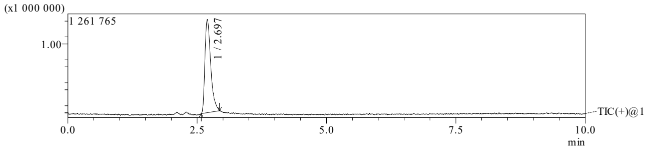

MASS Peak Table TIC

Peak#	Ret. Time	m/z	Area%
1	2.697	TIC	100.000
Total			100.000

MS Spectrum

Line#:1 R.Time:----(Scan#:----)
MassPeaks:505
Spectrum Mode:Averaged 2.690-2.700(539-541) Base Peak:320(403196)
BG Mode:Calc Segment 1 - Event 1

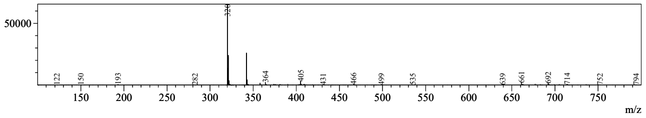

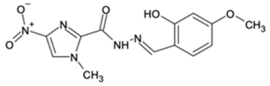

Formula Predictor Report

Printed at 15.06.2024 10:54:45

Formula Predictor Result	C13 H13 N5 O5
Mass	320.09884
Error Margin	10 ppm
DBE Range	Not Used
Electron Ions	Both configurations
HC Ratio	Not Used
Nitrogen Rule	Used

#	Score	Pred. (M)	Pred. m/z	Meas. m/z	Diff. (mDa)	Formulae (M)	Ion	Diff. (ppm)	Iso Score	DBE
3	79.20	319.09167	320.09894	320.09884	-0.10	C13 H13 N5 O5	[M+H]+	-0.328	76.88	10.0

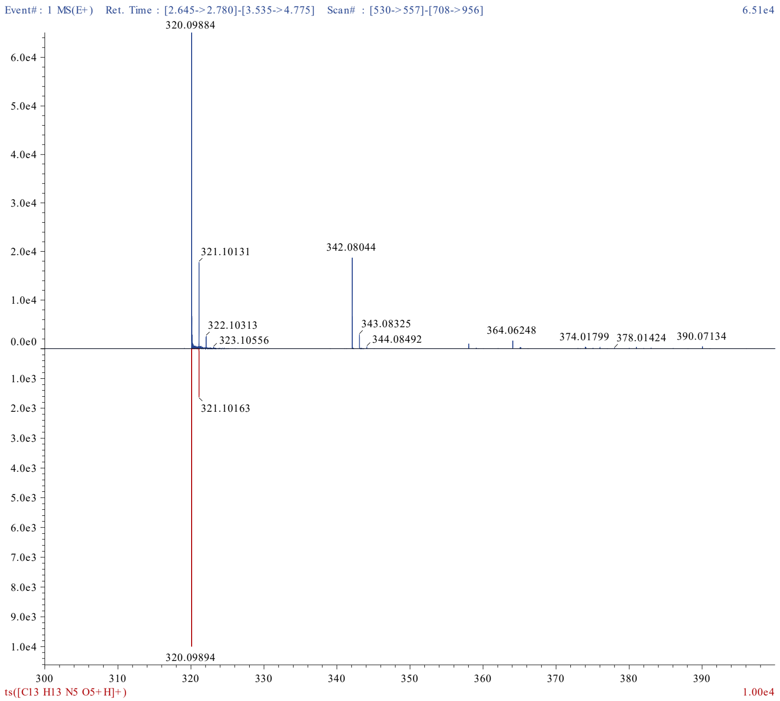

C:\LabSolutions\Data\DATA\KD-25-_i-MeOH_70-15m-03-(100-800)__14-6-2024_5.lcd

Figure S57. The MS of compound **30**.

SHIMADZU LabSolutions

Analysis Report

Sample Name :
Sample ID :
Data Filename :
Method Filename : i-MeOH_70-15m-03-(100-800)_.lcm
Batch Filename : 14-6-2024.lcb
Vial # : 1-15 Sample Type : Unknown
Injection Volume : 0.1 uL
Date Acquired : 6/14/2024 11:13:52 PM Acquired by : System Administrator
Date Processed : 6/14/2024 11:28:54 PM Processed by : System Administrator

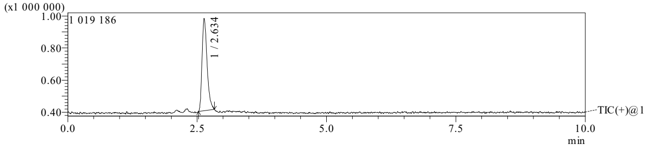

MASS Peak Table TIC

Peak#	Ret. Time	m/z	Area%
1	2.634	TIC	100.000
Total			100.000

MS Spectrum

Line#:1 R.Time:----(Scan#:----)
MassPeaks:467
Spectrum Mode:Averaged 2.625-2.635(526-528) Base Peak:320(271851)
BG Mode:Calc Segment 1 - Event 1

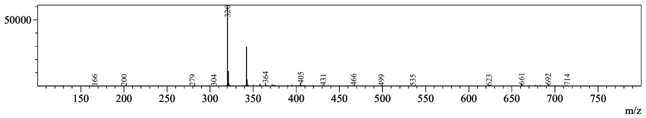

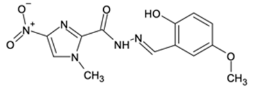

Formula Predictor Report

Printed at 15.06.2024 10:59:01

Formula Predictor Result	C13 H13 N5 O5
Mass	320.09864
Error Margin	10 ppm
DBE Range	Not Used
Electron Ions	Both configurations
HC Ratio	Not Used
Nitrogen Rule	Used

#	Score	Pred. (M)	Pred. m/z	Meas. m/z	Diff. (mDa)	Formulae (M)	Ion	Diff. (ppm)	Iso Score	DBE
2	99.29	319.09167	320.09894	320.09864	-0.30	C13 H13 N5 O5	[M+H]+	-0.953	99.21	10.0

Event#: 1 MS(E+) Ret. Time : [2.580->2.705]-[1.115->1.510] Scan# : [517->542]-[224->303] 5.33e4

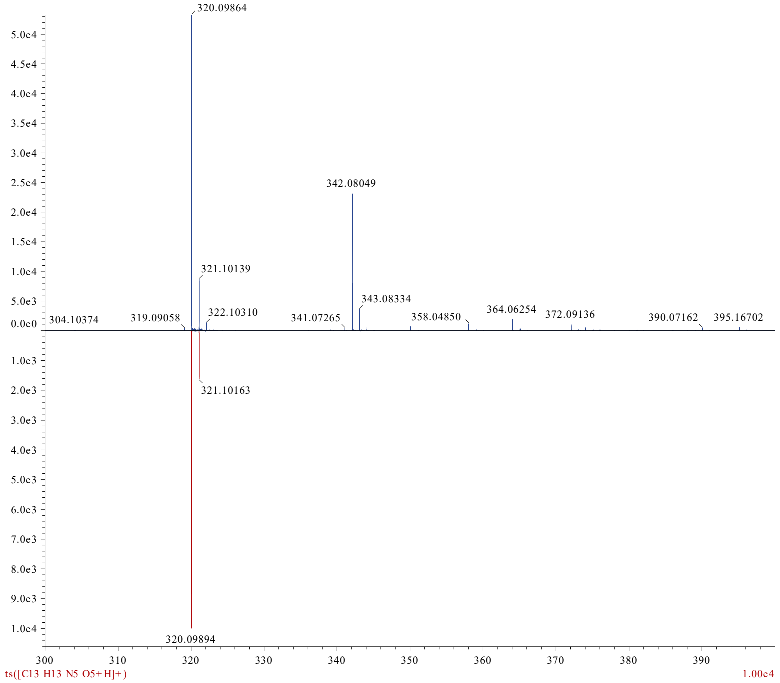

C:\LabSolutions\Data\DATA\KD-27-_i-MeOH_70-15m-03-(100-800)__14-6-2024_1.lcd

Figure S58. The MS of compound **31**.

SHIMADZU LabSolutions

Analysis Report

Sample Name :
Sample ID :
Data Filename :
Method Filename : i-MeOH_70-15m-03-(100-800)_.lcm
Batch Filename : 14-6-2024.lcb
Vial # : 1-16 Sample Type : Unknown
Injection Volume : 0.1 uL
Date Acquired : 6/14/2024 11:29:18 PM Acquired by : System Administrator
Date Processed : 6/14/2024 11:44:20 PM Processed by : System Administrator

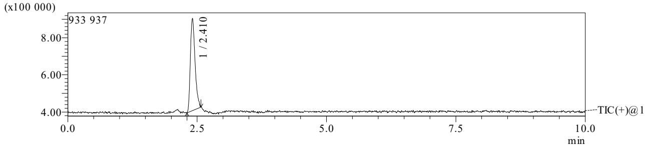

MASS Peak Table TIC

Peak#	Ret. Time	m/z	Area%
1	2.410	TIC	100.000
Total			100.000

MS Spectrum

Line#:1 R.Time:----(Scan#:----)
MassPeaks:445
Spectrum Mode:Averaged 2.400-2.410(481-483) Base Peak:320(253754)
BG Mode:Calc Segment 1 - Event 1

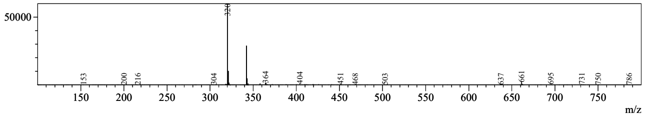

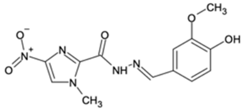

Formula Predictor Report

Printed at 15.06.2024 11:00:32

Formula Predictor Result	C13 H13 N5 O5
Mass	320.09868
Error Margin	10 ppm
DBE Range	Not Used
Electron Ions	Both configurations
HC Ratio	Not Used
Nitrogen Rule	Used

#	Score	Pred. (M)	Pred. m/z	Meas. m/z	Diff. (mDa)	Formulae (M)	Ion	Diff. (ppm)	Iso Score	DBE
2	99.60	319.09167	320.09894	320.09868	-0.26	C13 H13 N5 O5	[M+H]+	-0.828	99.55	10.0

Event#: 1 MS(E+) Ret. Time : [2.370->2.505]-[0.870->1.260] Scan# : [475->502]-[175->253] 4.66e4

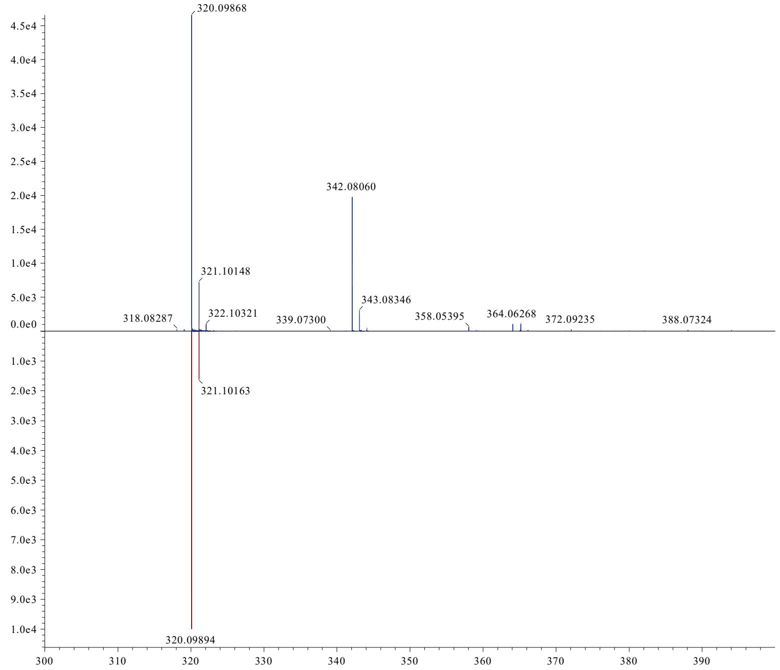

ts([C13 H13 N5 O5+H]+) 1.00e4

C:\LabSolutions\Data\DATA\KD-28-_i-MeOH_70-15m-03-(100-800)__14-6-2024_2.lcd

Figure S59. The MS of compound **32**.

Analysis Report

Sample Name :
Sample ID :
Data Filename :
Method Filename : i-MeOH_70-15m-03-(100-800)_.lcm
Batch Filename : 14-6-2024.lcb
Vial # : 1-17 Sample Type : Unknown
Injection Volume : 0.1 uL
Date Acquired : 6/14/2024 11:44:44 PM Acquired by : System Administrator
Date Processed : 6/14/2024 11:59:44 PM Processed by : System Administrator

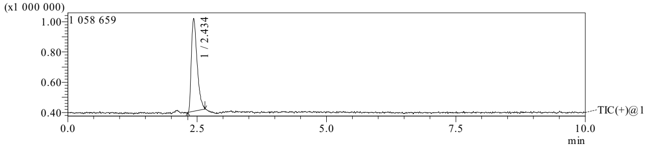

MASS Peak Table TIC

Peak#	Ret. Time	m/z	Area%
1	2.434	TIC	100.000
Total			100.000

MS Spectrum

Line#:1 R.Time:----(Scan#:----)
MassPeaks:382
Spectrum Mode:Averaged 2.425-2.435(486-488) Base Peak:320(371150)
BG Mode:Calc Segment 1 - Event 1

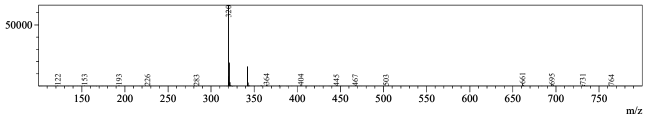

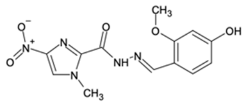

Formula Predictor Report

Printed at 15.06.2024 11:01:38

Formula Predictor Result	C13 H13 N5 O5
Mass	320.09884
Error Margin	10 ppm
DBE Range	Not Used
Electron Ions	Both configurations
HC Ratio	Not Used
Nitrogen Rule	Used

#	Score	Pred. (M)	Pred. m/z	Meas. m/z	Diff. (mDa)	Formulae (M)	Ion	Diff. (ppm)	Iso Score	DBE
2	96.19	319.09167	320.09894	320.09884	-0.10	C13 H13 N5 O5	[M+H]+	-0.328	95.76	10.0

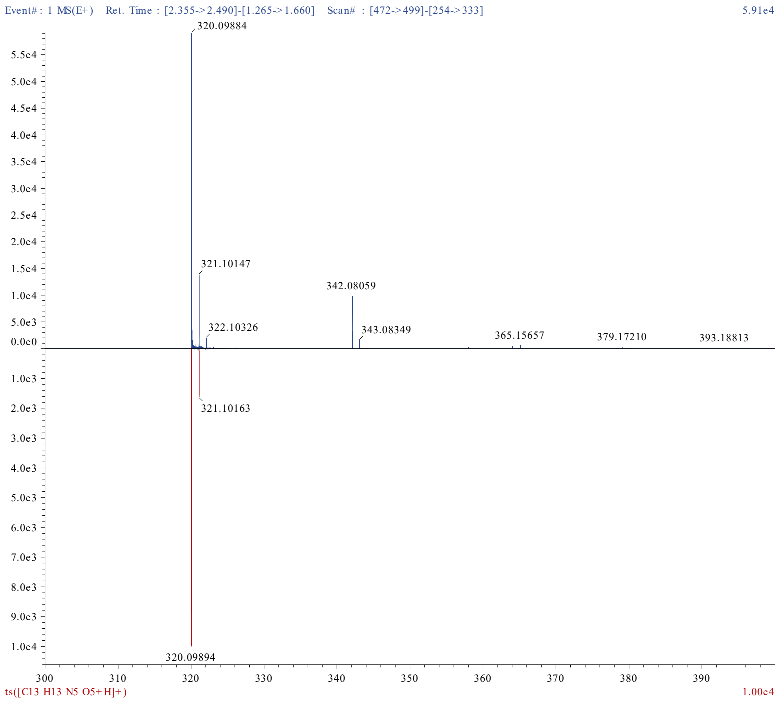

C:\LabSolutions\Data\DATA\KD-31-_i-MeOH_70-15m-03-(100-800)__14-6-2024_3.lcd

Figure S60. The MS of compound **33**.

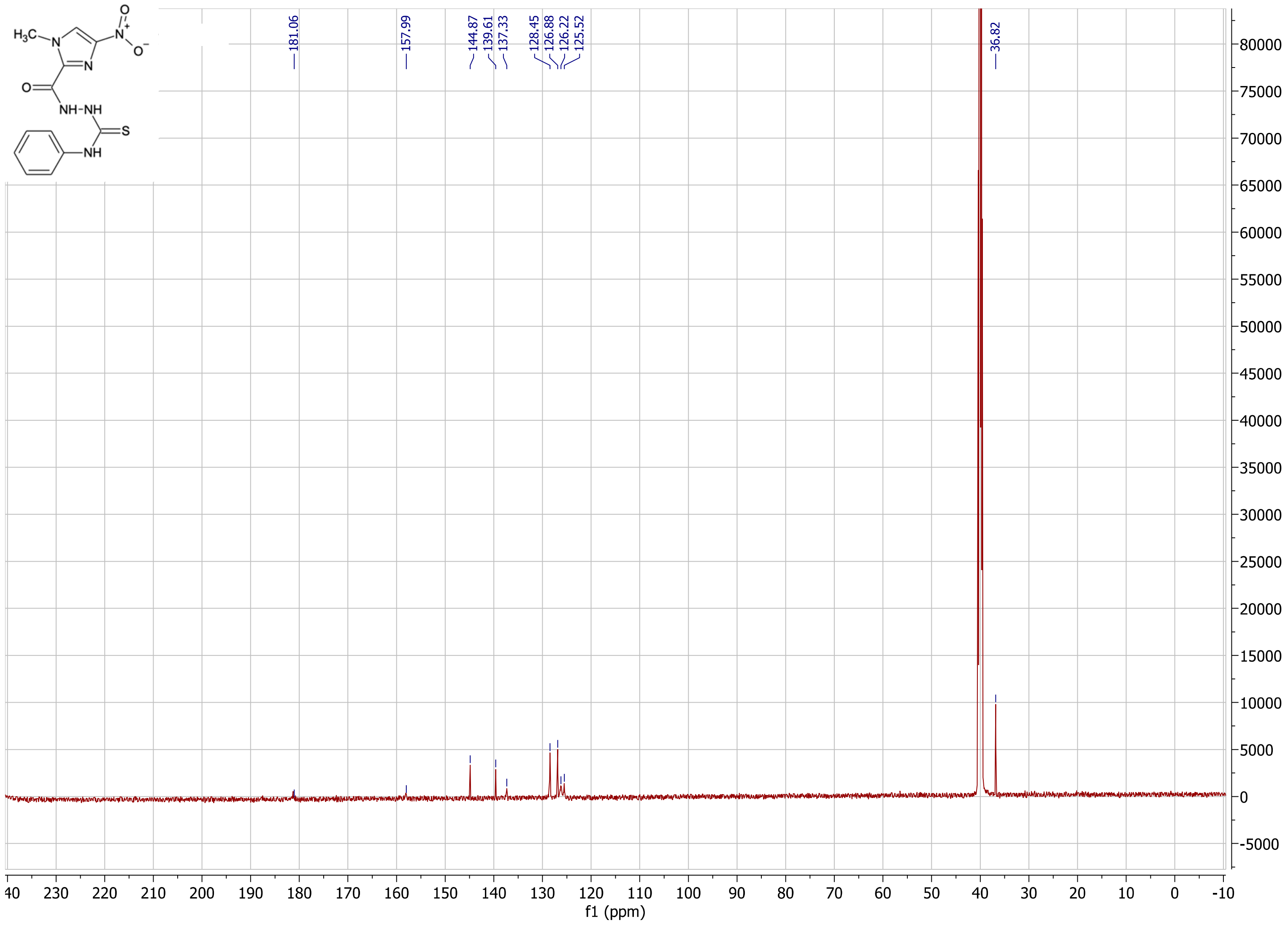

Figure S61. The ^{13}C NMR of compound **2.**

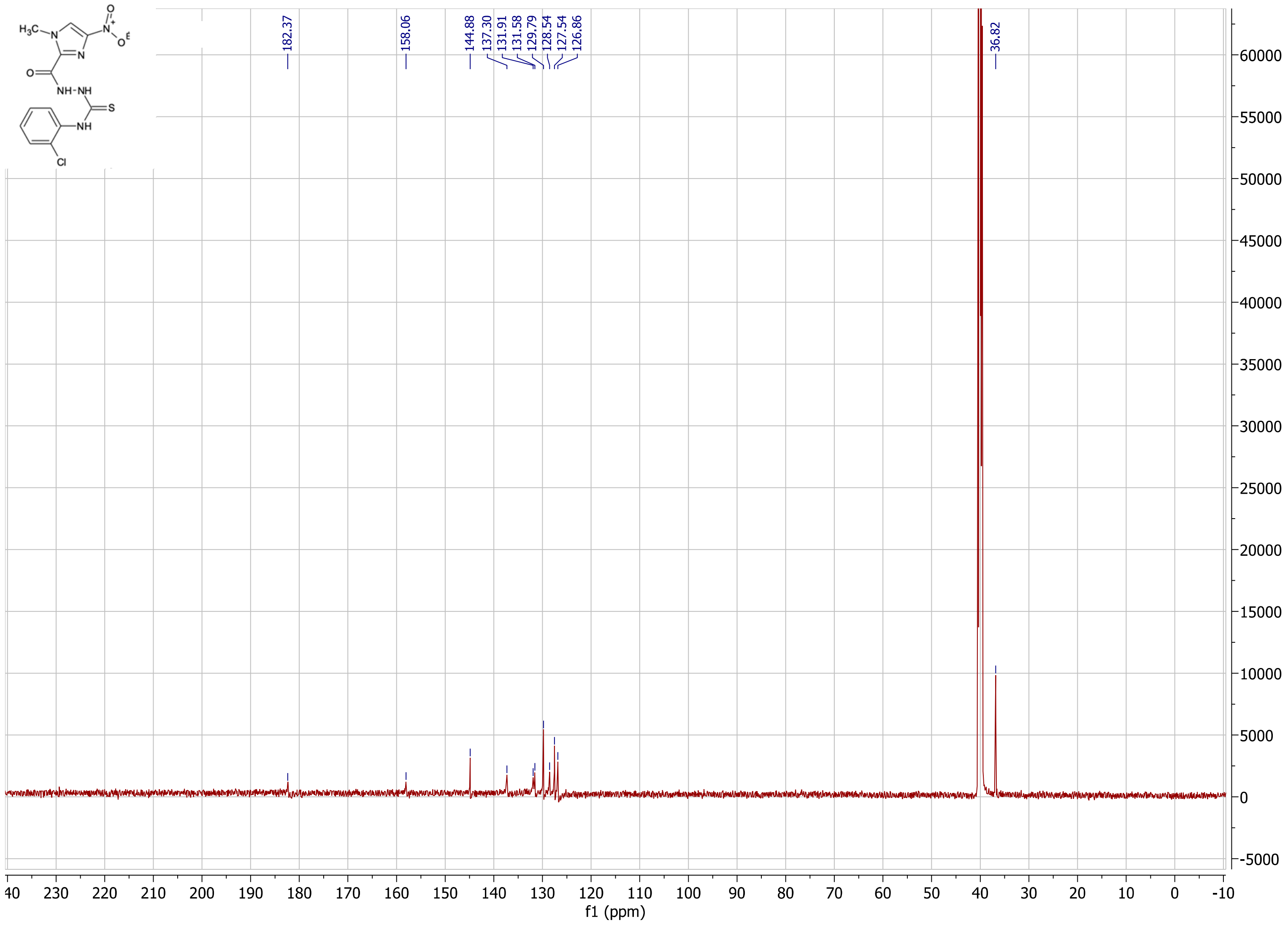

Figure S62. The ^{13}C NMR of compound **3.**

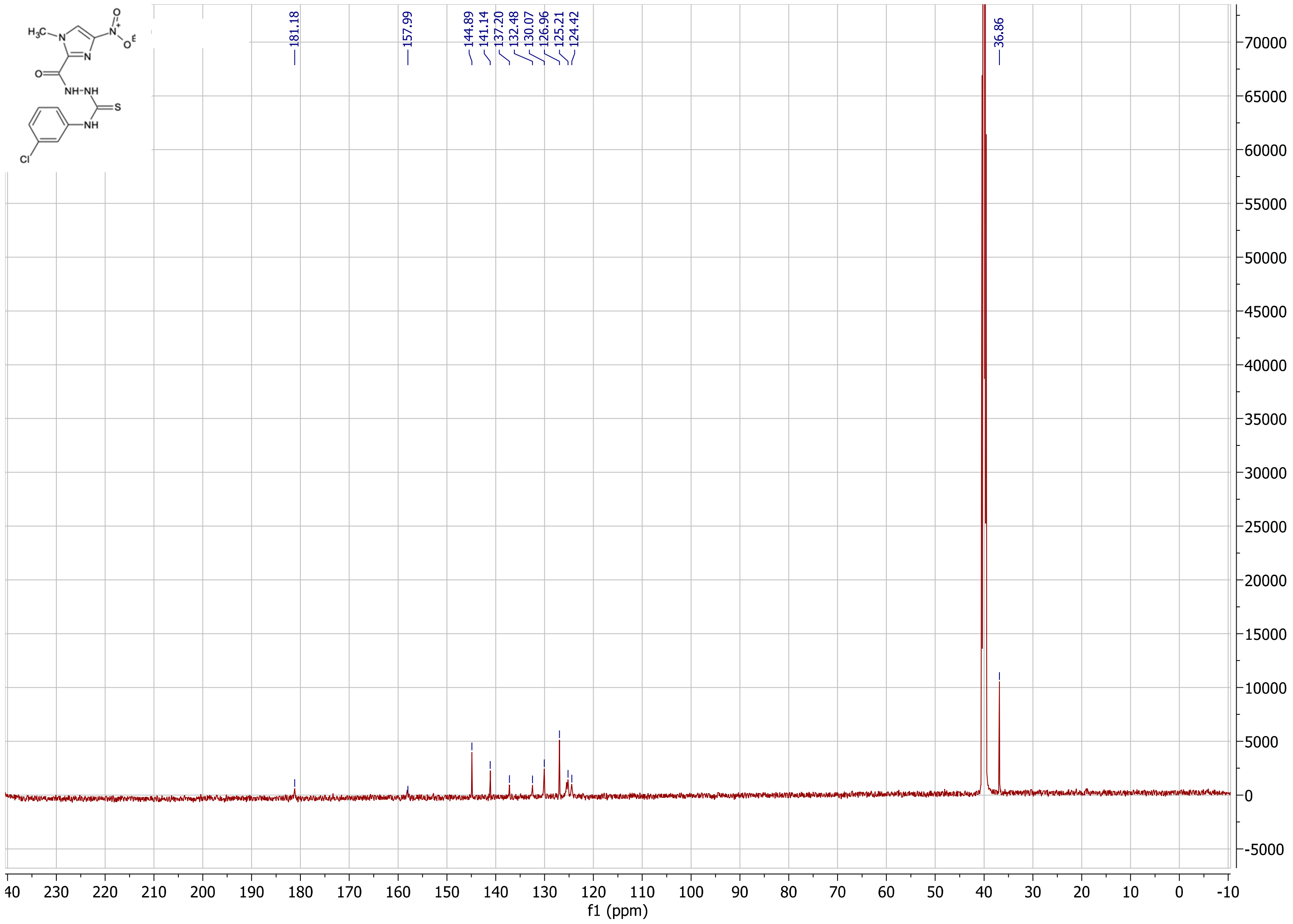

Figure S63. The ^{13}C NMR of compound **4.**

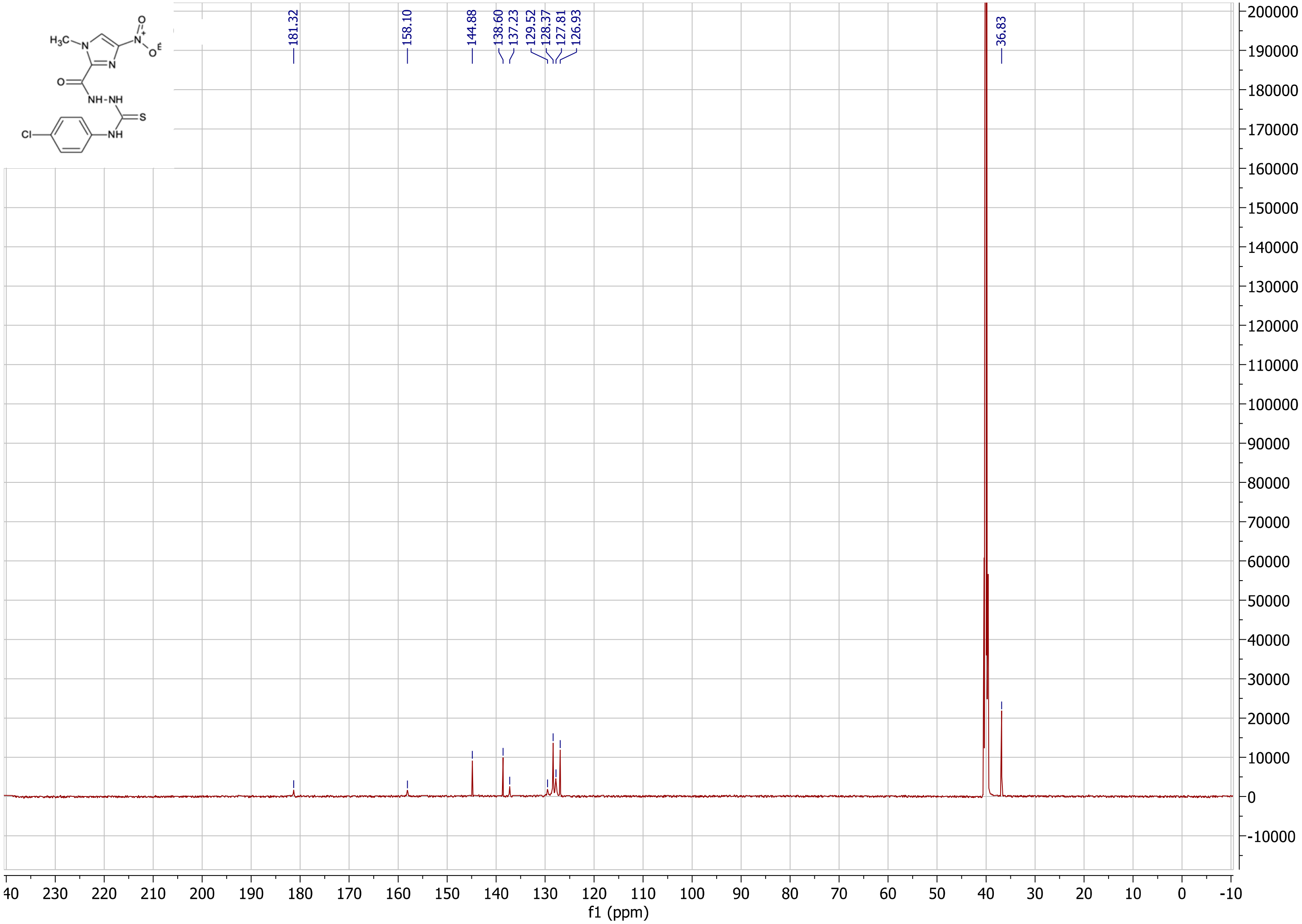

Figure S64. The ^{13}C NMR of compound **5.**

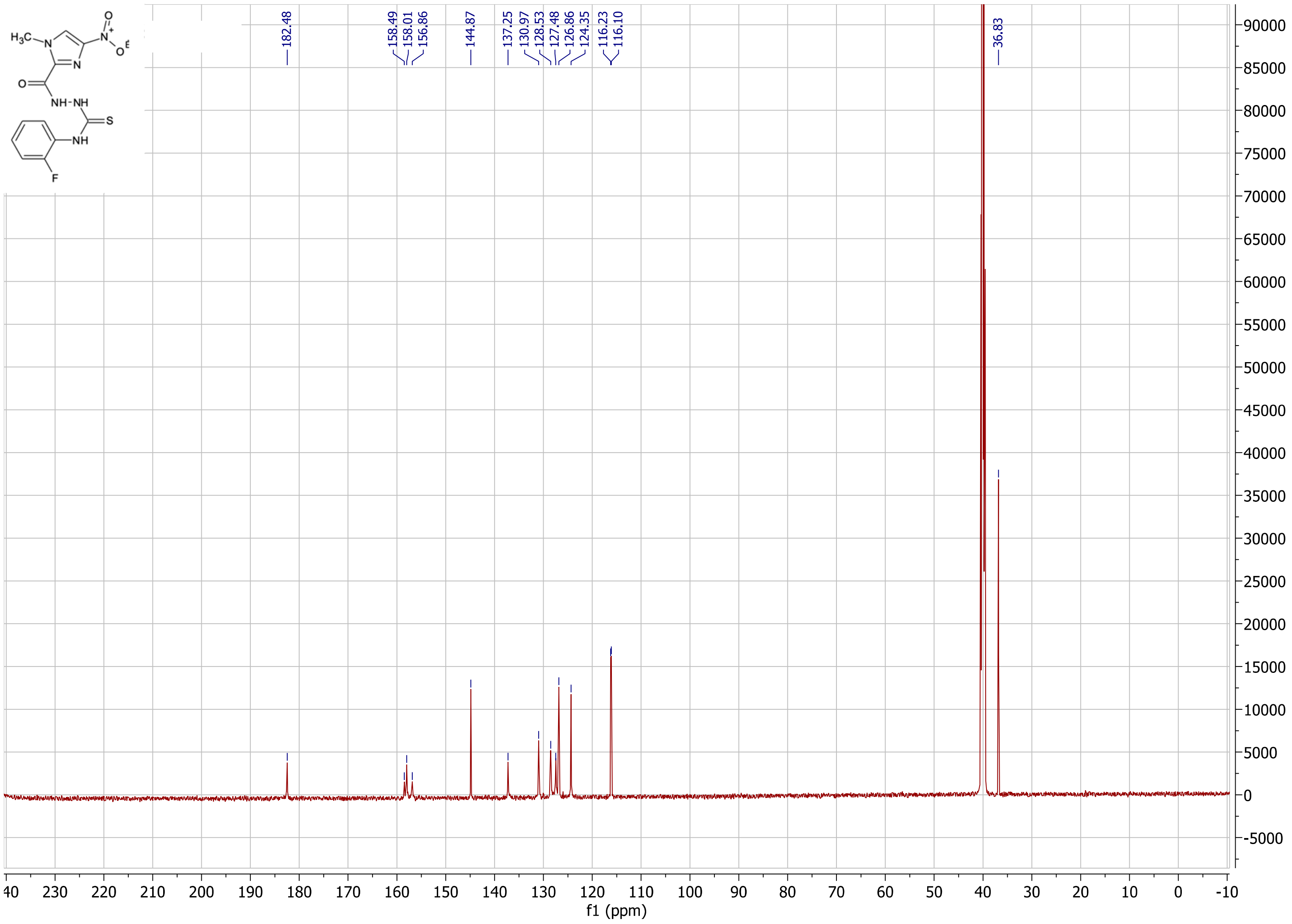

Figure S65. The ^{13}C NMR of compound **6.**

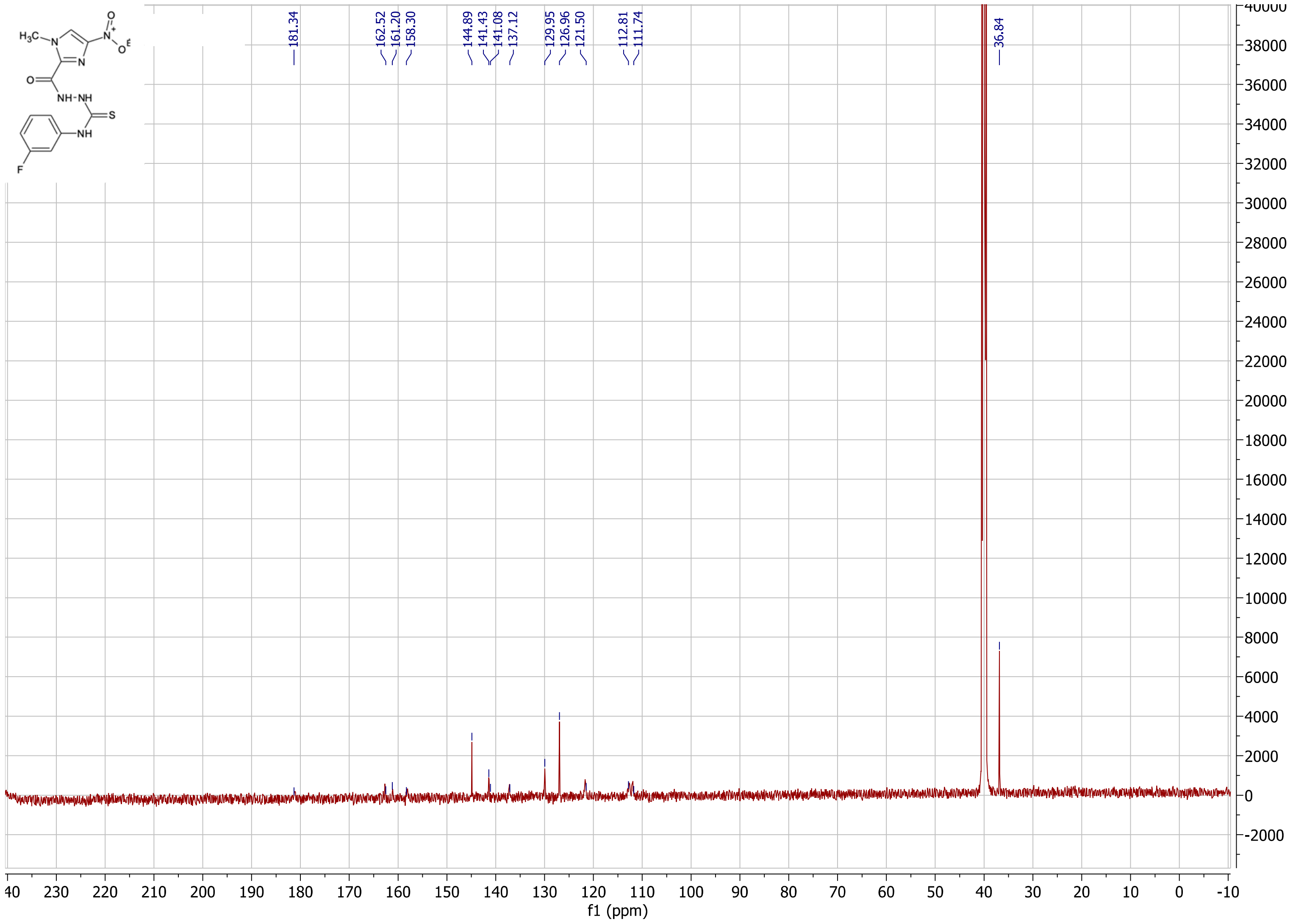

Figure S66. The ^{13}C NMR of compound **7.**

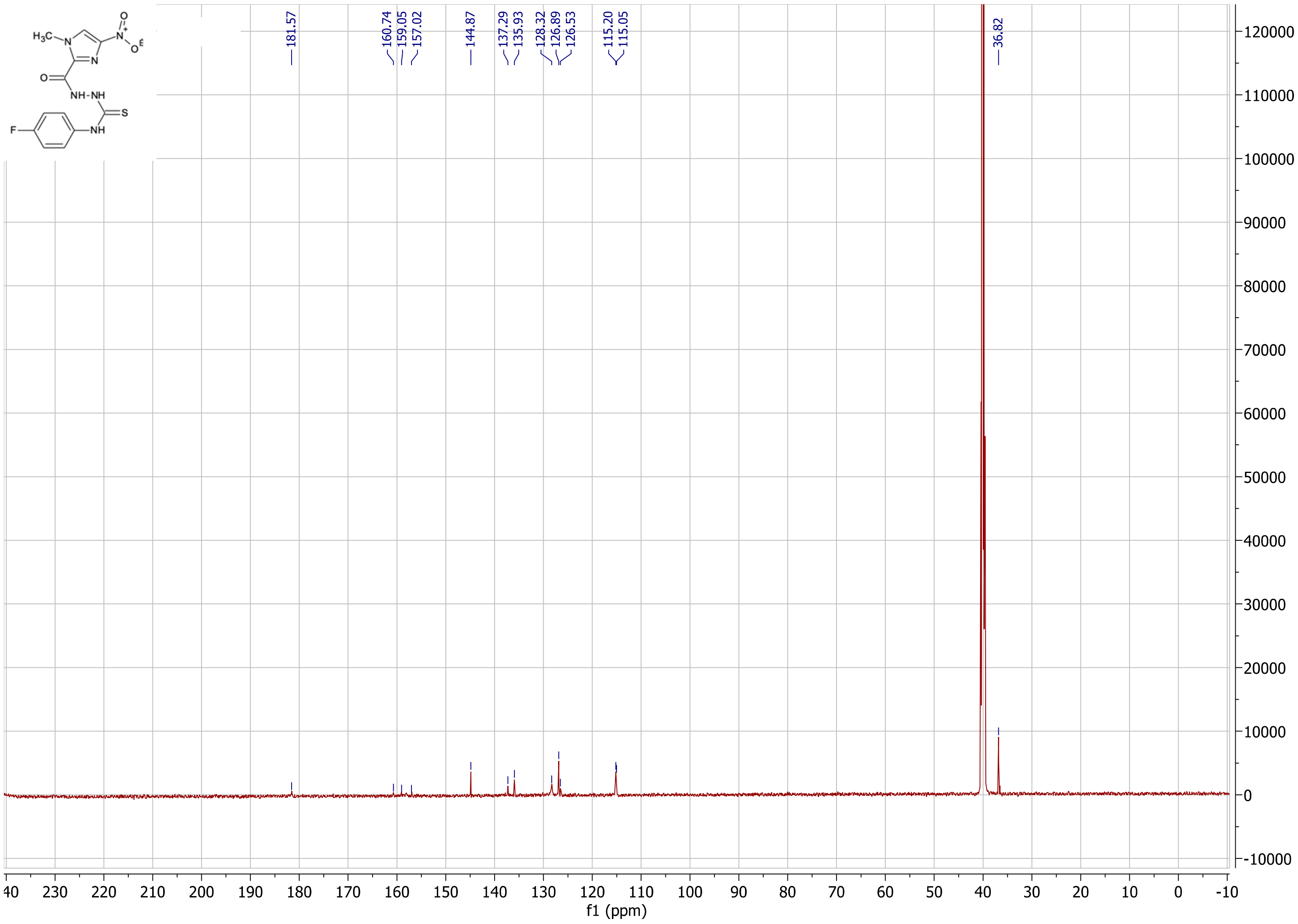

Figure S67. The ^{13}C NMR of compound **8.**

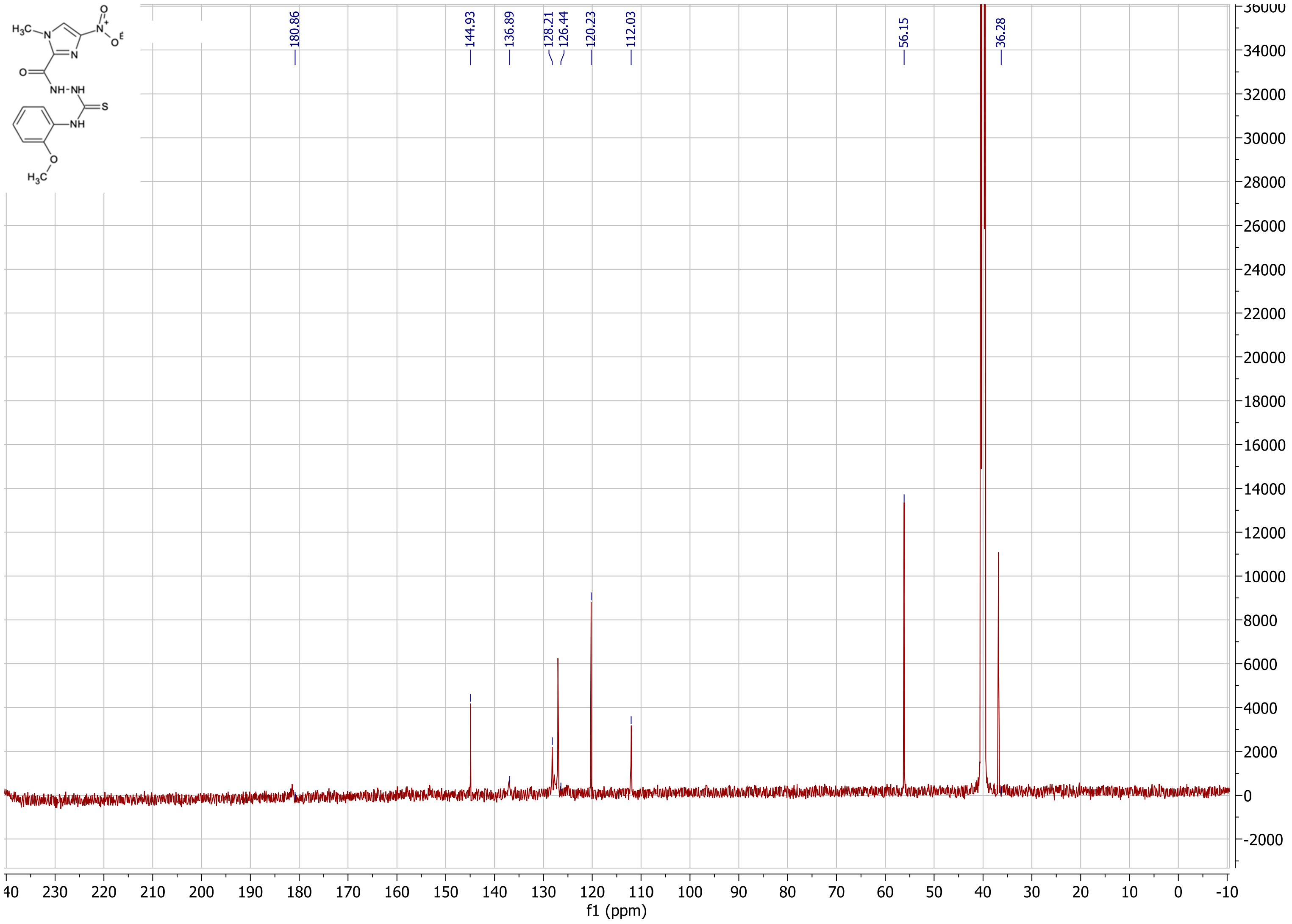

Figure S68. The ^{13}C NMR of compound **9.**

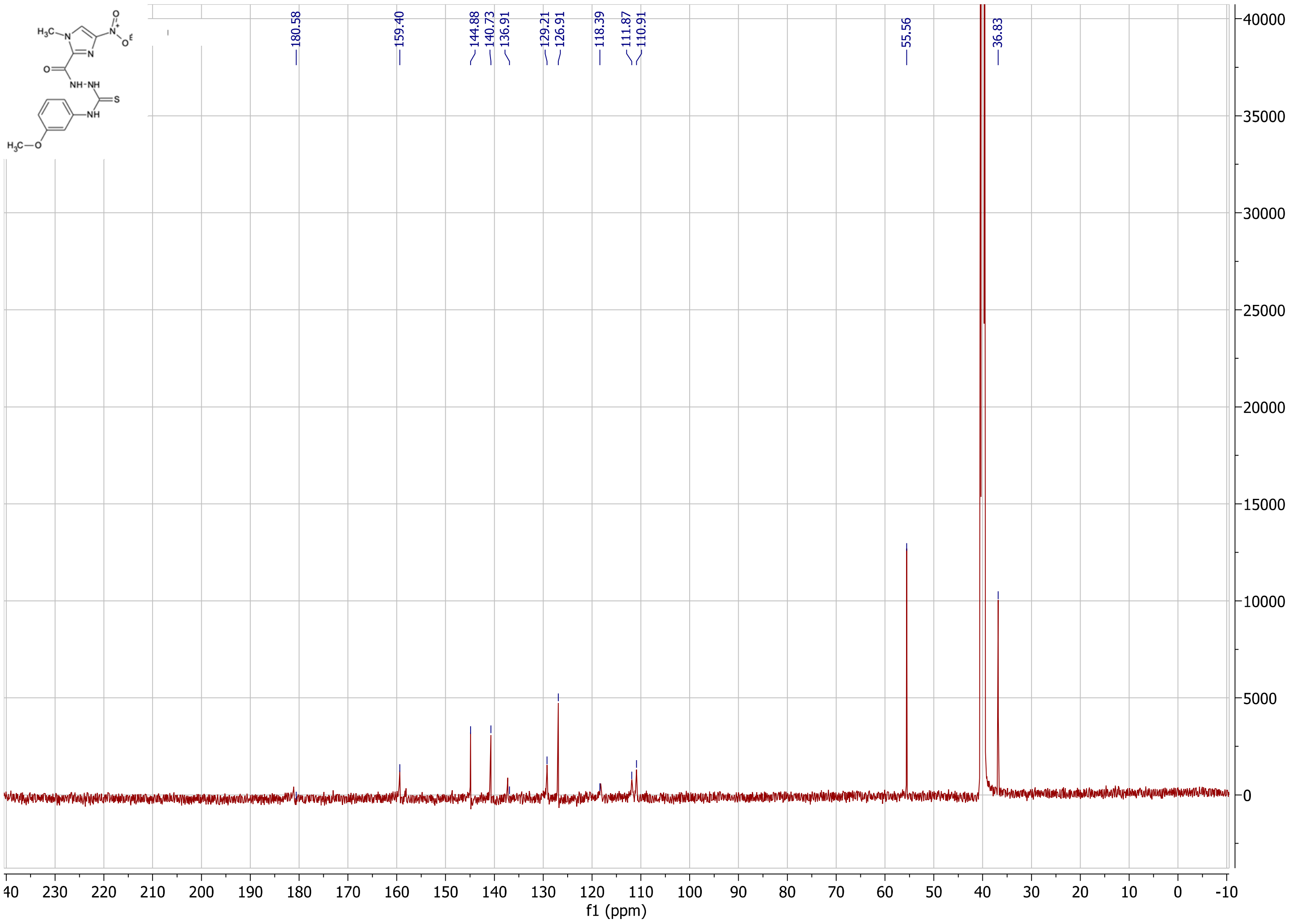

Figure S69. The ^{13}C NMR of compound **10.**

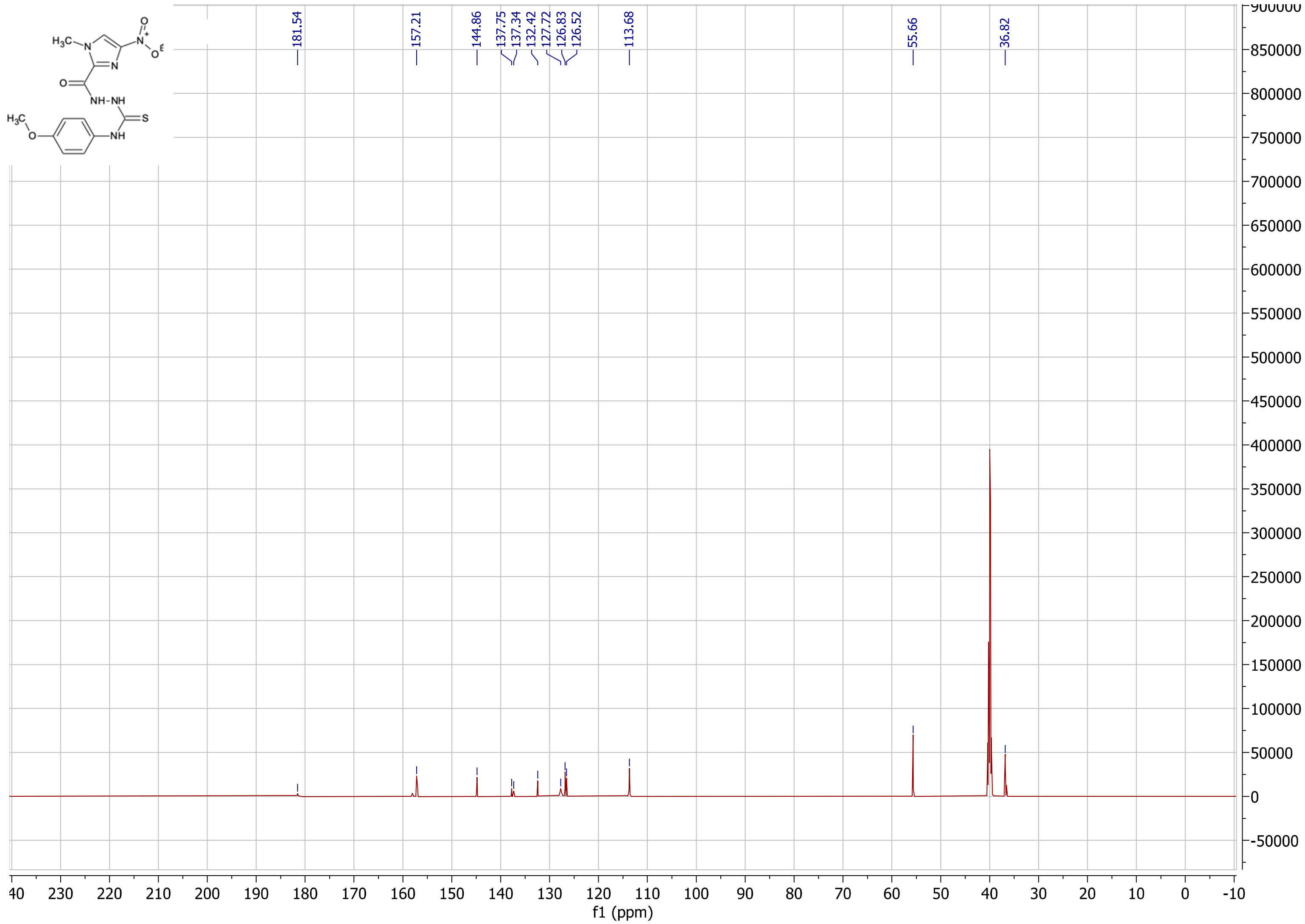

Figure S70. The ^{13}C NMR of compound **11.**

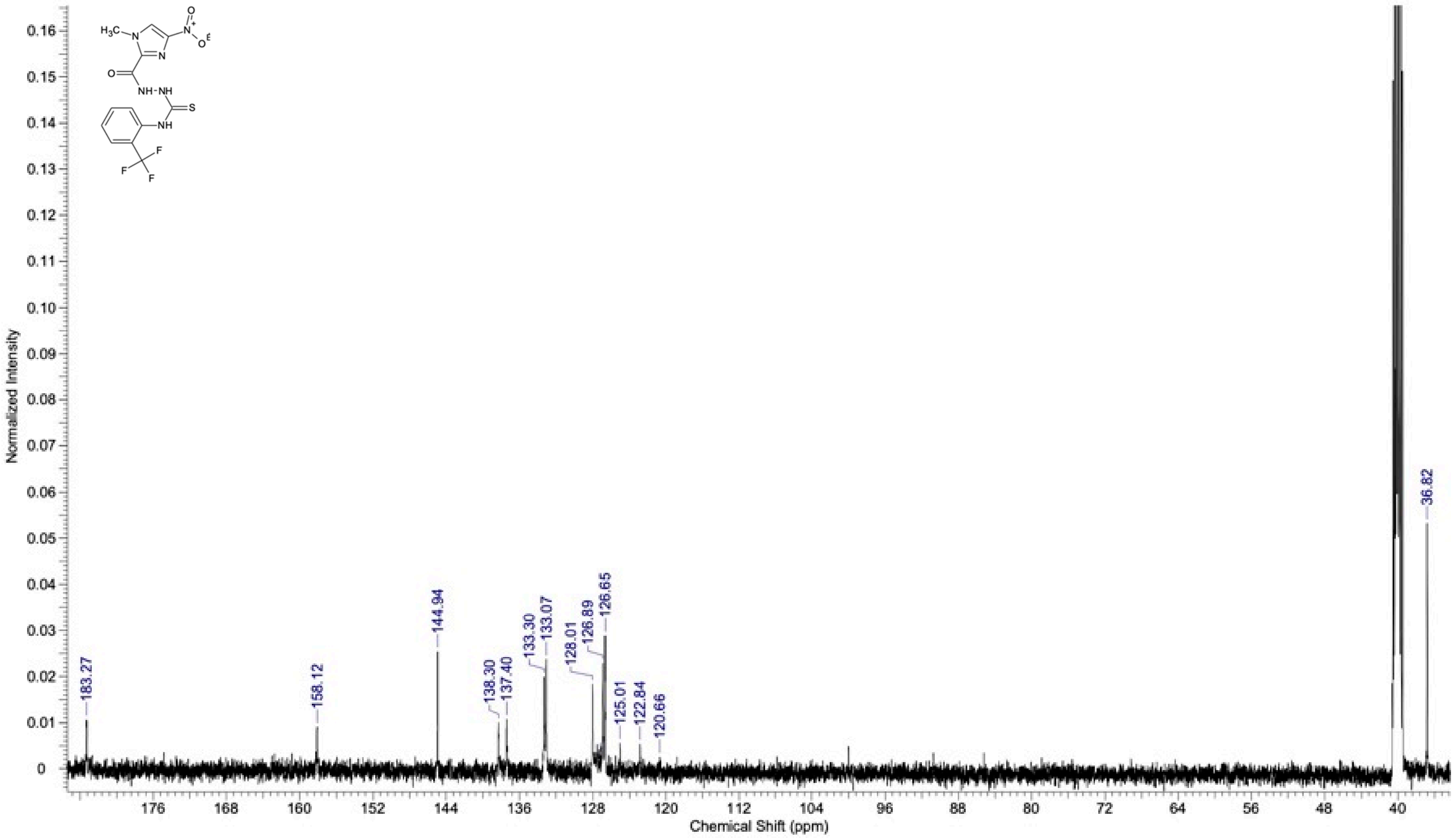

Figure S71. The ^{13}C NMR of compound **12.**

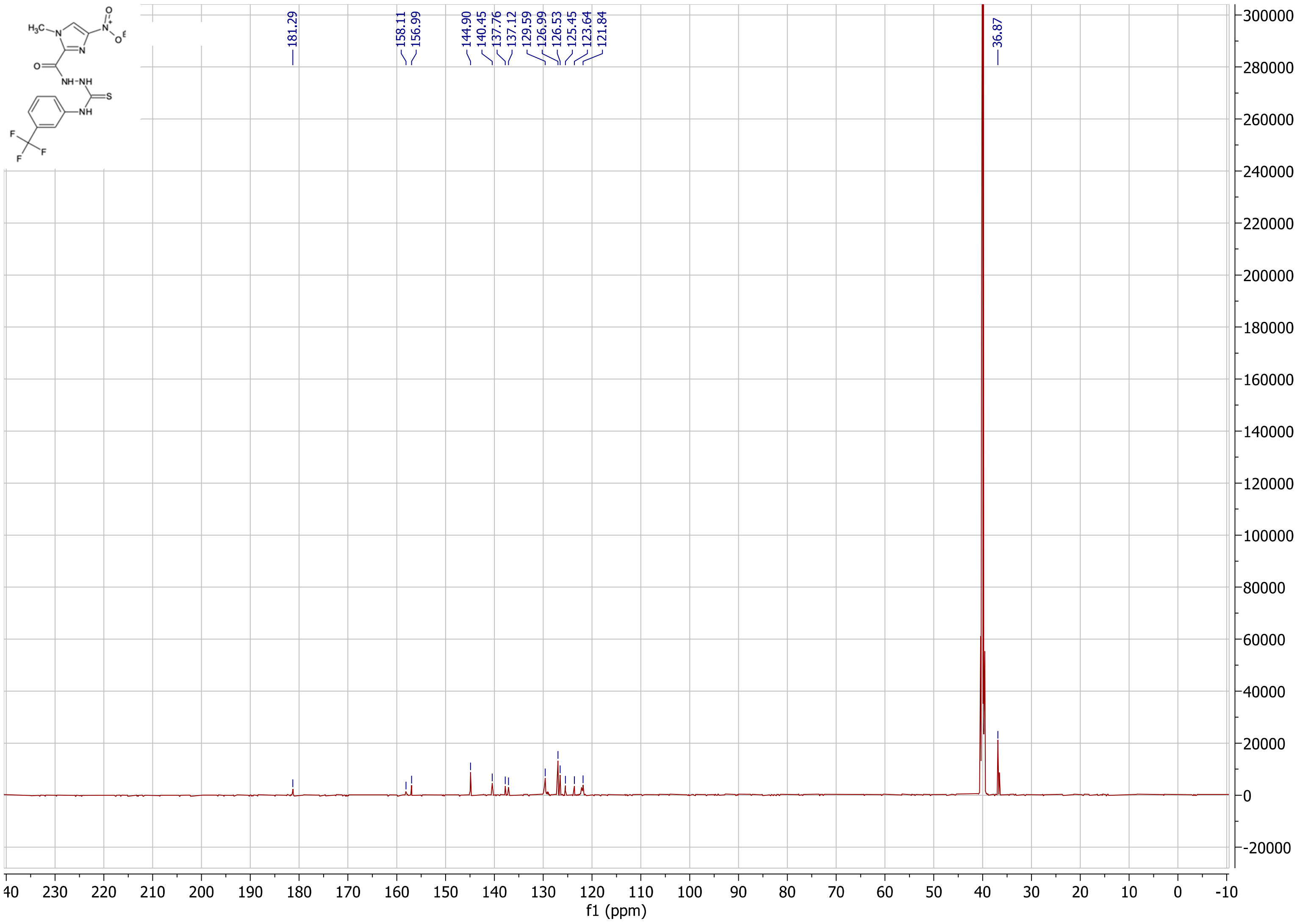

Figure S72. The ^{13}C NMR of compound **13.**
